# A Useful Synthesis of 2-Acylamino-1,3,4-oxadiazoles from Acylthiosemicarbazides Using Potassium Iodate and the Discovery of New Antibacterial Compounds

**DOI:** 10.3390/molecules24081490

**Published:** 2019-04-16

**Authors:** Tianlei Li, Gang Wen, Jishun Li, Wenxuan Zhang, Song Wu

**Affiliations:** 1 State Key Laboratory of Bioactive Substance and Function of Natural Medicines, Institute of Materia Medica, Peking Union Medical College and Chinese Academy of Medical Sciences, Beijing 100050, China; litianlei818@163.com (T.L.); wengang@imm.ac.cn (G.W.); 15286037616@163.com (J.L.); wxzhang@imm.ac.cn (W.Z.); 2 State Key Laboratory of Functions and Applications of Medicinal Plants, Guizhou Medical University, Guiyang 550014, China; 3 School of Pharmacy, Guiyang College of Traditional Chinese Medicine, Huaxi University town, Guiyang, Guizhou 550025, China

**Keywords:** heterocycles, synthetic method, 2-acylamino-1,3,4-oxadiazoles, antibacterial

## Abstract

A useful method for the synthesis of 2-acylamino-1,3,4-oxadiazoles was developed. By using potassium iodate as an oxidant in water at 60 °C, a wide range of 2-acylamino-1,3,4-oxadiazoles were afforded in moderate to excellent yields within two hours. This method could provide a facile shortcut to generate a series of 2-acylamino-1,3,4-oxadiazoles in medicinal chemistry. Interestingly, some highly potent antibiotic compounds were found through this synthetic method, and some of them displayed a significant improvement in activity compared with the corresponding 1,4-diacylthiosemicarbazides. Compound **2n** was the most active against *Staphylococcus aureus* with MIC (minimum inhibitory concentration) of 1.56 mg/mL, and compounds **2m** and **2q** were the most active against *Bacillus subtilis* with MIC of 0.78 mg/mL. The preliminary cytotoxic activities of the most potent compounds **2m**, **2n**, and **2q** against the androgen-independent (PC-3) prostate cancer cell line were more than 30 μM (IC_50_ > 30 μM).

## 1. Introduction

**Figure 1:**
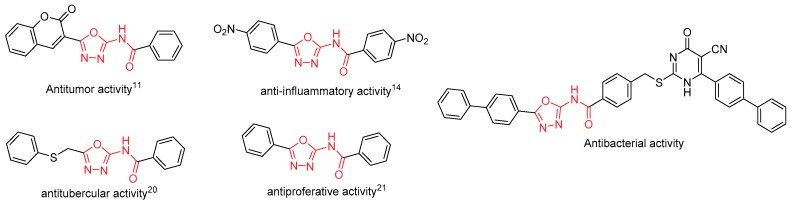
Biologically active compounds containing the 2-acylamino-1,3,4-oxadiazole core motif.

1,3,4-Oxadiazole, a pivotal five-membered nitrogen-containing heterocycle, is an interesting pharmacophore in medicinal chemistry. In particular, 2-acylamino-1,3,4-oxadiazoles have a broad range of biological activities such as anticoagulant [1,2,3], antibacterial [4,5,6,7,8], antifungal [9,10], anticancer [11,12], anti-inflammatory [13,14,15], insecticidal [16,17], antihypertensive [18,19], etc. [20,21] (Figure 1). Thus, the development of facile and efficient approaches to access these 2-acylamino-1,3,4-oxadiazoles is highly valuable to early drug discovery.

**Scheme 1:**
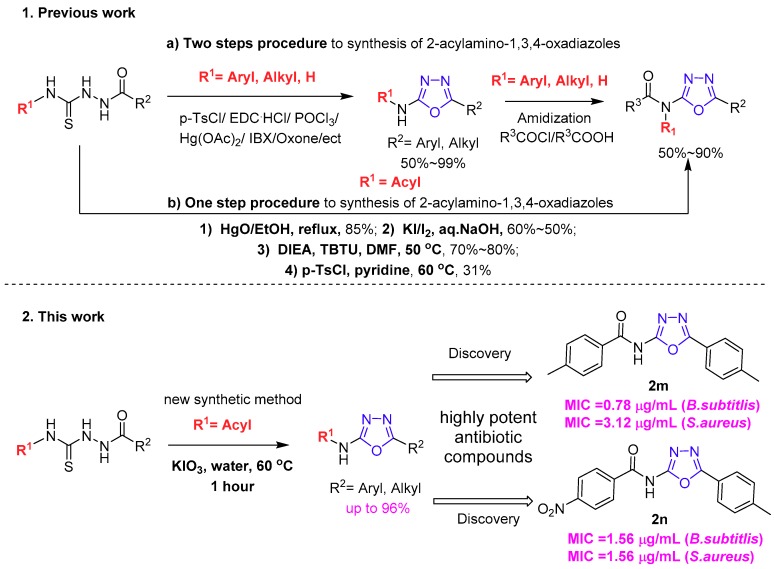
Intramolecular cyclodesulfurization of thiosemicarbazides. **1**. The previous methods for the synthesis of 2-acylamino-1,3,4-oxadiazoles: (**a**) Two-step procedure to synthesize 2-acylamino-1,3,4-oxadiazoles by cyclization and amidization [22,23,24,25,26,27,28,29,30,31,32,33,34,35]; (**b**) One-step procedure to synthesize 2-acylamino-1,3,4-oxadiazoles by desulfurated cyclization [11,36,37,38,39,40,41]. **2**. Our new method for the preparation of 2-acylamino-1,3,4-oxadiazoles and the discovery of new antibacterial compounds.

To date, different methods have been developed for the synthesis of these heterocyles. The most straightforward synthesis relies on the cyclization of corresponding thiosemicarbazide derivatives with several desulfurating agents [22], such as 1,3-dibromo-5,5-dimethylhydantoin [23]; *p*-tosyl chloride [24,25]; alkylating agents [26,27], such as methyl iodide and ethyl bromoacetate; carbodiimides [28,29]; mercury (II) acetate [30,31,32]; oxone [33]; and POCl_3_ [34] (Scheme 1).

Recently, Sureshbabu and Akamanchi [35] independently utilized hypervalent iodine (IBX) as an oxidant to prepare 2-amino-1,3,4-oxadizale under mild conditions. Chang [36] reported a one-pot procedure to synthesize 2-amino-substituted 1,3,4-oxadiazoles via the condensation of semicarbazide and the corresponding aldehydes followed by I_2_-mediated oxidative cyclization. As is well known, all of the mentioned methods are extremely dependent on the *N*-substituted group property, and when the *N*-substituted groups are aryl, hydrogen, and alkyl groups, the reaction can occur smoothly. When the *N*-substituted groups are replaced by the acyl groups, these reactions often lead to undesirable by-products. In fact, the preparation of 2-acylamino-substituted 1,3,4-oxadiazoles through the direct oxidative cyclization of thiosemicarbazide is still limited. For example, the 2-acylamino-substituted 1,3,4-oxadiazoles were generated through the cyclodesulfurization of thiosemicarbazide in the presence of I_2_ /aqueous NaOH as a desulfurization reagent with fair yields [37,38]. Other desulfurization reagents such as stoichiometric mercuric salts [11,39], or lead oxide [40] can be used to induce the cyclization to form the 2-acylamino-substituted 1,3,4-oxadiazoles. More recently, Balalaie and co-workers [41] proved that *O*-(benzotriazol-1-yl)-*N,N,N′,N′*-tetramethyluronium tetrafluoroborate (TBTU) as a uronium coupling reagent can selectively activate the sulfur moiety to construct disubstituted 1,3,4-oxadiazoles with good yields (Scheme 1). However, these methods suffer from several limitations, such as the handing of harsh and toxic reagents, a poor functional group tolerance, multistep synthetic steps, a long reaction time, and a tedious purified process.

Herein, we reported a useful method for the synthesis of 2-acylamino-1,3,4-oxadiazoles by the direct oxidation of acylthiosemicarbazides using potassium iodate under mild conditions. A wide range of 2-acylamino-substituted 1,3,4-oxadiazoles were afforded in moderate to excellent yields with a short reaction time. We believe that this useful approach provides a facile shortcut for the construction of diverse 2-acylamino-substituted 1,3,4-oxadiaxole libraries in a high throughput manner.

## 2. Results and Discussion

### 2.1. Optimization of the Reaction Conditions

We commenced with the reaction of 4-methyl-*N*-[2-(3-nitrobenzoyl)hydrazine-1-carbonothioyl]benzamide (**1a**) in the presence of 1.5 equivalent potassium persulfate or ammonium persulfate as the oxidant in water at 100 °C, but the reaction did not give the corresponding product (Table 1, entry 1–2). Switching to other oxidants such as 2-iodoxybenzoic acid (IBX) and oxone, the cyclodesulfurization product was observed with a low conversion (entry 3–4). To our delight, the reaction obtained the cyclodesulfurization product as 2-acylamino-1,3,4-oxadiazole in 53% yield by using KIO_3_ as the oxidant (Table 1, entry 5). An encouraging result of an 83% isolated yield was afforded (entry 6) when the reaction temperature was deceased to 80 °C. The screening of different temperatures showed that the reaction temperature had a great influence on this oxidation reaction. The reaction could be performed at a relatively low temperature (60 °C); the isolated yield of the reaction was up to 90% (Table 1, entry 8). Subsequently, various aprotic solvents, such as dichloromethane (DCM) and acetone, were investigated in the presence of 1.5 equivalent KIO_3_, and the reaction was fully inhibited when the reaction was carried out in DCM and acetone. Thus, we concluded that the optimal condition was the use of KIO_3_ as the oxidant and water as the solvent at 60 °C (entry 8).

### 2.2. Exploring the Substrates’ Scope

With the optimized reaction condition in hand, we next investigated the scope of 1,4-diacylthiosemicarbazides and the results are depicted in Table 2. Different substituents were well-tolerated and most of the products were synthesized in high yields. The effects of the R_2_ groups’ properties are important in the cyclization reaction. When the R_2_ groups were the phenyl substituents, the oxidative reaction resulted in higher yields than the alkyl groups (**2b**–**e**, **2i**–**k, 2n, 2o**). Additionally, higher yields of the 2-acylamino-1,3,4-oxadiazoles were observed when the phenyl group bearing an electron-withdrawing substituent (-NO_2_) was presented on the R_2_ group (**2i**, **2j**, **2k**). Furthermore, the reaction exhibited a good tolerance with the R_1_ groups in the 1,4-diacylthiosemicarbazides substrates (**1b**–**e**, **1i**, **1k**). When the R_1_ groups were replaced by a phenyl group, the reaction could generate the desired products with moderate yields (**2c**, **2f**, **2l**). A large-scale reaction was conducted to assess the scalability of this desulfurization and cyclization reaction. Under the standard conditions, the thiosemicarbazide **1a** (3.0 mmol, 1.07 g) was smoothly converted into the desired products with a 90% yield (See Supplementary Materials).

**Scheme 2:**
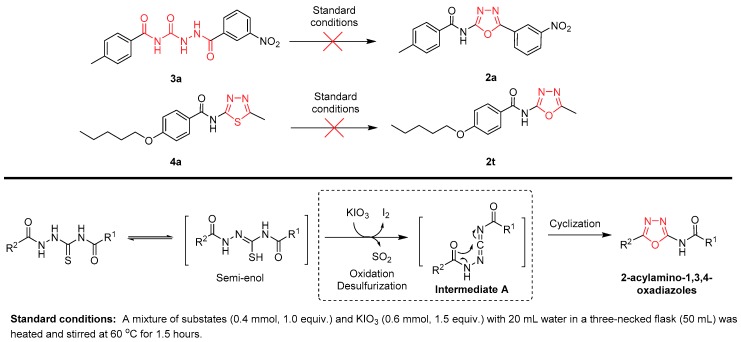
Postulated mechanism of desulfurization for the synthesis of 2-acylamino-1,3,4-oxadiazoles.

To gain an insight into the reaction mechanism, we set out the control reaction with the semicarbazide (**3a**) and 2-acylamino-1,3,4-thiadiazoles (**4a**) under the standard conditions to validate the reaction process. However, neither of them could be converted into the desired 2-acylamino-1,3,4-oxadiazoles (**2a**, **2t**) (Scheme 2). This may suggest that the oxidation and desulfurization step is the key step in this process. Recent reports have proposed that the reaction may be initiated by the nucleophilic attack of *S* on the electrophilic iodine of IBX, and subsequently, the cyclization and desulfurization step occurred at the same time [42,43]. Very recently, Frederick and co-workers [44] observed that 1-(2-hydroxybenzoyl)-thiosemicarbaziders existed as the keto–enol equilibrium in the ^1^H-NMR spectrum, and similar results have already been mentioned by Mostafa [45]. However, the details of the corresponding oxidation and desulfurization mechanism remain to be ascertained in this new reaction. On the basis of our studies and recent reports [35,42,43,44,45], a possible mechanism for this reaction is illustrated in Scheme 2. Due to the keto–enol equilibrium of thiosemicarbaziders, hydrosulphonyl could be oxidized by KIO_3_ to form sulfur dioxide which could be released from the corresponding semi-enol form. An activated carbodiimide intermediate A, generated from the thiosemicarbazides, could subsequently occur to give the desired 2-acylamino-1,3,4-oxiadiazoles. In this reaction, elemental iodine was isolated, and we found that the pH of the reaction solution was less than 6 (pH = 5–6) after the reaction. The keto–enol equilibrium of thiosemicarbaziders could be promoted by the increased reaction temperature, but the higher temperature may induce the decomposition of 2-acylamino-1,3,4-oxadiazoles. Furthermore, the substituted R_2_ group had a significant effect on this reaction, and when the phenyl groups were replaced by the alkyl groups, the yields were dramatically decreased (**2d**, **2e**, **2g**, and **2h**). It may suggest that the carbodiimide intermediate A could be stabilized by the phenyl groups. The reaction has a high tolerance for the property of the substituted R_1_ groups (**2o**–**q**).

### 2.3. Evaluation of the Antibacterial Activity

The discovery of highly proficient chemical reactions that give functionalized bioactive heterocycles with interesting pharmacophoric properties is a major challenge of medicinal chemistry [46,47]. The development of new synthetic methods for the construction of new scaffolds is an efficient shortcut to accelerate the rate of lead molecule identification [48]. The development of bacterial resistance to currently available antibacterial agents is a growing global health problem [49,50]. The ESKAPE pathogens (*Enterococcus faecium*, *Staphylococcus aureus*, *Klebsiella pneumoniae*, *Acinetobacter baumannii*, *Pseudomonas aeruginosa*, and *Enterobacter* species) are the cause of severe and difficult-to-treat nosocomial infections due to their antibiotic resistance [50]. One of the most important solutions is the discovery of new antibacterial compounds with a novel mechanism in medicinal chemistry. In our laboratory, we are focusing on the development of new antibacterial agents to protect against the ESKAPE pathogens [51]. As a continuation of our previous work, we further evaluated their antibiotic activity against *B. subtilis*, *S. aureus* (Gram-positive bacteria) and *E. coli* (Gram-negative bacteria) and the results are summarized in Table 3. By comparing the activities of the thiosemicarbazide (**1a**–**1s**) and the 2-acylamino-1,3,4-oxadiazoles (**2a**–**2s**) against *B. subtilis*, the potency of the major products (**2a**–**2s**) have apparently improved. The activity of the compounds **2e**, **2k**, **2m**, **2n**, **2q**, and **2s** showed six times the potency of their corresponding 1,4-diacylthiosemicarbazides. Furthermore, the compound **2q** even showed 64 times the potency of its 1,4-diacylthiosemicarbazides. There were another five compounds, namely **2a**, **2j**, **2l**, **2p**, and **2r**, which also exhibited better activity. As for antibiotic activity against *S. aureus*, although some products displayed lower activities, compounds **2e** and **2q** were also 32 times better than compounds **1e** and **1q.** We found that the most potent compounds of all of the compounds **1a**–**1s** and **2a**–**2s** were **2m**, **2n**, and **2q**. However, all of the compounds **1a**–**1s** and **2a**–**2s** did not show good antibiotic activity against *E. coli* (Gram-negative bacteria). At the moment, there is no clear trend regarding activity against *E. coli*, and the most active compounds (**2m**, **2n**, and **2q**) against Gram-positive pathogens do not show the same trend of activity against Gram-negative bacteria. In the literature, some of the 1,3,4-oxadiazoles have been reported to possess cytotoxic activities (PC-3 cell line) [21]. We tested the cytotoxicity of the most potent compounds **2m**, **2n**, and **2q** against the human tumor cell line (androgen-independent prostate cancer cell line, PC-3) to evaluate their potential as antibacterial drugs. The results showed that the in vitro IC_50_ (median growth inhibitory concentration) of **2m**, **2n**, and **2q** was more than 30 μM (IC_50_ > 30 μM). Their lower cytotoxic activities may suggest that the compounds **2m**, **2n**, and **2q** are interesting drug-like antibacterial molecules.

## 3. Materials and Methods

### 3.1. Reagent and Methods

Reagents: unless otherwise indicated, all solvents and organic reagents were obtained from commercially available sources and were used without further purification. Acetone was dried by a molecular sieve. Potassium iodate (99%, Innochem) was used in this oxidative reaction.

Instrument: The reaction process was monitored by thin-layer chromatography (TLC, Yantai Jiangyou Co.Ltd, Tsingdao, Shandong, China) with silica gel plates (thickness = 0.20 mm, GF254) under UV light and LC-MS (Waters Acquity UPLC/SQD, Waters Acquity UPLC/SQD, Milford, MA, USA). Mass spectra were obtained using a Waters Acquity UPLC-SQD mass spectrometer. High-resolution mass spectra (HRMS, Thermo Scientific Exactive. Plus, 81 Wyman Street, Waltham, MA, USA) were recorded on an Agilent Technologies LC/MSD TOF spectrometer. The ^1^H-NMR spectra were recorded on a Varian Mercury 400 or 500 MHz instrument, and ^13^C-NMR spectra were recorded at 400 or 500 MHz on a Varian Mercury using dimethyl sulfoxide-*d*_6_ (DMSO-*d*_6_), methanol-*d*_4_ (CD_3_OD), chloroform-*d* (CDCl_3_) as the solvents and tetramethylsilane (TMS) as an internal standard.

### 3.2. Synthesis of 1,4-Diacylthiosemicarbazide Substrates *(**1a**–**1s**)*

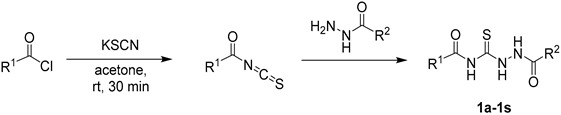



To a solution of acyl chloride (1.0 equiv.) in 20 mL dry acetone, potassium thiocyanate (1.1 equiv.) was added at room temperature. The solution was stirred at room temperature for 0.5 h. Then, relevant acylhydrazine (1.05 equiv.) was added. The reaction was stirred at 60 °C for 2 h until the reaction was complete based on TLC (thin-layer chromatography) (DCM:MeOH = 15:1). Then, it was cooled to room temperature, and concentrated under reduced pressure. The resulting residue was washed by water and recrystallized in MeOH to give the corresponding intermediate as a white solid.

### 3.3. Synthesis of 2-acylamino-1,3,4-oxadiazole substrates *(**2a**–**2s**)*

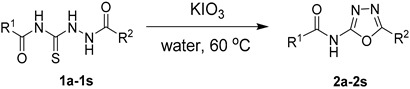



A mixture of 1,4-diacylthiosemicarbazides substrates (0.4 mmol, 1.0 equiv.) and KIO_3_ (0.6 mmol, 1.5 equiv.) with 20 mL water in a three-necked flask (50 mL) was heated and stirred at 60 °C for 1.5 h. After the reaction was completed, the reaction mixture was cooled to room temperature, and the precipitate was removed by filtration. The aqueous phase was extracted with EtOAc. The combined organic layers were dried by anhydrous Na_2_SO_4_, and concentrated under reduced pressure. The residue and the former precipitate were recrystallized in EtOAc to give the desired products.

### 3.4. Antibacterial Activity and Cell Viability Assay

Microorganisms used for the antibiotic assay were *S. aureus* ATCC 29213, *B. subtilis* ATCC 63501, and *E. coli* ATCC 25922. The MIC (minimum inhibitory concentration) of the compounds was determined according to the macrodilution broth method (National Committee for Clinical Laboratory Standards 2000) in Mueller–Hinton Broth (MHB, Difco, Detroit, MI, USA).

Stock solutions of all compounds were prepared in dimethylsulfoxide. The stock solution was then two-fold diluted in MHB to give an initial concentration of 100 μg/mL, and further dilution was performed until a final concentration of 0.05 μg/mL was obtained.

The microorganisms were incubated overnight in MHB at 37 °C and matched to a 0.5 MCFarland standard and added to make a final volume of 200 μL in each microliter well. A volume of bacterial suspension equal to the volume of diluted antimicrobial solution was added to each well of antimicrobial agent [52]. The 96-well plates were incubated for 18 h under aerobic conditions. The MIC was recorded as the lowest concentration of the test compound which inhibited the growth of bacteria in the broth. Standard drug, media, and bacterial growth control wells were included in each plate. Levofloxacin was used as a positive control. The assay was done in duplicate to confirm results.

The PC-3 cells were kindly provided by the cell bank of the Chinese Academy of Sciences and were maintained in F12K (Wisent) supplemented with 10% fetal bovine serum (Biosera) and 1 unit/mL penicillin−streptomycin (Wisent) at 37 °C under humidified conditions.

Cytotoxicity assays: The in vitro cytotoxicity of the compounds (**2m**, **2n**, and **2q**) against PC-3 cells (the androgen-in) was measured by the MTT assays. This assay was based on the cleavage of the yellow tetrazolium salt [3-(4,5-dimethyl-thiazol-2yl]-2,5-diphenyl-tetrazolium bromide, MTT, Sigma) forming purple formazan crystals by viable cells [53]. The cells were plated in 96-well culture plates at a density of 3 × 10^3^ cells per well and incubated for 24 h at 37 °C in a 5% CO_2_ incubator. The compounds (**2m**, **2n**, and **2q**) were dissolved in DMSO and diluted with culture medium. A total of 20 μL of the compound solution was added 24 h post-plating at the indicated concentrations. Cell viability was determined 72-h post-compound treatment. The total number of viable cells was determined using the MTT (biosharp) with the Envision (PekinElmer).

## 4. Conclusions

To conclude, we have developed an efficient, convenient, and environmentally friendly method to synthesize 2-acylamino-1,3,4-oxadiazoles by the direct oxidation of 1,4-diacylthiosemicarbazides. This oxidative protocol has also shown good functional group tolerance. By mixing the substrates with KIO_3_, a series of 2-acylamino-1,3,4-oxadiazoles were obtained within two hours. Compared with the classical strategies, this reaction provides a direct approach to construct 2-acylamino-1,3,4-oxadiazoles. Various substituted 2-acylamino-1,3,4-oxadiazoles were synthesized, and evaluated for antibacterial activity. The screening of those compounds revealed that several compounds were active against *B. subtilis* and *S. aureus*. The compound **2n** is the most active compound with MIC of 1.56 mg/mL against *S. aureus*, and the compounds **2m** and **2q** are the most active compounds with MIC of 0.78 mg/mL against *B. subtilis*. Interestingly, some of the 2-acylamino-1,3,4-oxadiazoles display a significant improvement in activity compared with the corresponding 1,4-diacylthiosemicarbazides. The preliminary cytotoxic effect of the most potent compounds **2m**, **2n,** and **2q** was determined by an MTT assay on an androgen-independent (PC-3) prostate cancer cell line. The results showed that the median growth inhibitory concentrations of **2m**, **2n**, and **2q** are more than 30 μM. Their lower cytotoxic activities may suggest that the compounds **2m**, **2n**, and **2q** are interesting drug-like antibacterial molecules. Further study of this project is currently underway in our laboratory.

## Figures and Tables

**Table 1:**
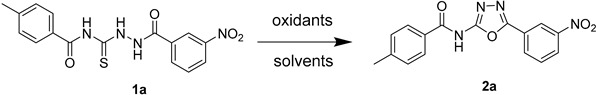
Optimization of cyclization of the thiosemicarbazide.

Entry	Oxidant	Solvent	Temp. (°C)	Yield (%)
1	K_2_S_2_O_8_	Water	100	N.O. ^a,c^
2	(NH_4_)_2_S_2_O_8_	Water	100	N.O. ^a,c^
3	IBX	Water	100	<30 ^a^
4	Oxone	Water	100	<40 ^a^
5	KIO_3_	Water	100	53 ^b^
6	KIO_3_	Water	80	83 ^b^
7	KIO_3_	Water	40	46 ^b^
8	KIO_3_	Water	60	90 ^b^
9	KIO_3_	DCM	60	5 ^a^
10	KIO_3_	Acetone	60	N.O. ^a,c^

All the reaction were carried out on a 0.4 mmol scale with 1.5 equiv. oxidants within 2 h; ^a^ the reaction conversions were monitored by LC-MS; ^b^ Isolated yield; ^c^ N.O.: No observation.

**Table 2:**
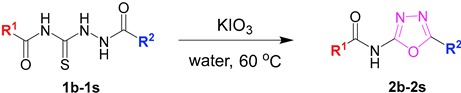
Reaction scope of cyclization of the thiosemicarbazide.

Entry	R^1^	R^2^	Starting Material	Product	Yield (%)
1	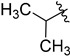	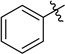	**1b**	**2b**	89
2	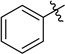	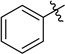	**1c**	**2c**	65
3	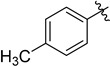	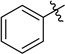	**1d**	**2d**	90
4	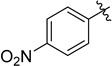	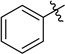	**1e**	**2e**	95
5	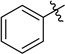	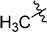	**1f**	**2f**	50
6	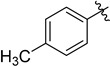	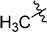	**1g**	**2g**	57
7	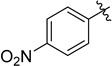	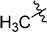	**1h**	**2h**	62
8	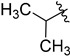	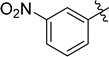	**1i**	**2i**	90
9	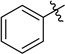	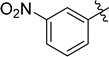	**1j**	**2j**	96
10	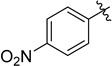	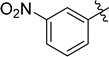	**1k**	**2k**	94
11	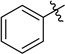	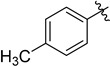	**1l**	**2l**	68
12	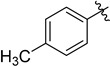	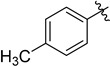	**1m**	**2m**	70
13	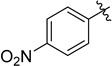	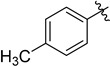	**1n**	**2n**	91
14	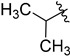	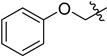	**1o**	**2o**	93
15	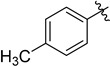	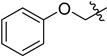	**1p**	**2p**	91
16	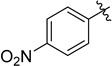	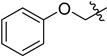	**1q**	**2q**	92
17	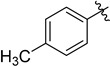	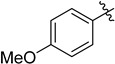	**1r**	**2r**	90
18	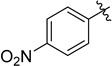	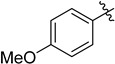	**1s**	**2s**	93

**Table 3:** The antibiotic activity of compounds **1a**–**1s** and **2a**–**2s.**

Comp. ID	MIC (μm/mL)			Comp. ID	MIC (μm/mL)		
	*B.subtilis*	*S. aureus*	*E. coli*		*B.subtilis*	*S. aureus*	*E. coli*
**1a**	12.5	12.5	>100	**2a**	3.12	6.25	>100
**1b**	12.5	25	50	**2b**	50	25	>100
**1c**	6.25	6.25	25	**2c**	6.25	25	100
**1d**	6.25	3.12	>100	**2d**	3.12	12.5	>100
**1e**	25	100	>100	**2e**	1.56	3.12	>100
**1f**	25	50	12.5	**2f**	100	>100	100
**1g**	12.5	25	12.5	**2g**	100	>100	100
**1h**	>100	>100	>100	**2h**	50	100	100
**1i**	25	50	>100	**2i**	25	100	>100
**1j**	25	25	>100	**2j**	6.25	25	>100
**1k**	50	100	>100	**2k**	1.56	6.25	>100
**1l**	3.12	6.25	>100	**2l**	1.56	6.25	>100
**1m**	6.25	3.12	>100	**2m**	0.78	3.12	>100
**1n**	12.5	12.5	>100	**2n**	1.56	1.56	>100
**1o**	25	50	>100	**2o**	>100	>100	>100
**1p**	12.5	3.12	>100	**2p**	6.25	12.5	>100
**1q**	50	100	>100	**2q**	0.78	3.12	>100
**1r**	6.25	3.12	>100	**2r**	1.56	6.25	>100
**1s**	25	12.5	>100	**2s**	3.12	3.12	>100
**LEV**	0.05	0.20	0.02	**3a**	>100	>100	>100

Levofloxacin (LEV) was used as a positive control.

## Supplementary Materials

The supplementary materials are available online.

## References

[1] Sun X., Hong Z., Liu M., Guo S., Yang D., Wang Y., Lan T., Gao L., Qi H., Gong P. (2017). Design, synthesis, and biological activity of novel tetrahydropyrazolopyridone derivatives as FXa inhibitors with potent anticoagulant activity. Bioorg. Med. Chem..

[2] Chapleo C.B., Myers M., Myers P.L., Saville J.F., Smith A.C., Stillings M.R., Tulloch I.F., Walter D.S., Welbourn A.P. (1986). The oxidation of 1-thioaroylsemicarbazides. J. Med. Chem..

[3] Luszczki J.J., Karpińska M., Matysiak J., Niewiadomy A. (2015). Characterization and preliminary anticonvulsant assessment of some 1,3,4-thiadiazole derivatives. Pharmacol. Rep..

[4] Holla B.S., Gonsalves R., Shenoy S. (2000). Synthesis and antibacterial studies of a new series of 1, 2-bis (1, 3, 4-oxadiazol-2-yl) ethanes and 1,2-*bis* (4-amino-1,2,4-triazol-3-yl) ethanes. Eur. J. Med. Chem..

[5] Cesur N., Birteksöz S., Ötük G. (2002). Synthesis and biological evaluation of some new thiosemicarbazide, 4-thiazolidinone, 1,3,4-oxadiazole and 1,2,4-triazole-3-thione derivatives bearing imidazo [1, 2-a] pyridine moiety. Acta Pharm. Sci..

[6] Laddi U., Desai S., Bennur R., Bennur S. (2002). Some new 1,3,4-oxadiazoles as antimicrobial agents. Indian J. Heterocycl. Chem..

[7] Rahman M.A. (2014). ZnX2 (X = Cl, Br) catalyzed efficient regioselective synthesis of 1,3,4-oxadiazole and 1,3,4-thiadiazole rings and their antibacterial studies. Ph.D. Thesis.

[8] Mishra P., Rajak H., Mehta A. (2005). Synthesis of Schiff bases of 2-amino-5-aryl-1,3,4-oxadiazoles and their evaluation for antimicrobial activities. J. Gen. Appl. Microbiol..

[9] Stephens C.E., Tanious F., Kim S., Wilson W.D., Schell W.A., Perfect J.R., Franzblau S.G., Boykin D.W. (2001). Diguanidino and “reversed” diamidino 2,5-diarylfurans as antimicrobial agents. J. Med. Chem..

[10] Zou X.J., Lai L.H., Jin G.Y., Zhang Z.X. (2002). Synthesis, fungicidal activity, and 3D-QSAR of pyridazinone-substituted 1,3,4-oxadiazoles and 1,3,4-thiadiazoles. J. Agric. Food Chem..

[11] Bondock S., Adel S., Etman H.A., Badria F.A. (2012). Synthesis and antitumor evaluation of some new 1,3,4-oxadiazole-based heterocycles. Eur. J. Med. Chem..

[12] Dawood K.M., Gomha S.M. (2015). Synthesis and Anti-cancer Activity of 1,3,4-Thiadiazole and 1,3-Thiazole Derivatives Having 1,3,4-Oxadiazole Moiety. J. Heterocycl. Chem..

[13] Durgashivaprasad E., Mathew G., Sebastian S., Reddy S.M., Mudgal J., Nampurath G.K. (2014). Novel 2,5-disubstituted-1,3,4-oxadiazoles as anti-inflammatory drugs. Indian J. Pharmacol..

[14] Singh A.K., Lohani M., Parthsarthy R. (2013). Synthesis, characterization and anti-inflammatory activity of some 1, 3, 4-oxadiazole derivatives. Iran. J. Pharm. Res..

[15] Mullican M.D., Wilson M.W., Conner D.T., Kostlan C.R., Schrier D.J., Dyer R.D. (1993). Design of 5-(3,5-di-tert-butyl-4-hydroxyphenyl)-1,3,4-thiadiazoles-1,3,4-oxadiazoles, and-1,2,4-triazoles as orally active, nonulcerogenic antiinflammatory agents. J. Med. Chem..

[16] Zheng X., Li Z., Wang Y., Chen W., Huang Q., Liu C., Song G. (2003). Syntheses and insecticidal activities of novel 2,5-disubstituted 1,3,4-oxadiazoles. J. Fluorine Chem..

[17] Idoux J.P., Gibbs-Rein K.S., Gupton J.T., Cunningham G.N. (1988). Synthesis and insecticidal activity of some 2, 5-(fluoroalkoxyphenyl)-1,3,4-oxadiazoles and their *N*,*N*′-dibenzoylhydrazine precursors. J. Chem. Eng. Data.

[18] Tyagi M., Kumar A. (2002). Synthesis of 2-[2’-carbonyl-5’-(heteroarylinomethylene)-1’,3’,4’-thiadiazol-2’-yl/oxadiazole-2’-yl)]-4,5-dihydroimidazolines as hypotensive agents. Oriental J. Chem..

[19] Almasirad A., Tabatabai S.A., Faizi M., Kebriaeezadeh A., Mehrabi N., Dalvandi A., Shafiee A. (2004). Synthesis and anticonvulsant activity of new 2-substituted-5-[2-(2-fluorophenoxy) phenyl]-1,3,4-oxadiazoles and 1,2, 4-triazoles. Bioorg. Med.Chem. Lett..

[20] Ekins S., Freundlich J.S., Hobrath J.V., White E.L., Reynolds R.C. (2014). Combining computational methods for hit to lead optimization in Mycobacterium tuberculosis drug discovery. Pharm. Res..

[21] Mochona B., Qi X., Euynni S., Sikazwi D., Mateeva N., Soliman K.F. (2016). Design and evaluation of novel oxadiazole derivatives as potential prostate cancer agents. Bioorg. Med.Chem. Lett..

[22] Soares de Oliveira C., Lira B.F., Barbosa-Filho J.M., Lorenzo J.G.F., Filgueiras de Athayde-Filho P. (2012). Synthetic Approaches and Pharmacological Activity of 1,3,4-Oxadiazoles: A Review of the Literature from 2000–2012. Molecules.

[23] Rivera N.R., Balsells J., Hansen K.B. (2006). Synthesis of 2-amino-5-substituted-1,3,4-oxadiazoles using 1,3-dibromo-5,5-dimethylhydantoin as oxidant. Tetrahedron Lett..

[24] Dolman S.J., Gosselin F., O’Shea P.D., Davies I.W. (2006). Superior reactivity of thiosemicarbazides in the synthesis of 2-amino-1,3,4-oxadiazoles. J. Org. Chem..

[25] Matsuno K., Masuda Y., Uehara Y., Sato H., Muroya A., Takahashi O., Yokotagawa T., Furuya T., Okawara T., Otsuka M. (2010). Identification of a New Series of STAT3 Inhibitors by Virtual Screening. ACS Med. Chem. Lett..

[26] Fülöp F., Semega E., Dombi C., Bernath G. (1990). Synthesis of new heterocyclic compounds as potential pharmaceutical agents. J. Heterocycl. Chem..

[27] Küçükgüzel G., Kocatepe A., De Clercq E., Şahin F., Güllüce M. (2006). Synthesis and biological activity of 4-thiazolidinones, thiosemicarbazides derived from diflunisal hydrazide. Eur. J. Med. Chem..

[28] Coppo F.T., Evans K.A., Graybill T.L., Burton G. (2004). Efficient one-pot preparation of 5-substituted-2-amino-1, 3, 4-oxadiazoles using resin-bound reagents. Tetrahedron Lett..

[29] Yang S.-J., Choe J.-H., Abdildinova A., Gong Y.-D. (2015). A highly efficient diversification of 2-amino/amido-1,3,4-oxadiazole and 1,3,4-thiadiazole derivatives via reagent-based cyclization of thiosemicarbazide intermediate on solid-phase. ACS Comb. Sci..

[30] Omar A., Mohsen M., Aboulwafa O.M. (1984). Synthesis and anticonvulsant properties of a novel series of 2-substituted amino-5-aryl-1,3,4-oxadiazole derivatives. J. Heterocycl. Chem..

[31] Kurzer F., Doyle K.M. (1986). The oxidation of 1-thioaroylsemicarbazides. J. Chem. Soc. Perk. Trans. 1.

[32] Zou X., Zhang Z., Jin G. (2002). Synthesis and biological activity of 1,3,4-oxadiazole-substituted pyridazinones. J. Chem. Res..

[33] Shinde V.N., Ugarkar B.G., Ghorpade S.R. (2013). A convenient synthesis of 5-substituted 2-amino-1,3,4-oxadiazoles from corresponding acylthiosemicarbazides using iodine and Oxone. J. Chem. Res..

[34] Vachal P., Toth L.M. (2004). General facile synthesis of 2,5-diarylheteropentalenes. Tetrahedron Lett..

[35] Chaudhari P.S., Pathare S.P., Akamanchi K.G. (2012). *o*-Iodoxybenzoic Acid Mediated Oxidative Desulfurization Initiated Domino Reactions for Synthesis of Azoles. J. Org. Chem..

[36] Niu P., Kang J., Tian X., Song L., Liu H., Wu J., Yu W., Chang J. (2014). Synthesis of 2-amino-1,3,4-oxadiazoles and 2-amino-1,3,4-thiadiazoles via sequential condensation and I_2_-mediated oxidative C-O/C-S bond formation. J. Org. Chem..

[37] Holla B.S., Prasanna C.S., Poojary B., Rao K.S., Shridhara K., Bhat U.G. (2004). Synthesis and characterization of 1,3,4-thiadiazole and 1,3,4-oxadiazole derivatives containing 2-chloropyridin-5-yl-methyl moiety. Indian J. Chem. Sect. B Org. Chem. Incl. Med. Chem..

[38] Xia Q., He Q., Xu D., Sun D., Peng Z. (2010). Synthesis, characterization and antibacterial activity of five-membered heterocycles. Huaxue Xuebao.

[39] Rostom S.A.F., Shalaby M.A., El-Demellawy M.A. (2003). Polysubstituted pyrazoles, part 5. Synthesis of new 1-(4-chlorophenyl)-4-hydroxy-1H-pyrazole-3-carboxylic acid hydrazide analogs and some derived ring systems. A novel class of potential antitumor and anti-HCV agents. Eur. J. Med. Chem..

[40] Yale H.L., Losee K. (1966). 2-Amino-5-substituted 1,3,4-oxadiazoles and 5-imino-2-substituted Δ2-1,3,4-oxadiazolines. A group of novel muscle relaxants. J. Med. Chem..

[41] Maghari S., Ramezanpour S., Darvish F., Balalaie S., Rominger F., Bijanzadeh H.R. (2013). A new and efficient synthesis of 1,3,4-oxadiazole derivatives using TBTU. Tetrahedron.

[42] Singh C.B., Ghosh H., Murru S., Patel B.K. (2008). Hypervalent iodine(III)-mediated regioselective *N*-acylation of 1,3-disubstituted thioureas. J. Org. Chem..

[43] Ghosh H., Yella R., Nath J., Patel B.K. (2008). Desulfurization mediated by hypervalent iodine(III): A novel strategy for the construction of heterocycles. Eur. J. Org. Chem..

[44] Ameryckx A., Thabault L., Pochet L., Leimanis S., Poupaert J.H., Wouters J., Joris B., Van Bambeke F., Frederick R. (2018). 1-(2-Hydroxybenzoyl)-thiosemicarbazides are promising antimicrobial agents targeting D-alanine-d-alanine ligase in bacterio. Eur. J. Med. Chem..

[45] Azhari S.J., Mlahi M.R., Al-Asmy A.A., Mostafa M.M. (2015). Synthesis of novel binary and ternary complexes derived from 1-(2-hydroxy benzoyl)-4-phenylthiosemicarbazide (L1) and 2,2′-dipyridyl (L2) with CoII, CuII and ZnII salts. Spectrochim. Acta Part. A.

[46] Prajapat P. (2017). Utility of drug discovery in medicinal and organic chemistry. Mod. Chem. Appl..

[47] Nielsen T.E., Schreiber S.L. (2008). Towards the optimal screening collection. A synthesis strategy. Angew. Chem. Int. Ed..

[48] Schreiber S.L. (2011). Organic synthesis toward small-molecule probes and drugs. Proc. Natl. Acad. Sci. USA.

[49] Barbachyn M.R., Ford C.W. (2003). Oxazolidinone structure-activity relationships leading to linezolid. Angew. Chem. Int. Ed..

[50] Schillaci D., Spano V., Parrino B., Carbone A., Montalbano A., Barraja P., Diana P., Cirrincione G., Cascioferro S. (2017). Pharmaceutical Approaches to Target Antibiotic Resistance Mechanisms. J. Med. Chem..

[51] Wu S., Xia J., Wen G., Feng B., Jia Y.-Q., Zhang W.-X., Yang Q.-Y., Zhang C., Qi Y. (2018). Preparation method of 1-benzoyl-4-acyl semicarbazide derivatives as antibacterial drug.

[52] Andrews J. M. (2001). Determination of minimum inhibitory concentrations. J. Antimicrobial. Chemotherapy.

[53] Gao X., Lu Y., Fang L., Fang X., Xing Y., Gou S., Xi T. (2013). Synthesis and anticancer activity of some novel 2-phenazinamine derivatives. Eur. J. Med. Chem..

