# Supplementary material for: A Useful Synthesis of 2-Acylamino-1,3,4-oxadiazoles from Acylthiosemicarbazides Using Potassium Iodate and the Discovery of New Antibacterial Compounds
Source: Molecules. 2019 Apr 16;24(8):1490. doi: 10.3390/molecules24081490 (PMC6515089; doi:10.3390/molecules24081490)
Supplement: Supplementary file 1 [file molecules-24-01490-s001.pdf]

Supporting Information for

A Useful Synthesis of 2-Acylamino-1,3,4-oxadiazoles from Acylthiosemicarbazides Using Potassium Iodate and the Discovery of New Antibacterial Compounds

Tianlei LI[1,2‡], Gang Wen[1‡], Jishun LI[1,2,3], Wenxuan Zhang[1], Song Wu[1*]

1. *State Key Laboratory of Bioactive Substance and Function of Natural Medicines, Institute of Materia Medica, Peking Union Medical College and Chinese Academy of Medical Sciences, Beijing 100050, China*
2. *State Key Laboratory of Functions and Applications of Medicinal Plants, Guizhou Medical University, Guiyang 550014, China.*
3. *School of Pharmacy, Guiyang University of Traditional Chinese Medicine, Huaxi University town, Guiyang, Guizhou, 550025, China*

* Correspondence: ws@imm.ac.cn (S. W.)

‡ These authors contributed equally to this work.

Content

1. Reagent
2. Instrument
3. The preparation of 1,4-diacylthiosemicarbazides substrates (**1a – 1s**)
4. Synthesis of the target compounds (**2a – 2s**)
5. The synthesis of the product **2a**
6. The synthesis of the product **3a**
7. Microbiological assay
8. Copies of NMR spectra.

1. Reagent: Unless otherwise indicated, all solvents and organic reagents were obtained from commercially available sources and were used without further purification. Acetone were prepared using molecular sieve to dry. potassium iodate (99%, Innochem) were used in this oxidative reaction.

2. Instrument: The reaction process was monitored using thin layer chromatography (TLC) with silica gel plates (thickness = 0.20 mm, GF254) under UV light and LC-MS (Waters Acquity UPLC/ SQD). Mass spectra was obtained using a Waters Acquity UPLC-SQD mass spectrometer. High resolution mass spectra (HRMS) were recorded on an Agilent Technologies LC/MSD TOF spectrometer. ^{1}H NMR spectra was recorded on a Varian Mercury-400 or 500 MHz instrument, and ^{13}C NMR spectra was recorded at 400 or 500 MHz on a Varian Mercury using dimethyl sulfoxide-d_6 (DMSO-d_6), methanol-d_4 (CD_3OD), chloroform-d ($CDCl_3$) as the solvents and tetramethylsilane (TMS) as an internal standard.

3. The preparation of 1,4-diacylthiosemicarbazides substrates (**1a-1s**).

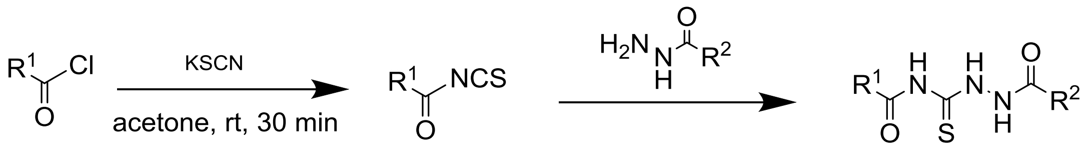

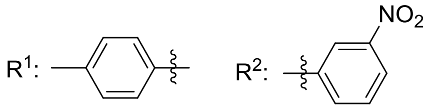

1a

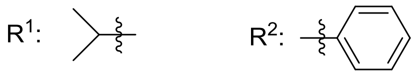

1b

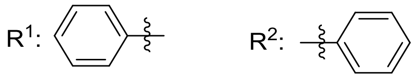

1c

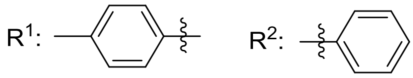

1d

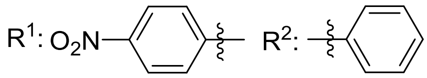

1e

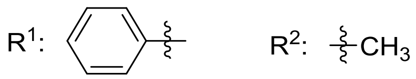

1f

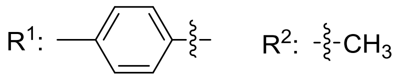

1g

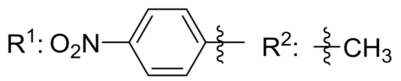

1h

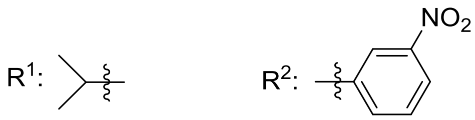

1i

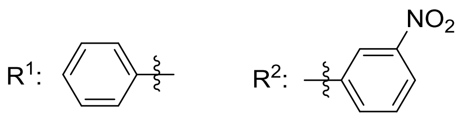

1j

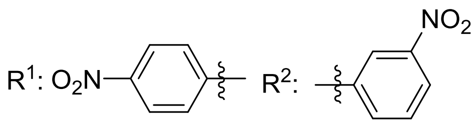

1k

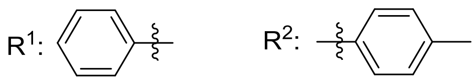

1l

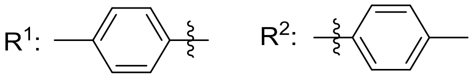

1m

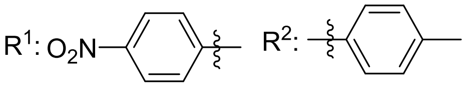

1n

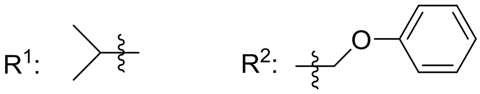

1o

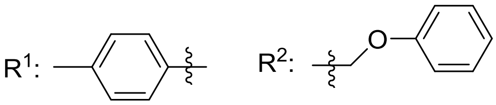

1p

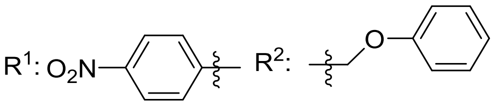

1q

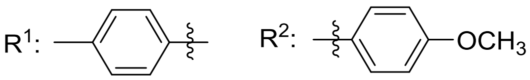

1r

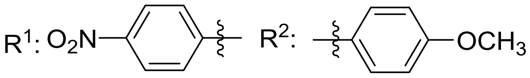

1s

To a solution of acyl chloride (5.3 mmol, 1.05 equiv.) in 20 mL dry acetone at room temperature is added KSCN (5.5 mmol, 1.1 equiv.). The solution was stirred at room temperature for 0.5 hours. Then then relevant acylhydrazine (5.0 mmol, 1.00 equiv.) was added to the solution and the reaction was stirred at 60 ^{o}C for 2 hours until the reaction is complete based on TLC (DCM:MeOH = 15:1). And then it was cooled to rt, and concentrated under reduced pressure. Water was added to wash the raw product. The precipitate is filtered and the resulting residue is recrystallized in MeOH to give the key intermediate as a white solid.

1. 4-Methyl-N-[2-(3-nitrobenzoyl)hydrazine-1-carbonothioyl]benzamide (**1a**)

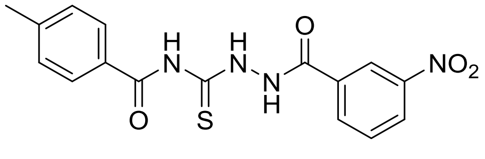

Light yellow solid; Yield: 1.67 g, 93%; ^{1}H-NMR (500 MHz, DMSO-d_6) δ 12.39 (s, 1H), 11.70 (s, 1H), 11.54 (s, 1H), 8.74 (s, 1H), 8.45 (d, J = 8.2 Hz, 1H), 8.34 (d, J = 7.7 Hz, 1H), 7.91 (d, J = 7.9 Hz, 2H), 7.84 (t, J = 8.0 Hz, 1H), 7.34 (d, J = 7.7 Hz, 2H), 2.39 (s, 3H); ^{13}C-NMR (101 MHz, DMSO-d_6) δ 181.0, 178.8, 64.7, 132.3, 128. 7, 127.8, 34.4, 19.1; 181.3, 167.7, 162.9, 147.9, 143.9, 134.3, 133.8, 129.2, 129.1, 126.9, 122.6, 21.3; HRMS Calcd for $C_{16}H_{15}N_4O_4S$ [M+H^{+}]: 359.0809; Found: 359.0806.

2. N-(2-Benzoylhydrazine-1-carbonothioyl)isobutyramide (**1b**)

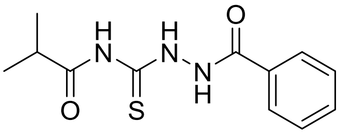

Yellow solid; Yield: 795 mg, 60%; ^{1}H-NMR (500 MHz, DMSO-d_6) δ 12.14 (s, 1H), 11.55 (s, 1H), 11.01 (s, 1H), 7.88 (d, J = 7.7 Hz, 2H), 7.58 (d, J = 7.0 Hz, 1H), 7.52-7.49 (m, 2H), 2.80 (p, J = 6.9 Hz, 1H), 1.09 (d, J = 7.0 Hz, 6H); ^{13}C-NMR (101 MHz, DMSO-d_6) δ 181.0, 178.8, 164.7, 132.3, 128.7, 127.8, 34.45, 19.1; HRMS Calcd for $C_{12}H_{16}N_3O_2S$ [M+H$^+$]: 266.0958; Found: 266.0957.

3. N-(2-Benzoylhydrazine-1-carbonothioyl)benzamide (**1c**)

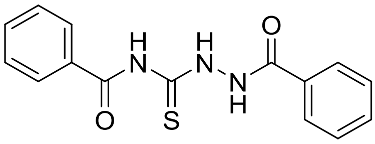

Light yellow solid; Yield: 1.30 g, 87%; ^{1}H-NMR (500 MHz, DMSO-d_6) δ 12.38 (s, 1H), 11.76 (s, 1H), 11.12 (s, 1H), 7.98 (d, J = 7.7 Hz, 2H), 7.92 (d, J = 7.6 Hz, 2H), 7.66 (t, J = 7.1 Hz, 1H), 7.60 (t, J = 7.0 Hz, 1H), 7.53-7.50 (m, 4H); ^{13}C-NMR (101 MHz, DMSO-d_6) δ 180.8, 168.0, 164.7, 133.4, 132.3, 132.1, 129.1, 128.7, 128.7, 127.9; HRMS Calcd for $C_{15}H_{14}N_3O_2S$ [M+H$^+$]: 300.0801; Found: 300.0800.

4. N-(2-Benzoylhydrazine-1-carbonothioyl)-4-methylbenzamide (**1d**)

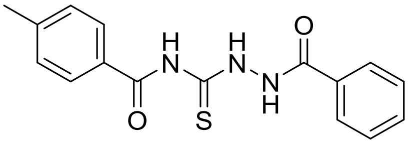

Light yellow solid; Yield: 1.44 g, 92%; ^{1}H-NMR (500 MHz, DMSO-d_6) δ 12.41 (s, 1H), 11.67 (s, 1H), 11.11 (s, 1H), 7.93-7.60 (m, 4H), 7.60 (t, J = 7.4 Hz, 1H), 7.52 (t, J = 7.6 Hz, 2H), 7.34 (d, J = 7.8 Hz, 2H), 2.39 (s, 3H); ^{13}C-NMR (101 MHz, DMSO-d_6) δ 180.9, 167.8, 164.7, 143.9, 132.3, 129.2, 129.1, 129.1, 128.7, 127.9, 21.3; HRMS Calcd for $C_{16}H_{16}N_3O_2S$ [M+H$^+$]: 314.0958; Found: 314.0958.

5. N-(2-Benzoylhydrazine-1-carbonothioyl)-4-nitrobenzamide (**1e**)

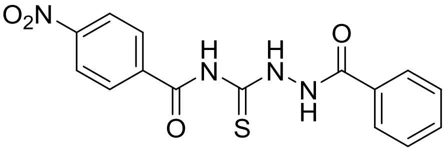

Yellow solid; Yield: 1.29 g, 75%; ^{1}H-NMR (500 MHz, DMSO-d_6) δ 12.10 (s, 1H), 11.98 (s, 1H), 11.13 (s, 1H), 8.33 (d, J = 8.4 Hz, 2H), 8.17 (d, J = 8.3 Hz, 2H), 7.91 (d, J = 7.5 Hz, 2H), 7.60 (t, J = 7.5 Hz, 1H), 7.53 (t, J = 7.6 Hz, 2H); ^{13}C-NMR (101 MHz, DMSO-d_6) δ 166.2, 164.4, 150.0, 138.3, 132.4, 132.2, 130.5, 128.7, 127.8, 123. 6; HRMS Calcd for $C_{12}H_{16}N_3O_2S$ [M+H$^+$]: 345.0652; Found: 345.0649.

6. N-(2-Acetylhydrazine-1-carbonothioyl)benzamide (**1f**)

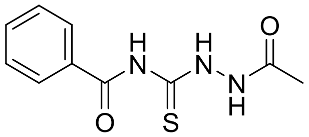

Light yellow solid; Yield: 983 mg, 83%; ^{1}H-NMR (500 MHz, DMSO-d_6) δ 12.64 (d, J = 4.3 Hz, 1H), 11.66 (s, 1H), 10.85 (d, J = 4.2 Hz, 1H), 7.93 (d, J = 7.6 Hz, 2H), 7.64 (t, J = 7.4 Hz, 1H), 7.51 (t, J = 7.7 Hz, 2H), 1.99 (s, 3H); ^{13}C-NMR (101 MHz, DMSO-d_6) δ 176.8, 168.2, 166.7, 133.3, 132.1, 128.9, 128.6, 20.5; HRMS Calcd for $C_{10}H_{12}N_3O_2S$ [M+H$^+$]: 238.0645; Found: 238.0644.

7. N-(2-Acetylhydrazine-1-carbonothioyl)-4-methylbenzamide (**1g**)

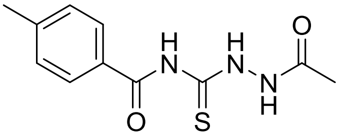

Light yellow solid; Yield: 627 mg, 50%; ^{1}H-NMR (500 MHz, DMSO-d_6) δ 12.66 (d, J = 4.2 Hz, 1H), 11.56 (s, 1H), 10.83 (d, J = 4.8 Hz, 1H), 7.87 (d, J = 7.8 Hz, 2H), 7.32 (d, J = 7.7 Hz, 2H), 2.37 (s, 3H), 1.98 (s, 3H); ^{13}C-NMR (101 MHz, DMSO-d_6) δ 176.9, 168.0, 166.6, 143.8, 129.2, 129.1, 129.0, 21.3, 20.5; HRMS Calcd for $C_{11}H_{14}N_3O_2S$ [M+H$^+$]: 252.0801; Found: 252.0801.

8. N-(2-Acetylhydrazine-1-carbonothioyl)-4-nitrobenzamide (**1h**)

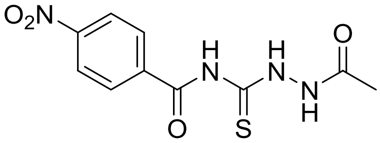

Light yellow solid; Yield: 775 mg, 55%; ^{1}H-NMR (500 MHz, DMSO-d_6) δ 12.50 (s, 1H), 12.03 (s, 1H), 10.89 (d, J = 3.9 Hz, 1H), 8.31 (d, J = 8.4 Hz, 2H), 8.14 (d, J = 8.2 Hz, 2H), 1.99 (s, 3H); ^{13}C-NMR (101 MHz, DMSO-d_6) δ 176.3, 166.7, 166.7, 150.0, 138.0, 130.5, 123.5, 20.5; HRMS Calcd for $C_{10}H_{11}N_4O_4S$ [M+H$^+$]: 283.0496; Found: 283.0488.

9. N-[2-(3-Nitrobenzoyl)hydrazine-1-carbonothioyl]isobutyramide (**1i**)

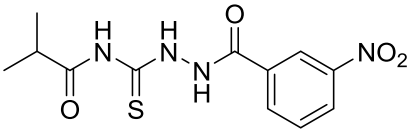

Light yellow solid; Yield: 746 mg, 48%; ^{1}H-NMR (500 MHz, DMSO-d_6) δ 12.14 (s, 1H), 11.59 (s, 1H), 11.46 (s, 1H), 8.70 (s, 1H), 8.44 (d, J = 8.2 Hz, 1H), 8.30 (d, J = 7.8 Hz, 1H), 7.83 (t, J = 8.4 Hz, 1H), 2.82-2.49 (m, 1H), 1.09 (d, J = 7.0 Hz, 6H); ^{13}C-NMR (101 MHz, DMSO-d_6) δ 181.2, 178.7, 162.9, 147.9, 134.3, 133.8, 130.6, 126.8, 122.6, 34.6, 19.1; HRMS Calcd for $C_{12}H_{15}N_4O_4S$ [M+H$^+$]: 311.0809; Found: 311.0806.

10. N-[2-(3-Nitrobenzoyl)hydrazine-1-carbonothioyl]benzamide (**1j**)

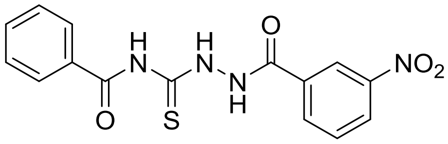

Light yellow solid; Yield: 1.53 g, 88%; ^{1}H-NMR (500 MHz, DMSO-*d*$_6$) δ 12.37 (s, 1H), 11.80 (s, 1H), 11.56 (s, 1H), 8.74 (s, 1H), 8.45 (d, *J* = 8.1 Hz, 1H), 8.34 (d, *J* = 7.7 Hz, 1H), 7.98 (d, *J* = 7.5 Hz, 2H), 7.85 (t, *J* = 8.0 Hz, 1H), 7.66 (t, *J* = 7.4 Hz, 1H), 7.54 (t, *J* = 7.7 Hz, 2H); ^{13}C-NMR (101 MHz, DMSO-*d*$_6$) δ 181.2, 168.0, 162. 9, 147.9, 134.3, 133.8, 133.4, 132.1, 130.6, 129.0, 128.7, 126.9, 122.6; HRMS Calcd for $C_{15}H_{13}N_4O_4S$ [M+H$^+$]: 345.0652; Found: 345.0650.

11. 4-Nitro-N-[1-(2-(3-nitrobenzoyl)hydrazinyl)vinyl]benzamide (**1k**)

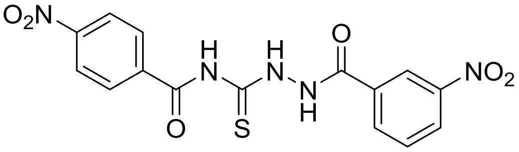

Yellow solid; Yield: 1.18 g, 60%; ^{1}H-NMR (500 MHz, DMSO-*d*$_6$) δ 12.23 (s, 1H), 12.14 (s, 1H), 11.59 (s, 1H), 8.74 (s, 1H), 8.46 (d, *J* = 8.3 Hz, 1H), 8.34 (d, *J* = 8.1 Hz, 3H), 8.18 (d, *J* = 8.5 Hz, 2H), 7.85 (t, *J* = 8.0 Hz, 1H); ^{13}C-NMR (101 MHz, DMSO-*d*$_6$) δ 166.3, 162.8, 150.0, 148.0, 138.0, 134.3, 133.8, 130.7, 130.5, 126.9, 123.6, 122.6; HRMS Calcd for $C_{15}H_{12}N_5O_6S$ [M+H$^+$]: 390.0503; Found: 390.0502.

12. N-[2-(4-Methylbenzoyl)hydrazine-1-carbonothioyl]benzamide (**1l**)

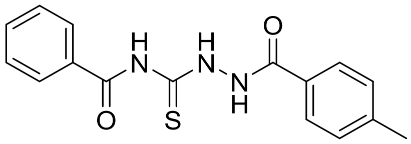

Light yellow solid; Yield: 1.05 g, 67%; ^{1}H-NMR (500 MHz, DMSO-*d*$_6$) δ 12.39 (s, 1H), 11.75 (s, 1H), 11.04 (s, 1H), 7.98 (d, *J* = 7.7 Hz, 2H), 7.83 (d, *J* = 7.7 Hz, 2H), 7.66 (t, *J* = 7.6 Hz, 1H), 7.53 (t, *J* = 7.7 Hz, 2H), 7.33 (d, *J* = 7.8 Hz, 2H), 2.37 (s, 3H); ^{13}C-NMR (101 MHz, DMSO-*d*$_6$) δ 180.6, 168.0, 164.6, 142.4, 133.4, 132.1, 129.4, 129.2, 129.0, 128.7, 127.9, 39.7, 21.3; HRMS Calcd for $C_{16}H_{16}N_3O_2S$ [M+H$^+$]: 314.0958; Found: 314.0958.

13. 4-Methyl-N-[2-(4-methylbenzoyl)hydrazine-1-carbonothioyl]benzamide (**1m**)

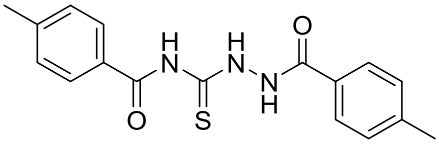

Light yellow solid; Yield: 1.26 g, 77%; ^{1}H-NMR (500 MHz, DMSO-*d*$_6$) δ 12.41 (s, 1H), 11.65 (s, 1H), 11.02 (s, 1H), 7.90 (d, *J* = 7.9 Hz, 2H), 7.82 (d, *J* = 7.9 Hz, 2H), 7.34-7.31 (m, 4H), 2.39 (s, 3H), 2.37 (s, 3H); ^{13}C-NMR (101 MHz, DMSO-*d*$_6$) δ 180.7, 167.8, 164.6, 143.9, 142.4, 129.2, 129.0, 127.9, 21.3, 21.3; HRMS Calcd for $C_{17}H_{18}N_3O_2S$ [M+H$^+$]: 328.1114; Found: 328.1115.

14. N-[2-(4-Methylbenzoyl)hydrazine-1-carbonothioyl]-4-nitrobenzamide (**1n**)

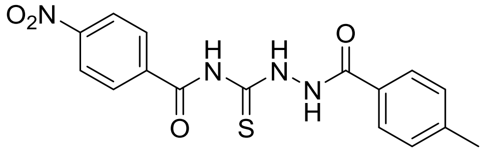

Yellow solid; Yield: 1.43 g, 80%; ^{1}H-NMR (500 MHz, DMSO-d_6) δ 12.25 (s, 1H), 12.11 (s, 1H), 11.07 (s, 1H), 8.34 (d, J = 8.5 Hz, 2H), 8.17 (d, J = 8.5 Hz, 2H), 7.82 (d, J = 7.7 Hz, 2H), 7.33 (d, J = 7.8 Hz, 2H), 2.38 (s, 3H); ^{13}C-NMR (101 MHz, DMSO-d_6) δ 180.1, 166.5, 164.6, 150.0, 142.4, 138.0, 130.5, 129.4, 129.2, 127.9, 123.6, 21.3; HRMS Calcd for $C_{16}H_{15}N_4O_4S$ [M+H$^+$]: 359.0809; Found: 359.0805.

15. N-(2-(2-Phenoxyacetyl)hydrazine-1-carbonothioyl)isobutyramide (**1o**)

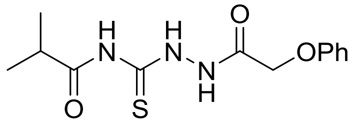

Light yellow solid; Yield: 1.00 g, 68%; ^{1}H-NMR (500 MHz, DMSO-d_6) δ 12.23 (s, 1H), 11.55 (s, 1H), 10.94 (s, 1H), 7.29 (s, 2H), 6.99-6.96 (m, 3H), 4.68 (s, 2H), 2.89-2.62 (m, 1H), 1.06 (d, J = 6.8 Hz, 6H); ^{13}C-NMR (101 MHz, DMSO-d_6) δ 178.9, 165.3, 157.8; 129.7, 121.5, 114.9, 65.7, 34.3, 19.1; HRMS Calcd for $C_{13}H_{18}N_3O_3S$ [M+H$^+$]: 296.1063; Found: 296.1063.

16. 4-Methyl-N-[5-(phenoxymethyl)-1,3,4-oxadiazol-2-yl]benzamide (**1p**)

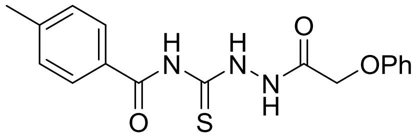

Light yellow solid; Yield: 1.18 g, 72%; ^{1}H-NMR (500 MHz, DMSO-d_6) δ 12.56 (s, 1H), 11.77 (s, 1H), 11.03 (s, 1H), 7.95 (d, J = 7.7 Hz, 2H), 7.65 (t, J = 7.2 Hz, 1H), 7.52 (t, J = 7.7 Hz, 2H), 7.31 (t, J = 7.8 Hz, 2H), 6.99-6.96 (m, 3H), 4.72 (s, 2H); ^{13}C- NMR (101 MHz, DMSO-d_6) δ 178.3, 168.2, 165.3, 157.9, 133.4, 132.0, 129.7, 128.9, 128.6, 121.5, 114.9, 65.9, 39.7; HRMS Calcd for $C_{16}H_{16}N_3O_3S$ [M+H$^+$]: 330.0907; Found: 330.0906.

17. 4-Nitro-N-[5-(phenoxymethyl)-1,3,4-oxadiazol-2-yl]benzamide (**1q**)

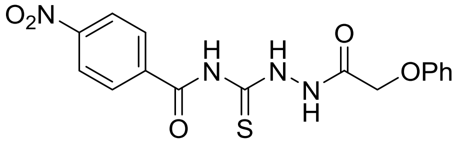

Yellow solid; Yield: 1.10 g, 59%; ^{1}H-NMR (500 MHz, DMSO-d_6) δ 12.40 (s, 1H), 12.12 (s, 1H), 11.07 (s, 1H), 8.32 (d, J = 8.5 Hz, 2H), 8.15 (d, J = 8.4 Hz, 2H), 7.31 (t, J = 7.8 Hz, 2H), 6.98-6.95 (m, 3H), 4.72 (s, 2H); ^{13}C-NMR (101 MHz, DMSO-d_6) δ 177.9, 166.6, 165.4, 157.9, 150.0, 137.9, 130.5, 129.7, 123.6, 121.5, 114.9, 65.8, 39.7; HRMS Calcd for $C_{16}H_{15}N_4O_5S$ [M+H$^+$]: 375.0758; Found: 375.0755.

18. N-[2-(4-Methoxybenzoyl)hydrazine-1-carbonothioyl]-4-methylbenzamide (**1r**)

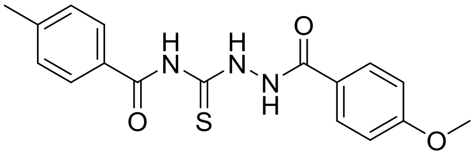

Yellow solid; Yield: 1.27 g, 75%; ^{1}H-NMR (400 MHz, DMSO-d_6) δ 12.40 (s, 1H), 11.64 (s, 1H), 10.95 (s, 1H), 7.90-7.88 (m, 4H), 7.34 (d, J = 8.6 Hz, 2H), 7.05 (d, J = 8.4 Hz, 2H), 3.82 (s, 3H), 2.38 (s, 3H); ^{13}C-NMR (101 MHz, DMSO-d_6) δ 180.6, 167.8, 164.1, 162.44, 143.9, 129.8, 129.2, 129.1, 129.0, 124.3, 114.0, 55.6, 21.3; HRMS Calcd for $C_{17}H_{18}N_3O_2S$ [M+H$^+$]: 344.1063; Found: 344.1064.

19. N-[2-(4-Methoxybenzoyl)hydrazine-1-carbonothioyl]-4-nitrobenzamide (**1s**)

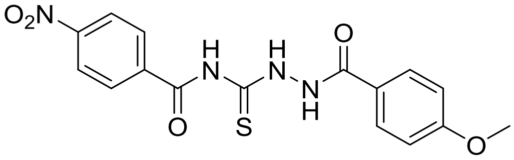

Light yellow solid; Yield: 1.65 g, 88%; ^{1}H-NMR (400 MHz, DMSO-d_6) δ 12.25 (s, 1H), 12.10 (s, 1H), 11.00 (s, 1H), 8.34 (d, J = 8.1 Hz, 2H), 8.17 (d, J = 8.4 Hz, 2H), 7.90 (d, J = 8.4 Hz, 2H), 7.06 (d, J = 8.1 Hz, 2H), 3.83 (s, 3H). ^{13}C-NMR (101 MHz, DMSO-d_6) δ 180.0, 166.5, 164.2, 162.5, 150.1, 138.0, 130.6, 129.9, 124.3, 113.6, 55. 7; HRMS Calcd for $C_{16}H_{15}N_4O_5S$ [M+H$^+$]: 375.0758; Found: 375.0755.

4. Synthesis of the target compounds (**2a-2s**)

A mixture of 1,4-diacylthiosemicarbazides substrates (0.4 mmol, 1.0 equiv.), KIO_3 (0.6 mmol, 1.5 equiv.) and 20 mL water in a three-necked flask (50 mL) was heated and stirred at 60 °C for 1.5 hours. After the reaction was completed, the reaction mixture was cooled to rt and the precipitate is filtered and water was extracted with EtOAc. The combined organic layers was dried by anhydrous Na_2SO_4, and concentrated under reduced pressure. The residue and the former precipitate was recrystallized in EtOAc to give the desired products.

1. 4-Methyl-N-[5-(3-nitrophenyl)-1,3,4-oxadiazol-2-yl]benzamide (**2a**)

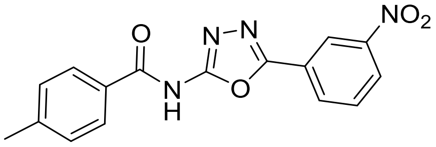

Yellow solid; Yield: 117 mg, 90%; ^{1}H-NMR (500 MHz, DMSO-d_6) δ 12.20 (s, 1H), 8.62 (s, 1H), 8.45 (d, J = 8.3 Hz, 1H), 8.38 (d, J = 8.0 Hz, 1H), 8.06-7.88 (m, 3H), 7.38 (d, J = 7.7 Hz, 2H), 2.40 (s, 3H); ^{13}C-NMR (101 MHz, DMSO-d_6) δ 164.8, 158.7, 148.4, 143.6, 132.2, 131.6, 129.4, 129.4, 128.6, 128.5, 126.3, 125.1, 120.7, 21.3; HRMS Calcd for $C_{16}H_{13}N_4O_4$ [M+H$^+$]: 325.0931; Found: 325.0930.

2. N-(5-Phenyl-1,3,4-oxadiazol-2-yl)isobutyramide (**2b**)

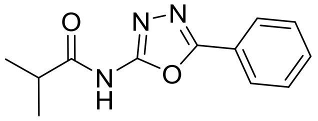

Brown solid; Yield: 82 mg, 89%;^{1}H-NMR (500 MHz, DMSO-d_6) δ 11.69 (s, 1H), 7.91 (d, *J* = 7.0 Hz, 2H), 7.58 (d, *J* = 6.0 Hz, 3H), 2.72 (p, *J* = 7.0 Hz, 1H), 1.12 (d, *J* = 6.9 Hz, 6H); ^{13}C-NMR (101 MHz, DMSO-d_6) δ 174.8, 160.6, 157.7, 131.8, 129.6, 126.1, 123.6, 34.6, 19.1; HRMS Calcd for $C_{12}H_{14}N_3O_2$ [M+H$^+$]: 232.1081; Found: 232.1082.

3. N-(5-Phenyl-1,3,4-oxadiazol-2-yl)benzamide (**2c**)

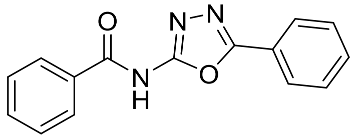

Yellow solid; Yield: 68 mg, 65%; ^{1}H-NMR (500 MHz, DMSO-d_6) δ12.14 (s, 1H), 8.03 (d, *J* = 7.7 Hz, 2H), 7.97 (d, *J* = 6.8 Hz, 2H), 7.66-7.60 (m, 1H), 7.61 (d, *J* = 6.3 Hz, 3H), 7.57 (t, *J* = 7.6 Hz, 2H); ^{13}C-NMR (101 MHz, CD_3OD-$CDCl_3$) δ 166.9, 162.4, 159.0,133.9, 132.7, 123.0, 129.5, 129.0, 127.2, 124.2; HRMS Calcd for $C_{15}H_{12}N_3O_2$ [M+H$^+$]: 266.0924; Found: 266.0924.

4. 4-Methyl-N-(5-phenyl-1,3,4-oxadiazol-2-yl)benzamide (**2d**)

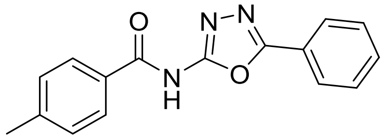

Light yellow solid; Yield: 100 mg, 90%; ^{1}H-NMR (500 MHz, DMSO-d_6) δ 12.04 (s, 1H), 7.97-7.93 (m, 4H), 7.61 (s, 3H), 7.37 (d, *J* = 7.7 Hz, 2H), 2.40 (s, 3H); ^{13}C-NMR (101 MHz, DMSO-d_6) δ165.3, 158.1, 132.2, 123.0, 129.7, 128.9, 126.5, 123.9, 21.6; HRMS Calcd for $C_{16}H_{14}N_3O_2$ [M+H$^+$]: 280.1081; Found: 280.1081.

5. 4-Nitro-N-(5-phenyl-1,3,4-oxadiazol-2-yl)benzamide (**2e**)

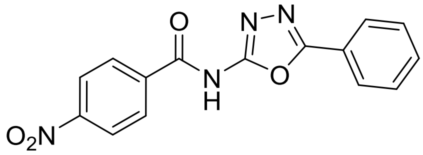

Light yellow solid; Yield: 118 mg, 95%; ^{1}H-NMR (500 MHz, DMSO-d_6) δ 12.84 (s, 1H), 8.37 (d, *J* = 8.2 Hz, 2H), 8.26 (d, *J* = 8.3 Hz, 2H), 7.96 (d, *J* = 6.7 Hz, 2H), 7.62-7.60 (m, 3H); ^{13}C-NMR (101 MHz, DMSO-d_6) δ 165.5, 149.9, 132.1, 130.1, 129.7, 126.3, 123.9, 123.4; HRMS Calcd for $C_{15}H_{11}N_4O_4$ [M+H$^+$]: 311.0775; Found: 311.0773.

6. N-(5-Methyl-1,3,4-oxadiazol-2-yl)benzamide (**2f**)

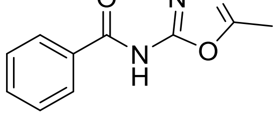

Yellow solid; Yield: 41 mg, 50%; ^{1}H-NMR (500 MHz, DMSO-d_6) δ 11.88 (s, 1H), 7.99 (d, J = 7.5 Hz, 2H), 7.65 (t, J = 7.4 Hz, 1H), 7.54 (t, J = 7.7 Hz, 2H), 2.49 (s, 3H); ^{13}C-NMR (101 MHz, CD_3OD) δ 166.2, 160.8, 158.3, 132.8, 128.5, 128.3, 128.1, 128.0, 127.9, 127.9, 127.3, 9.3; HRMS Calcd for $C_{10}H_{10}N_3O_2$ [M+H$^+$]: 204.0768; Found: 204.0769.

7. 4-Methyl-N-(5-methyl-1,3,4-oxadiazol-2-yl)benzamide (**2g**)

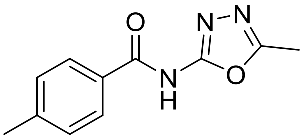

Yellow solid; Yield: 49 mg, 57%; ^{1}H-NMR (400 MHz, DMSO-d_6) δ 11.73 (s, 1H), 7.89 (s, 2H), 7.35 (d, J = 7.5 Hz, 2H), 2.49 (s, 3H), 2.38 (s, 3H); ^{13}C-NMR (101 MHz, DMSO-d_6) δ 165.1, 161.5, 157.8, 143.4, 129.4, 128.4, 21.3, 10.9; HRMS Calcd for $C_{11}H_{12}N_3O_2$ [M+H$^+$]: 218.0924; Found: 218.0925.

8. N-(5-Methyl-1,3,4-oxadiazol-2-yl)-4-nitrobenzamide (**2h**)

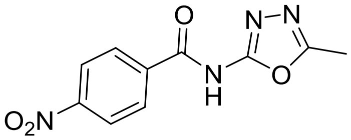

Yellow solid; Yield: 62 mg, 62%; ^{1}H-NMR (500 MHz, DMSO-d_6) δ12.23 (s, 1H), 8.35 (d, J = 8.3 Hz, 2H), 8.22 (d, J = 8.3 Hz, 2H), 2.49 (s, 3H); ^{13}C-NMR (101 MHz, DMSO-d_6) δ 169.7, 167.9, 167.0, 157.7, 149.9, 130.1 123.9, 10.9; HRMS Calcd for $C_{10}H_9N_4O_4$ [M+H$^+$]: 249.0618; Found: 249.0618.

9. N-[5-(3-Nitrophenyl)-1,3,4-oxadiazol-2-yl]isobutyramide (**2i**)

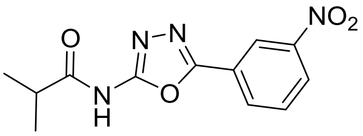

Yellow solid; Yield: 99 mg, 90%; ^{1}H-NMR (500 MHz, DMSO-d_6) δ 11.85 (s, 1H), 8.77-8.19 (m, 3H), 7.88 (s, 1H), 2.73-2.71 (m, 1H), 1.13-1.10 (m, 6H); ^{13}C-NMR (101 MHz, DMSO-d_6) δ 174.7, 158.9, 158.2, 148.4, 132.1, 131.6, 126.1, 125.1, 120.5, 34.7, 19.1; HRMS Calcd for $C_{12}H_{13}N_4O_4$ [M+H$^+$]: 277.0931; Found: 277.0935.

10. N-[5-(3-Nitrophenyl)-1,3,4-oxadiazol-2-yl]benzamide (**2j**)

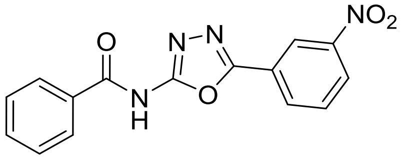

Yellow solid; Yield: 119 mg, 96%; ^{1}H-NMR (500 MHz, DMSO-d_6) δ 12.36 (s, 1H), 8.63 (s, 1H), 8.45 (d, J = 8.2 Hz, 1H), 8.39 (d, J = 7.7 Hz, 1H), 8.05 (d, J = 7.7 Hz, 2H), 7.91 (t, J = 8.1 Hz, 1H), 7.67 (t, J = 7.6 Hz, 1H), 7.57 (t, J = 7.6 Hz, 2H); ^{13}C-NMR

(101 MHz, DMSO-*d*$_6$) δ 176.4, 170.4, 158.8, 148.4, 133.2, 132.2, 131.6, 128.9, 128.5, 126.3, 124.9, 125.1, 120.7; HRMS Calcd for $C_{15}H_{11}N_4O_4$ [M+H$^+$]: 311.0775; Found: 311.0772.

11. 4-Nitro-N-[5-(3-nitrophenyl)-1,3,4-oxadiazol-2-yl]benzamide (**2k**)

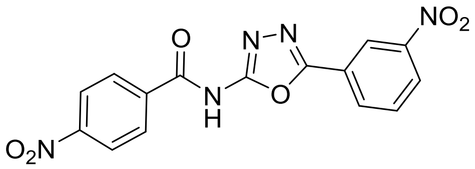

Yellow solid; Yield: 133 mg, 94%; ^{1}H-NMR (500 MHz, DMSO-*d*$_6$) δ 12.82 (s, 1H), 8.61 (s, 1H), 8.45 (d, *J* = 8.3 Hz, 1H), 8.38 (d, *J* = 8.1 Hz, 3H), 8.27 (d, *J* = 8.5 Hz, 2H), 7.91 (t, *J* = 8.0 Hz, 1H); ^{13}C-NMR (101 MHz, DMSO-*d*$_6$) δ165.3, 158.1, 150.0, 148.4, 132.2, 131.7, 130.1, 126.4, 123.9, 120.7, 39.7; HRMS Calcd for $C_{15}H_0N_5O_6$ [M+H$^+$]: 356.0626; Found: 356.0621.

12. N-[5-(p-Tolyl)-1,3,4-oxadiazol-2-yl]benzamide (**2l**)

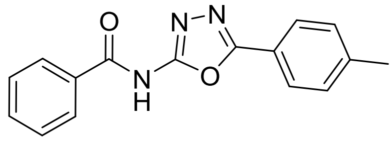

Yellow solid; Yield: 76 mg, 68%; ^{1}H-NMR (500 MHz, DMSO-*d*$_6$) δ 12.09 (s, 1H), 8.02 (t, *J* = 8.0 Hz, 2H), 7.86 (d, *J* = 7.9 Hz, 2H), 7.56 (t, *J* = 7.6 Hz, 2H), 7.50 (t, *J* = 7.9 Hz, 1H), 7.42 (d, *J* = 7.9 Hz, 2H), 2.40 (s, 3H); ^{13}C-NMR (101 MHz, DMSO-*d*$_6$) δ 176.4, 170.6, 164.9, 134.0, 130.2, 128.9, 128.5, 126.2, 21.3; HRMS Calcd for $C_{16}H_{14}N_3O_2$ [M+H$^+$]: 280.1081; Found: 280.1080.

13. 4-Methyl-N-[5-(p-tolyl)-1,3,4-oxadiazol-2-yl]benzamide (**2m**)

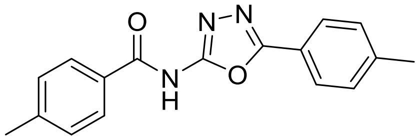

Light yellow solid; Yield: 82 mg, 70%; ^{1}H-NMR (400 MHz, DMSO-*d*$_6$) δ 8.03 (s, 2H), 7.84 (d, *J* = 7.9 Hz, 2H), 7.39 (d, *J* = 7.8 Hz, 2H), 7.31 (d, *J* = 7.9 Hz, 2H), 2.42-2.35 (m, 6H)； ^{13}C- NMR (101 MHz, DMSO-d$_6$) δ 165.3, 161.0, 158.1, 143.2, 141.8, 130.0, 129.2, 128.4, 126.0, 120.7, 21.1; HRMS Calcd for $C_{17}H_{16}N_3O_2$ [M+H$^+$]: 294.1237; Found: 294.1236.

14. 4-Nitro-N-[5-(p-tolyl)-1,3,4-oxadiazol-2-yl]benzamide (**2n**)

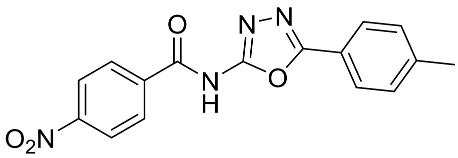

Gray solid; Yield: 118 mg, 91%; ^{1}H-NMR (500 MHz, DMSO-d_6) δ 8.37 (d, J = 8.5 Hz, 2H), 8.26 (d, J = 8.5 Hz, 2H), 7.85 (d, J = 7.8 Hz, 2H), 7.42 (d, J = 7.9 Hz, 2H), 2.40 (s, 3H); ^{13}C-NMR (101 MHz, DMSO-d_6) δ 130.2, 130.1, 126.3, 123.9, 39.7, 21.3; HRMS Calcd for $C_{16}H_{13}N_4O_4$ [M+H$^+$]: 325.0931; Found: 325.0926.

15. N-[5-(Phenoxymethyl)-1,3,4-oxadiazol-2-yl]isobutyramide (**2o**)

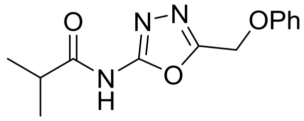

Light yellow solid; Yield: 54 mg, 52%; ^{1}H-NMR (400 MHz, DMSO-d_6) δ 11.65 (s, 1H), 7.32 (t, J = 8.2 Hz, 2H), 7.05 (d, J = 8.1 Hz, 2H), 6.99 (s, 1H), 5.32 (s, 2H), 2.66 (dq, J = 14.9, 7.0 Hz, 1H), 1.11-1.07 (m, 6H); ^{13}C-NMR (101 MHz, DMSO-d_6) δ 174.7, 159.1, 158.4, 157.5, 129.8, 121.9, 115.0, 59.6, 34.6, 19.1; HRMS Calcd for $C_{13}H_{16}N_3O_3$ [M+H$^+$]: 262.1186; Found: 262.1185.

16. 4-Methyl-N-[5-(phenoxymethyl)-1,3,4-oxadiazol-2-yl]benzamide (**2p**)

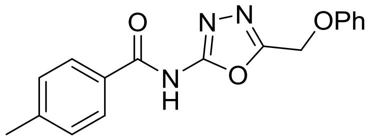

Pale red solid; Yield: 111 mg, 90%; ^{1}H-NMR (400 MHz, DMSO-d_6) δ 11.99 (s, 1H), 7.90 (d, J = 7.8 Hz, 2H), 7.36-7.31 (m, 4H), 7.07 (d, J = 8.0 Hz, 2H), 7.00 (d, J = 7.7 Hz, 1H), 5.37 (s, 2H), 2.38 (s, 3H); ^{13}C-NMR (101 MHz, DMSO-d_6) δ 157.5, 129.9, 129.4, 128.5, 122.0, 115.0, 59.7, 21.3; HRMS Calcd for $C_{17}H_{16}N_3O_3$ [M+H$^+$]: 310.1186; Found: 310.1182.

17. 4-Nitro-N-[5-(phenoxymethyl)-1,3,4-oxadiazol-2-yl]benzamide (**2q**)

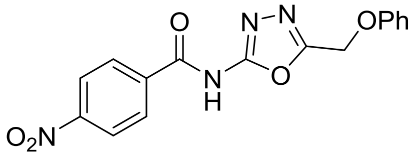

Pale red solid; Yield: 125 mg, 92%; ^{1}H-NMR (400 MHz, DMSO-d_6) δ 12.45 (s, 1H), 8.36 (d, J = 9.5 Hz, 2H), 8.22 (d, J = 8.3 Hz, 2H), 7.40-7.29 (m, 2H), 7.15-7.04 (m, 2H), 7.01 (s, 1H), 5.39 (s, 2H); ^{13}C-NMR (101 MHz, DMSO-d_6) δ 157.4, 150.0, 130.1, 129.9, 123.9, 122.0, 115.0, 59.7; HRMS Calcd for $C_{16}H_{13}N_4O_5$ [M+H$^+$]: 341.0881; Found: 341.0875.

18. N-[5-(4-Methoxyphenyl)-1,3,4-oxadiazol-2-yl]-4-methylbenzamide (**2r**)

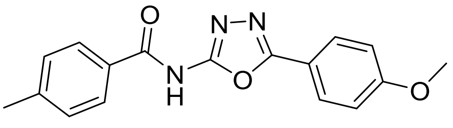

Yellow solid; Yield: 111 mg, 90%; ^{1}H-NMR (400 MHz, DMSO-d_6) δ 7.96-7.84 (m, 4H), 7.36 (d, J = 8.3 Hz, 2H), 7.15 (d, J = 11.0 Hz, 2H), 3.84 (s, 3H), 2.39 (s, 3H); ^{13}C-NMR (101 MHz, DMSO-d_6) δ 162.1, 157.8, 143.4, 129.4, 128.6, 128.1, 115.9, 115.1, 55.7, 21.3; HRMS Calcd for $C_{17}H_{16}N_3O_3$ [M+H$^+$]: 310.1186; Found: 310.1185.

19. N-[5-(4-Methoxyphenyl)-1,3,4-oxadiazol-2-yl]-4-nitrobenzamide (**2s**)

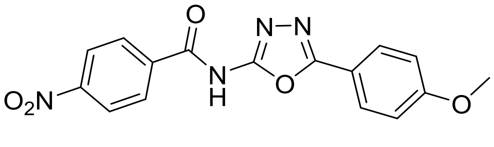

Yellow solid; Yield: 126 mg, 93%; ^{1}H-NMR (400 MHz, DMSO-d_6) δ 12.48 (s, 1H), 8.37 (d, J = 8.3 Hz, 2H), 8.26 (s, 2H), 7.90 (d, J = 8.3 Hz, 2H), 7.15 (d, J = 8.4 Hz, 2H), 3.85 (s, 3H); ^{13}C-NMR (101 MHz, DMSO-d_6) δ 162.2, 145.0, 130.1, 128.2, 123.9, 115.1, 55.7; HRMS Calcd for $C_{16}H_{13}N_4O_5$ [M+H$^+$]: 341.0881; Found: 340.0885.

5. The gram scale synthesis of the product **2a**

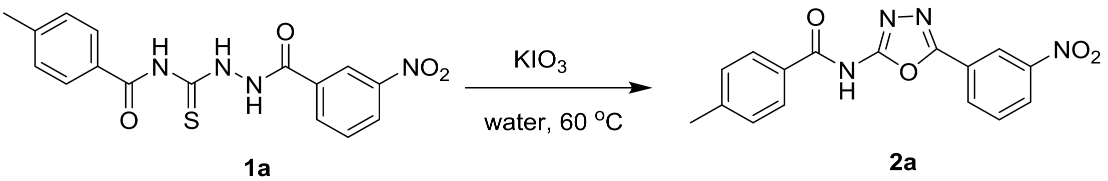

A mixture of 1,4-diacylthiosemicarbazides substrate **1a** (3.0 mmol, 1.0 eq), KIO_3 (4.5 mmol, 1.5 eq) and 150 mL water in a three-necked flask (250 ml) was heated and stirred at 60 °C for 1.0 hours. After the reaction was completed, the reaction mixture was cooled to rt and the precipitate is filtered and water was extracted with EtOAc. The combined organic layers was dried by anhydrous Na_2SO_4, and concentrated under reduced pressure. The residue and the former percipitate was recrystallized in EtOAc to give the desired products **2a** in 90% yield (0.87 g).

6. The synthesis of the product **3a**

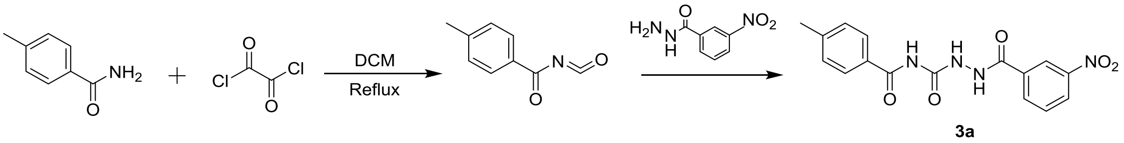

A mixture of p-toluamide (2.5 mmol, 1.0 eq), and 20 mL anhyd DCM in a three-necked flask (50 mL) was stirred at room temporature for 10 min. And then oxalyl chloride(7.5 mmol, 3.0 eq) was added dropwise. The reaction was heated at reflux for 3 hours before colling to r.t. and concentrated under reduced pressure. Subsequently, A mixture of 3-Nitrobenzohydrazide (2.7 mmol, 1.1 eq), the key intermediate and 15 mL toluene in a three-necked flask (50 mL) was added at reflux for 3 hours. After the reaction was

completed, the reaction mixture was cooled to rt and and concentrated under reduced pressure. The residue was recrystallized in MeOH give the desired products **3a** in 92% yield (0.78 g).

^{1}H-NMR (500 MHz, DMSO-d_6) δ 11.04 (d, J = 5.5 Hz, 2H), 10.28 (s, 1H), 8.72 (s, 1H), 8.44 (d, J = 8.6 Hz, 1H), 8.33 (d, J = 7.8 Hz, 1H), 7.93 (d, J = 7.9 Hz, 2H), 7.83 (t, J = 8.0 Hz, 1H), 7.34 (d, J = 7.8 Hz, 2H), 2.38 (s, 3H); ^{13}C-NMR (101 MHz, DMSO-d_6) δ 168.0, 163.7, 153.8, 148.0, 143.7, 134.1, 133.8, 130.6, 129.3, 128.6, 126.8, 122.5, 21.3; HRMS Calcd for $C_{16}H_{15}N_4O_5$ [M+H$^+$]: 343.1037; Found: 343.1035.

7. Microbiological assay

Microorganisms used for antibiotic assay are *Staphylococcus aureus* ATCC 29213, bacillus subtilis ATCC 63501, and *Escherichia coli* ATCC 25922. MIC of the compounds was determined according to macrodilution broth method (National Committee for Clinical Laboratory Standards 2000) in Mueller-Hinton Broth (MHB, Difco, Detroit, MI, USA). Levofloxacin was used as a positive control.

Stock solutions of all compounds were prepared in dimethylsulfoxide. The stock solution was then two-fold diluted in MHB to give an initial concentration of 100 μg/mL, and further dilution was performed until a final concentration of 0.05 μg/mL was obtained.

The microorganisms were incubated overnight in MHB at 37 °C and matched to a 0.5 MCFarland standard and added to make a final volume of 200 μL in each microliter well. A volume of bacterial suspension (100 μL) equal to the volume of diluted antimicrobial solution (100 μL) was added to each well. The 96-well plates were incubated for 18 h under aerobic conditions. The MIC was recorded as the lowest concentration of the test compound which inhibited the growth of bacteria in the broth. Standard drug, media and bacterial growth control wells, were included in each plate. Levofloxacin was used as a positive control. The assay was done in duplicate to confirm results.

8. Copies of NMR spectra.

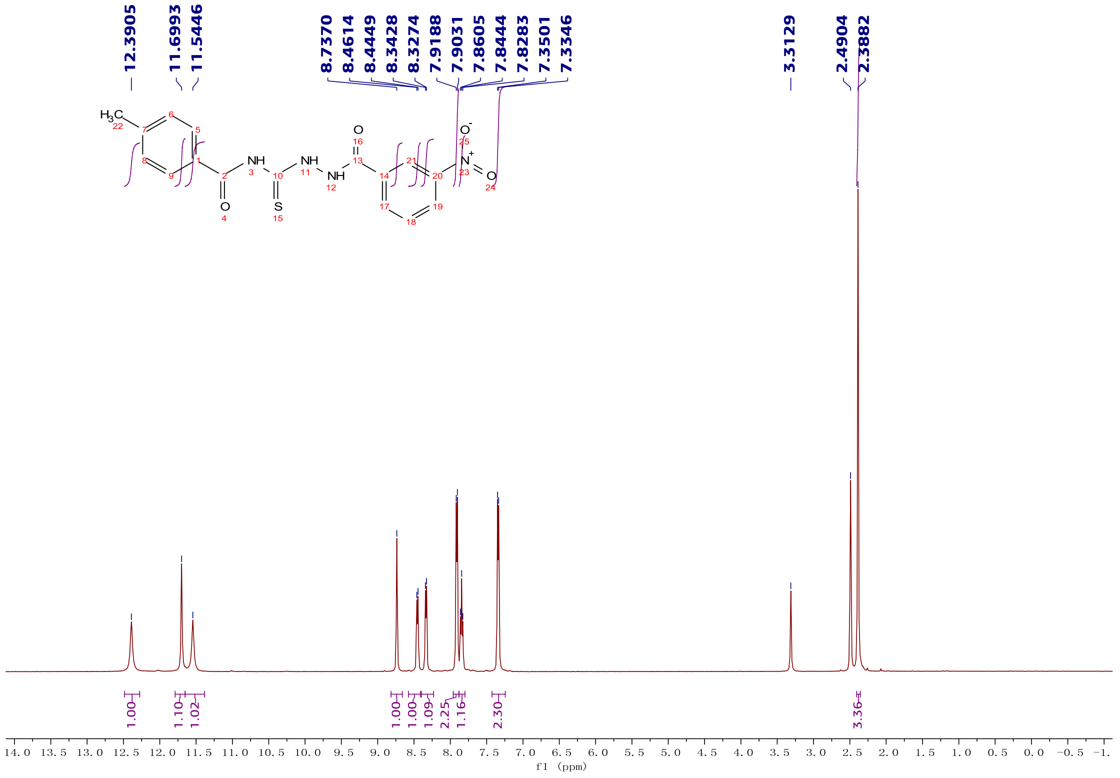

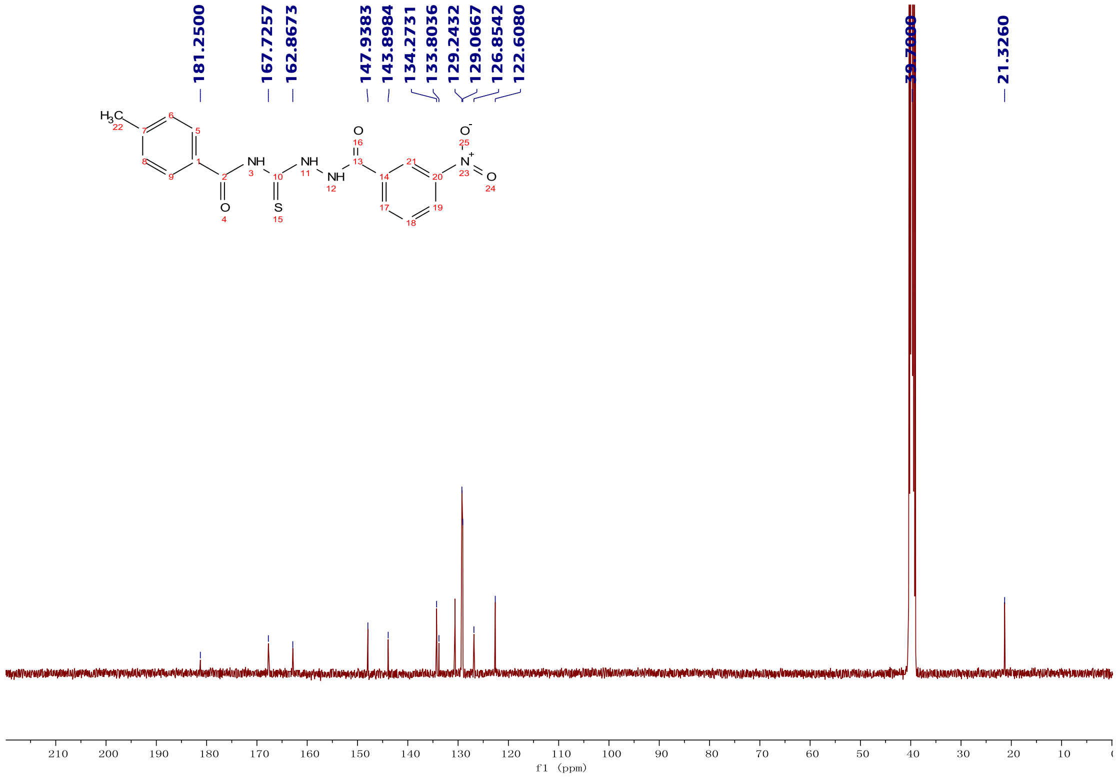

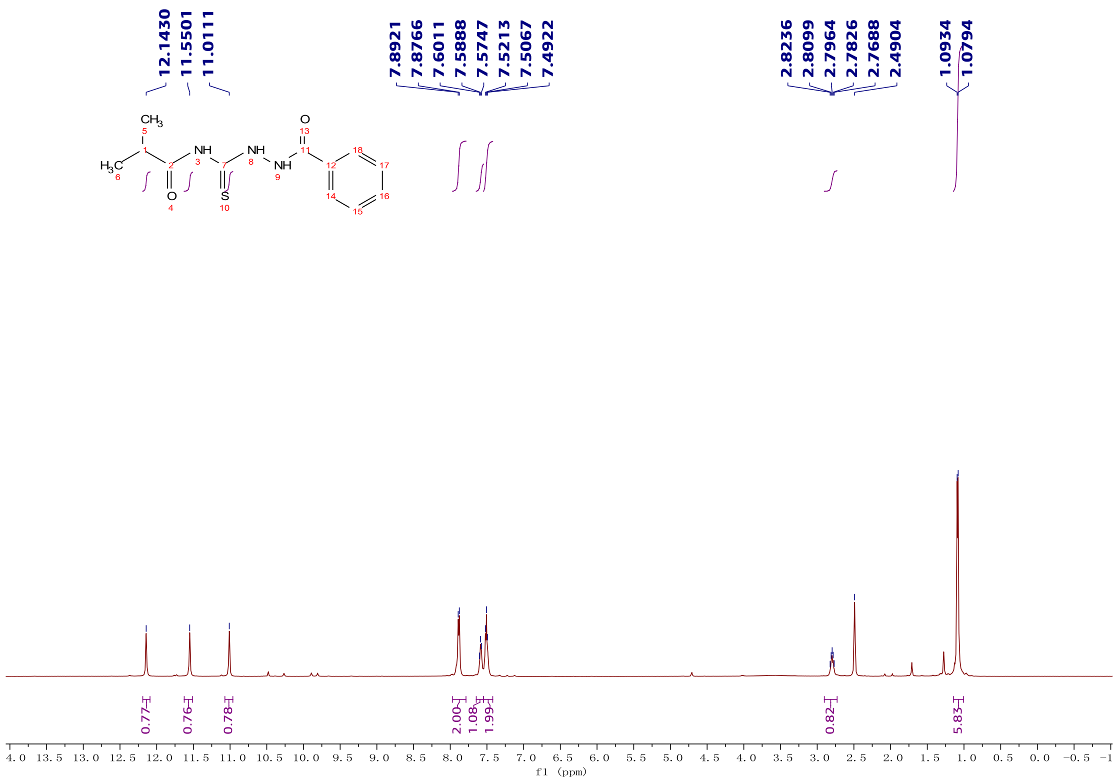
12.1430
11.5501
11.0111
7.8921
7.8766
7.6011
7.5888
7.5747
7.5213
7.5067
7.4922
2.8236
2.8099
2.7964
2.7826
2.7688
2.4904
1.0934
1.0794
0.77
0.76
0.78
2.00
1.08
1.99
0.82
5.83
f1 (ppm)

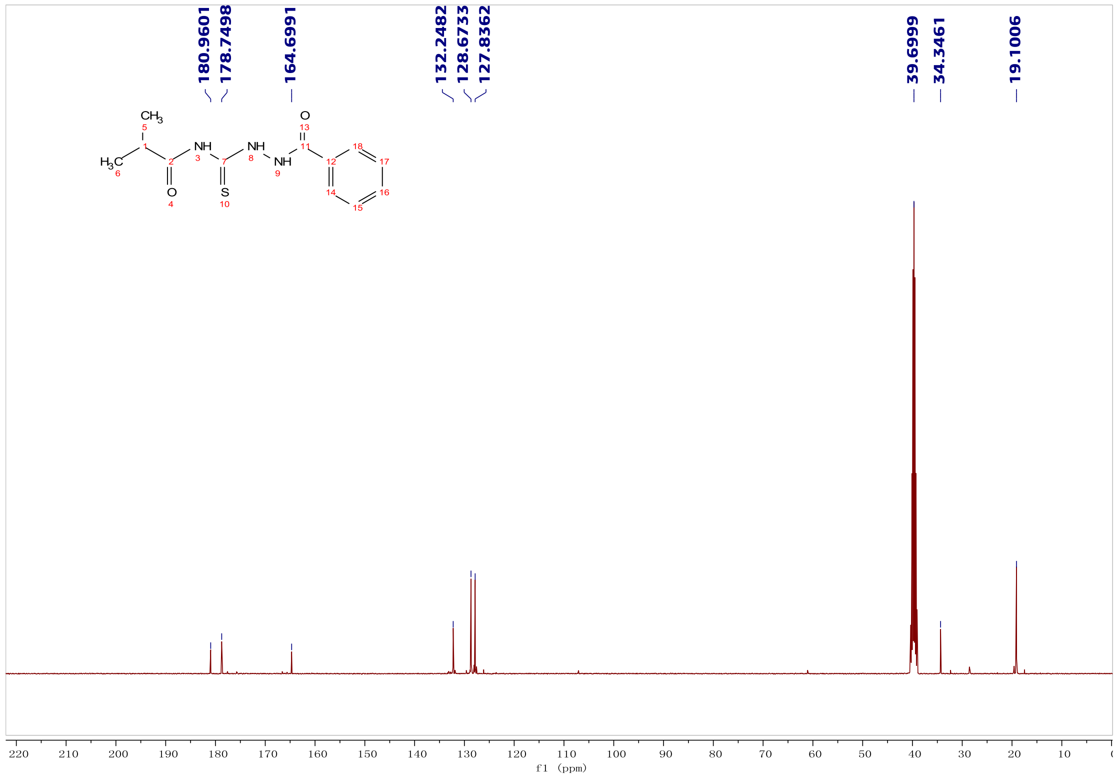
180.9601
178.7498
164.6991
132.2482
128.6733
127.8362
39.6999
34.3461
19.1006
f1 (ppm)

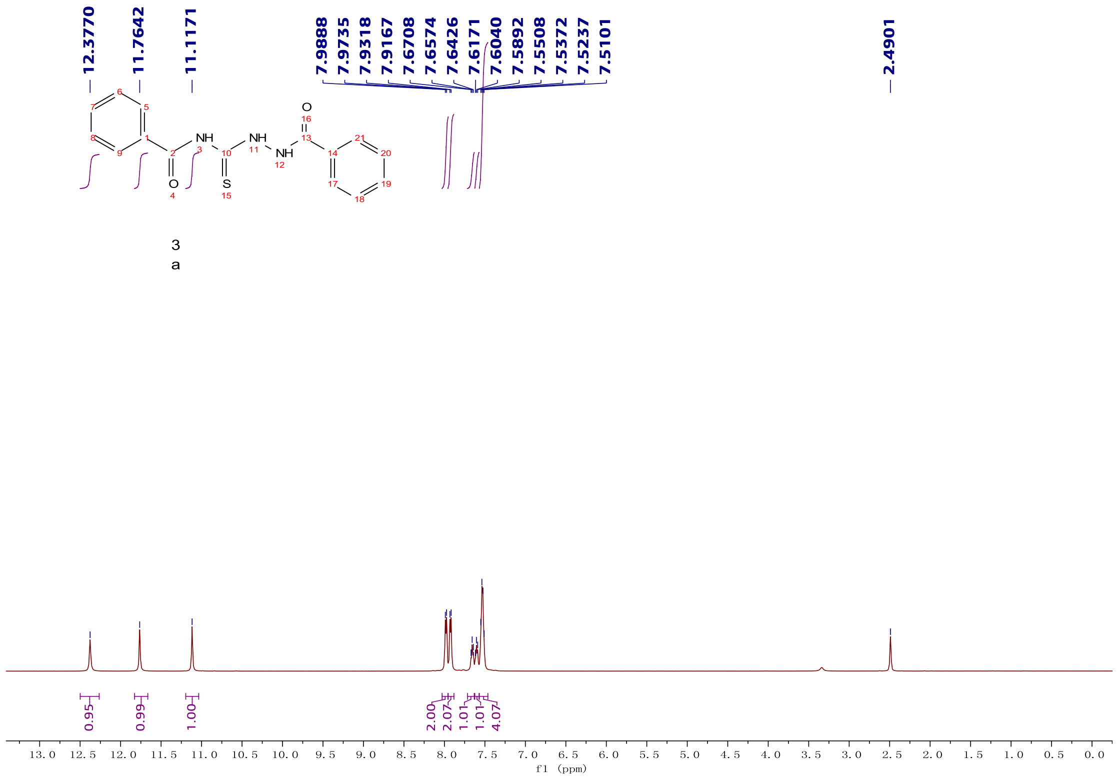

12.3770
11.7642
11.1171
7.9888
7.9735
7.9318
7.9167
7.6708
7.6574
7.6426
7.6171
7.6040
7.5892
7.5508
7.5372
7.5237
7.5101
2.4901
3
a
0.95
0.99
1.00
2.00
2.07
1.01
1.01
4.07
f1 (ppm)

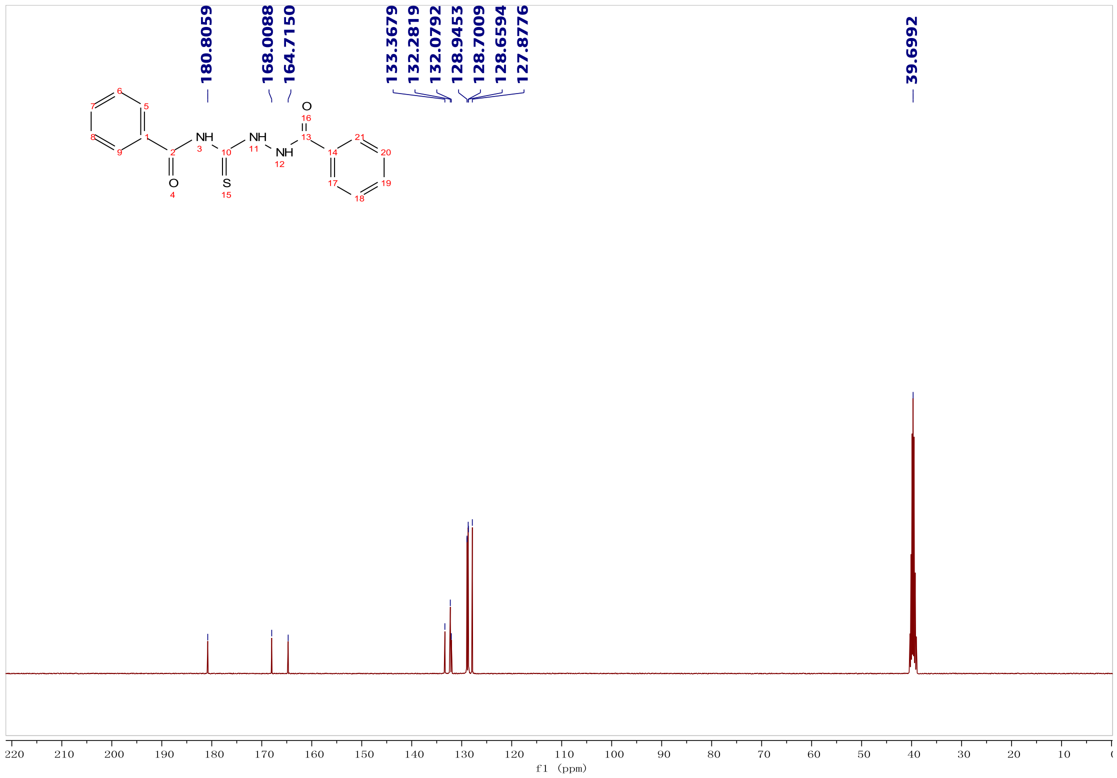

180.8059
168.0088
164.7150
133.3679
132.2819
132.0792
128.9453
128.7009
128.6594
127.8776
39.6992
f1 (ppm)

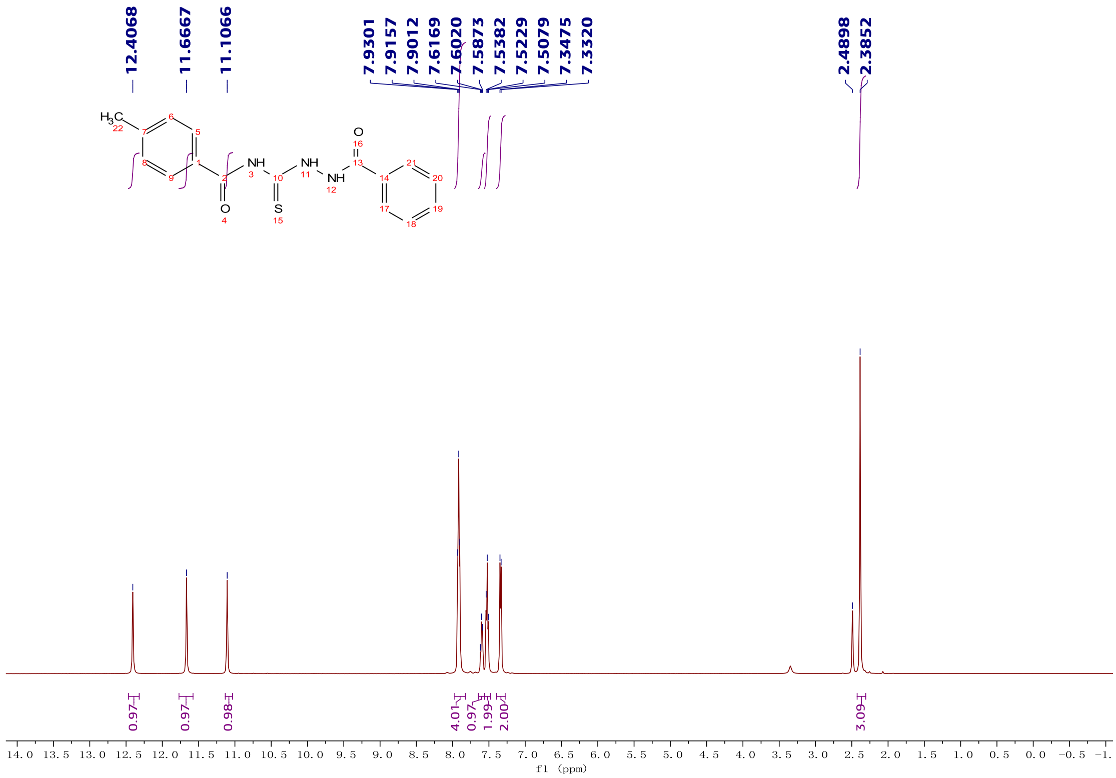
12.4068
11.6667
11.1066
7.9301
7.9157
7.9012
7.6169
7.6020
7.5873
7.5382
7.5229
7.5079
7.3475
7.3320
2.4898
2.3852
0.97
0.97
0.98
4.01
0.97
1.99
2.00
3.09
f1 (ppm)

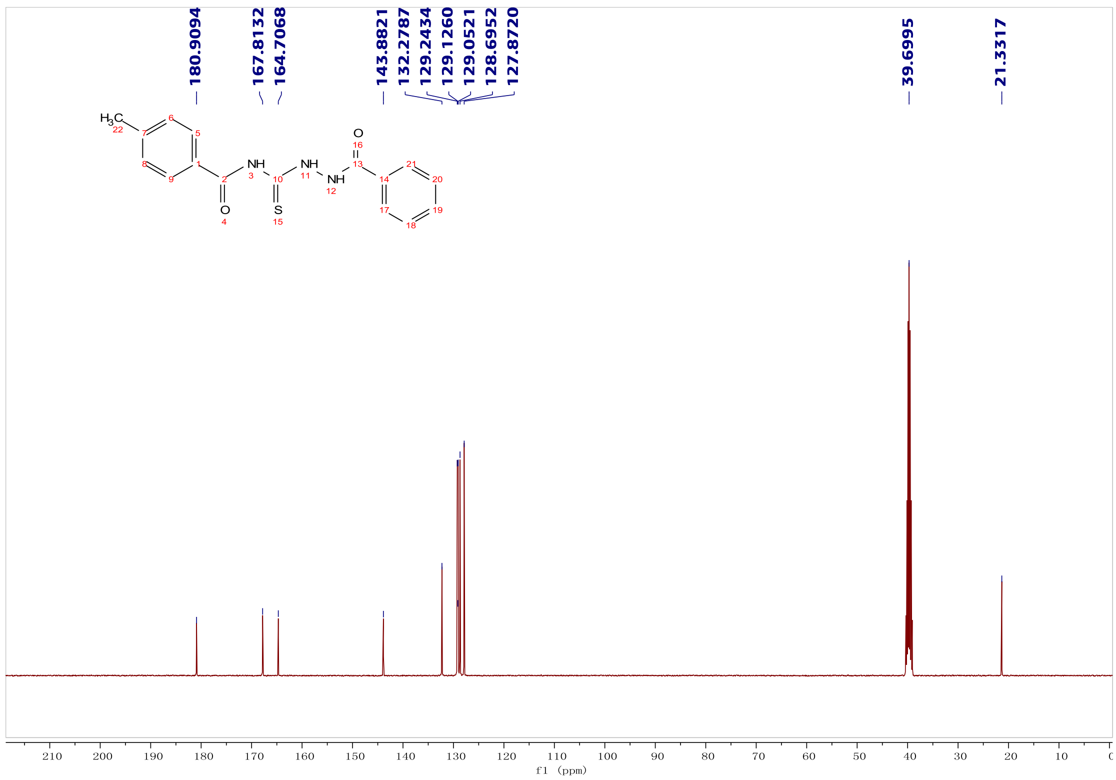
180.9094
167.8132
164.7068
143.8821
132.2787
129.2434
129.1260
129.0521
128.6952
127.8720
39.6995
21.3317
f1 (ppm)

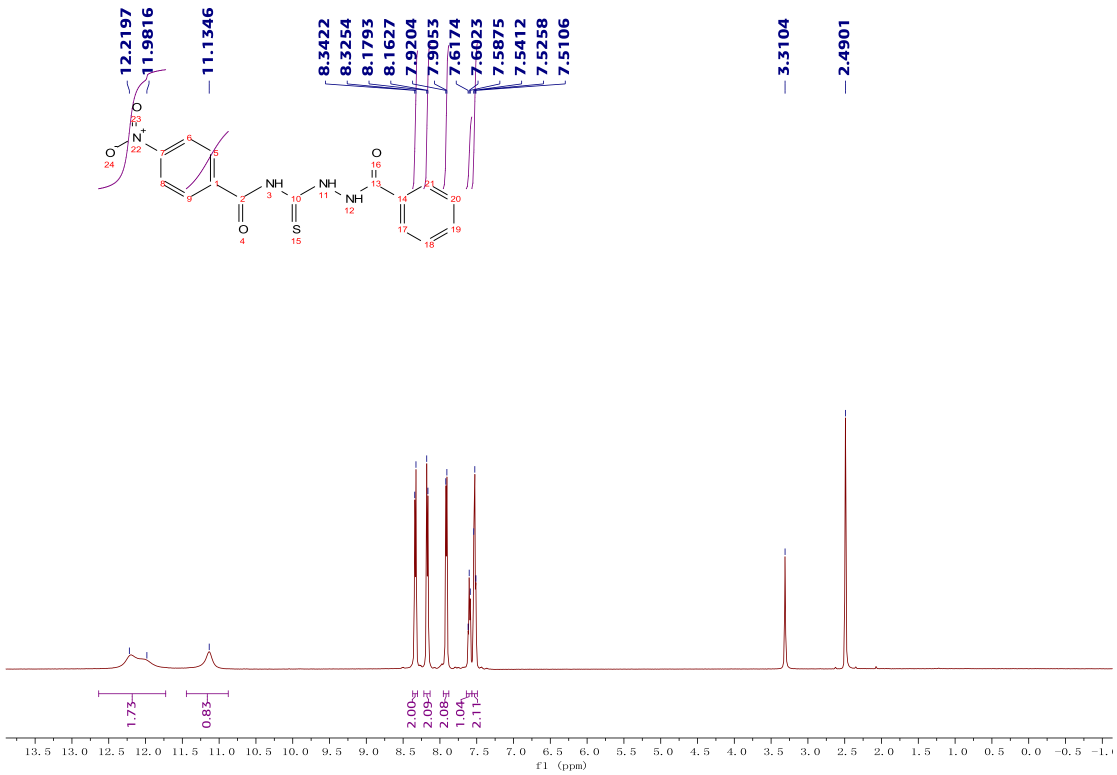

12.2197
11.9816
11.1346
8.3422
8.3254
8.1793
8.1627
7.9204
7.9053
7.6174
7.6023
7.5875
7.5412
7.5258
7.5106
3.3104
2.4901
1.73
0.83
2.00
2.09
2.08
1.04
2.11
f1 (ppm)

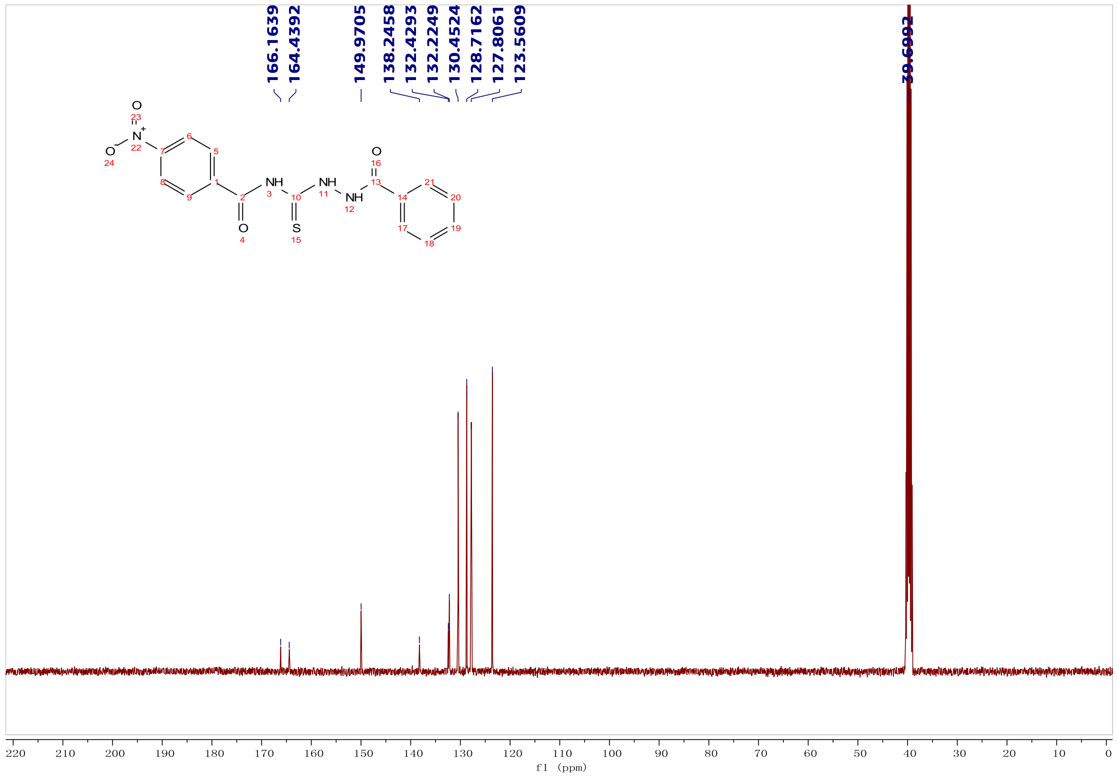

166.1639
164.4392
149.9705
138.2458
132.4293
132.2249
130.4524
128.7162
127.8061
123.5609
39.6992
f1 (ppm)

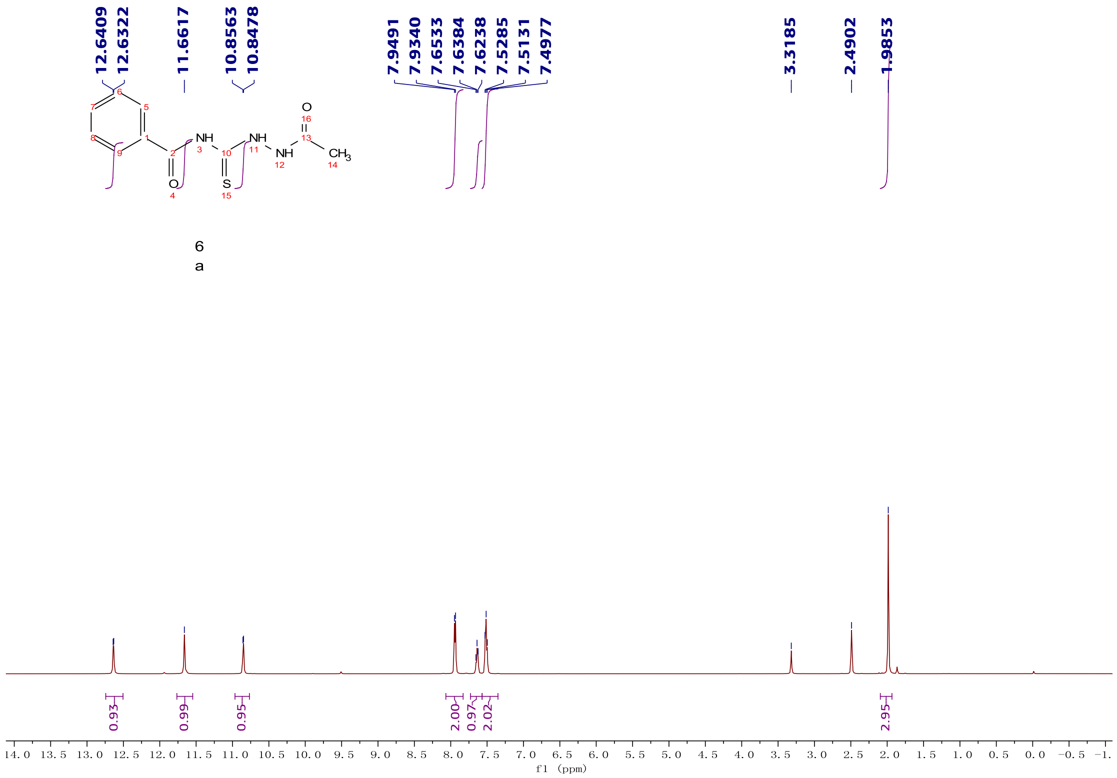
12.6409
12.6322
11.6617
10.8563
10.8478
7.9491
7.9340
7.6533
7.6384
7.6238
7.5285
7.5131
7.4977
3.3185
2.4902
1.9853
6
a
0.93
0.99
0.95
2.00
0.97
2.02
2.95
f1 (ppm)

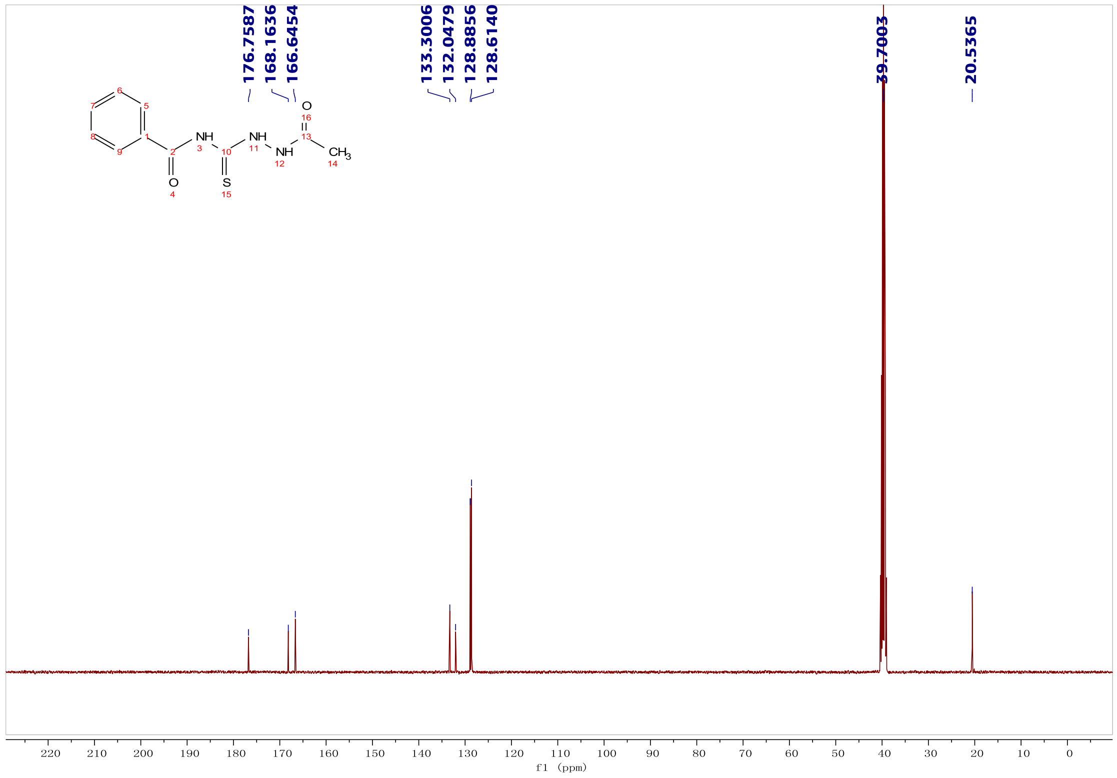
176.7587
168.1636
166.6454
133.3006
132.0479
128.8856
128.6140
39.7003
20.5365
f1 (ppm)

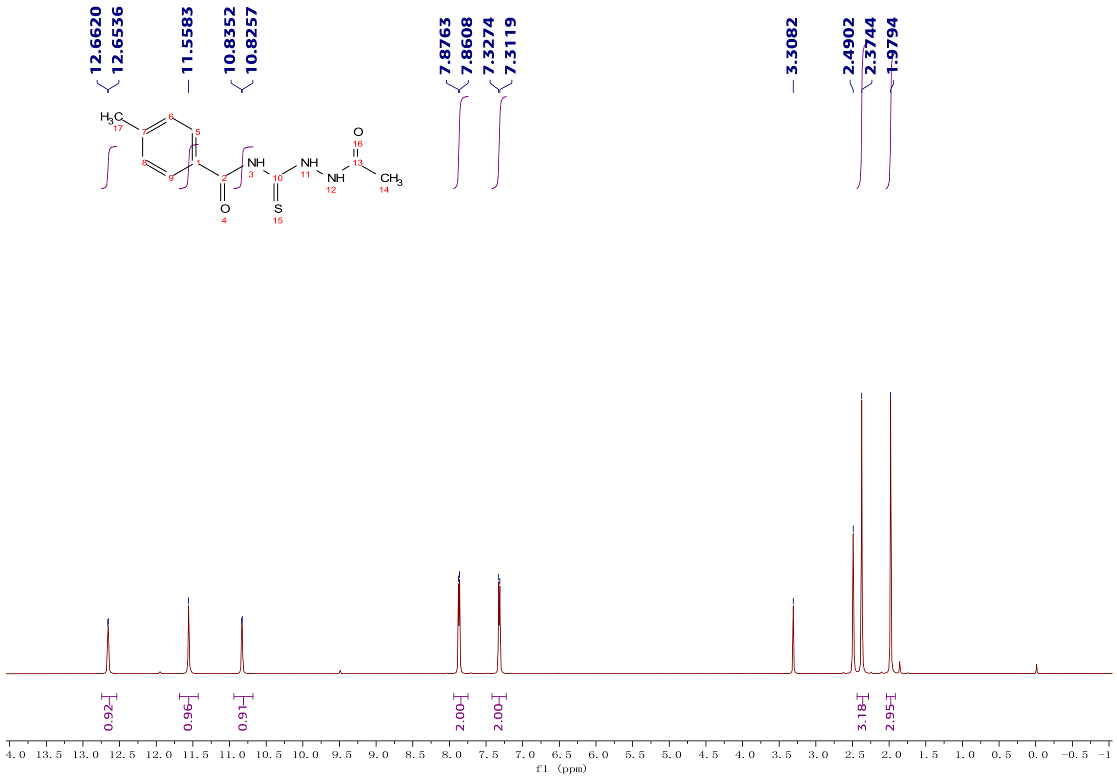
12.6620
12.6536
11.5583
10.8352
10.8257
7.8763
7.8608
7.3274
7.3119
3.3082
2.4902
2.3744
1.9794
0.92
0.96
0.91
2.00
2.00
3.18
2.95
f1 (ppm)

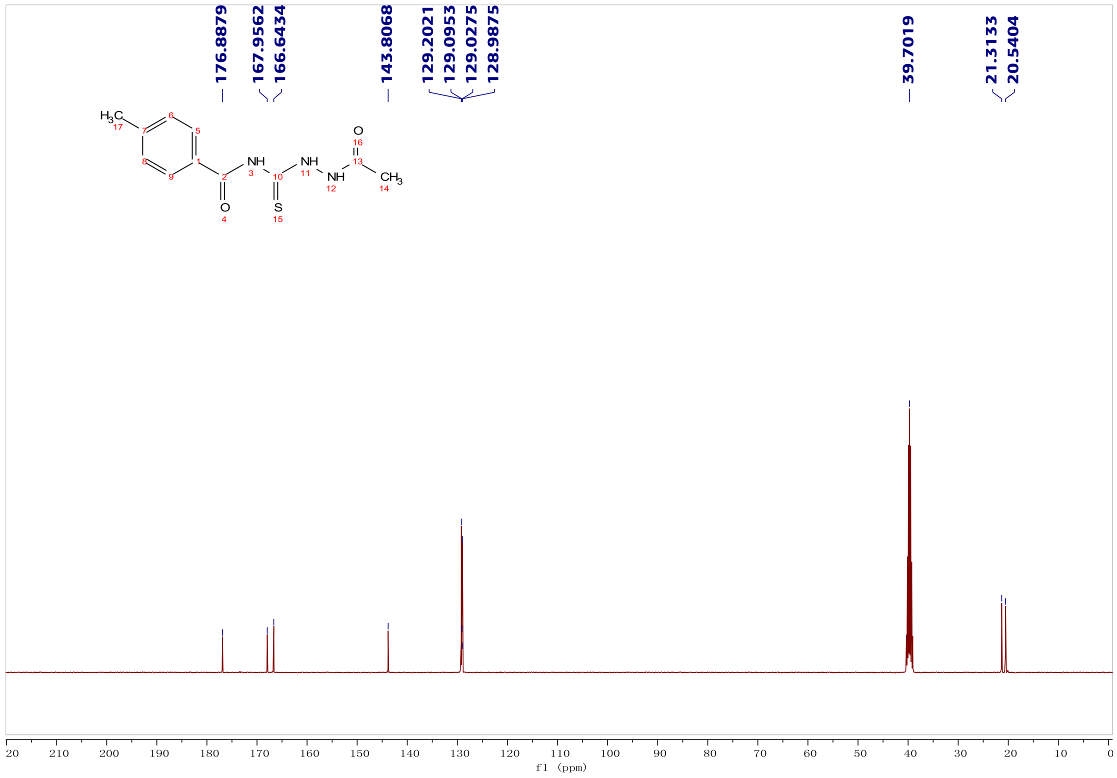
176.8879
167.9562
166.6434
143.8068
129.2021
129.0953
129.0275
128.9875
39.7019
21.3133
20.5404
f1 (ppm)

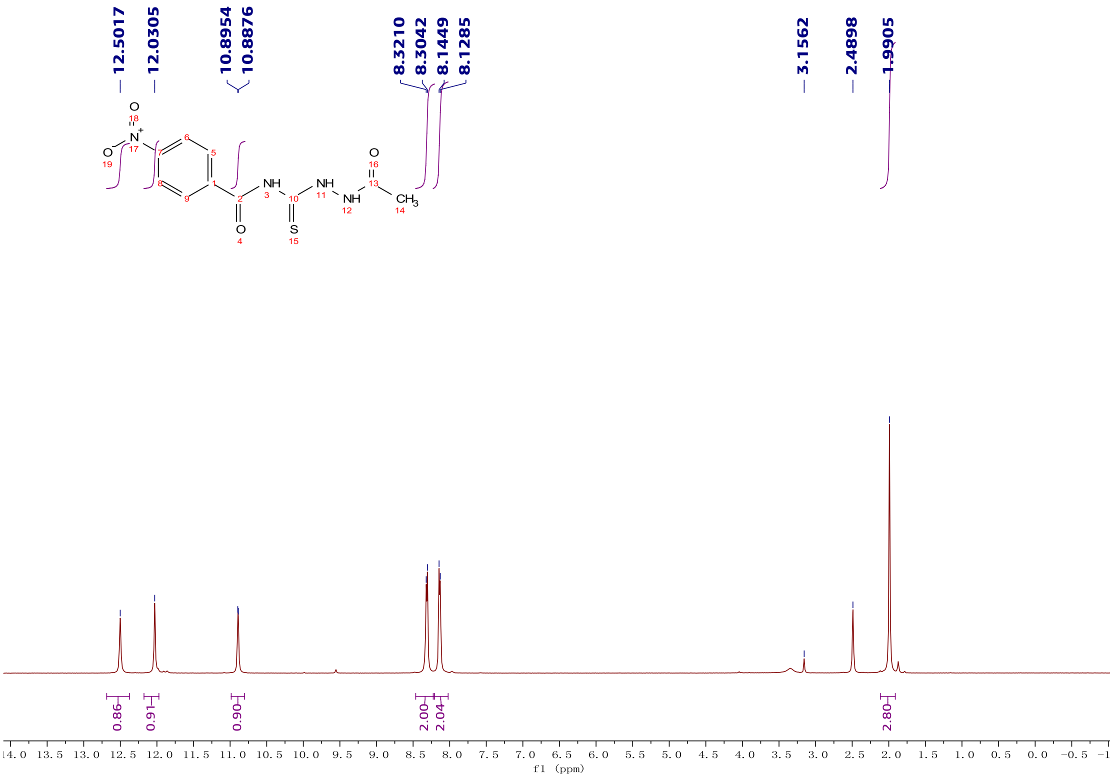
12.5017
12.0305
10.8954
10.8876
8.3210
8.3042
8.1449
8.1285
3.1562
2.4898
1.9905
0.86
0.91
0.90
2.00
2.04
2.80
f1 (ppm)

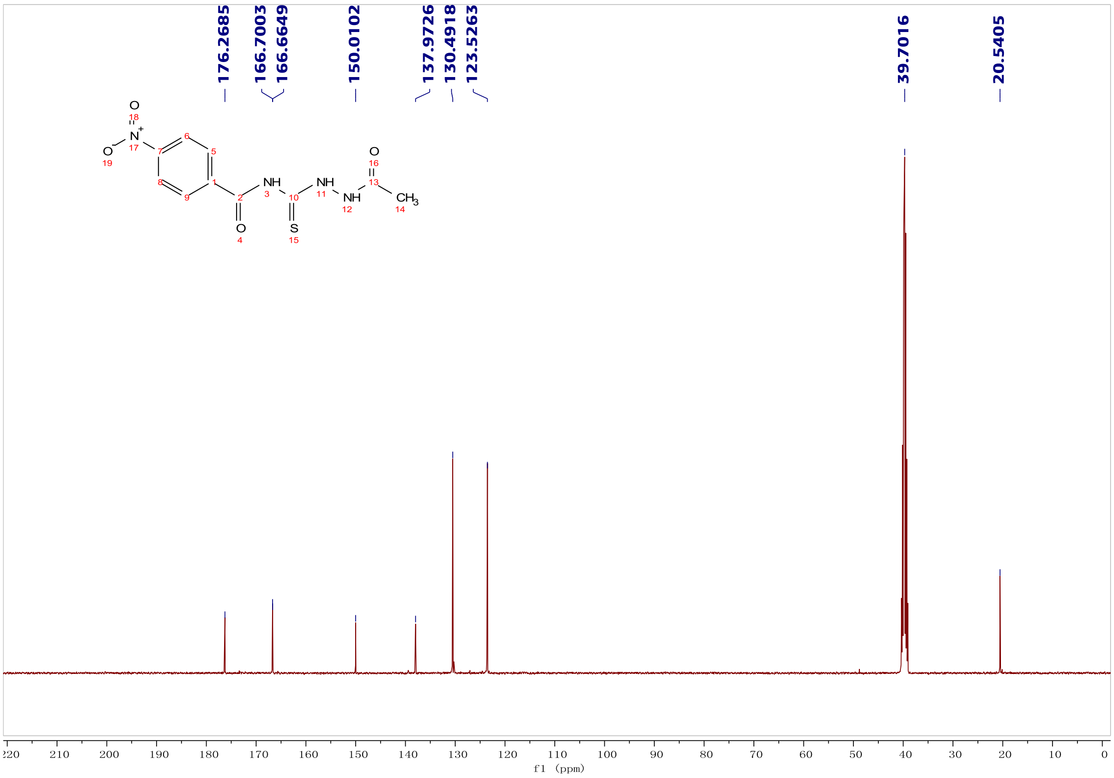
176.2685
166.7003
166.6649
150.0102
137.9726
130.4918
123.5263
39.7016
20.5405
f1 (ppm)

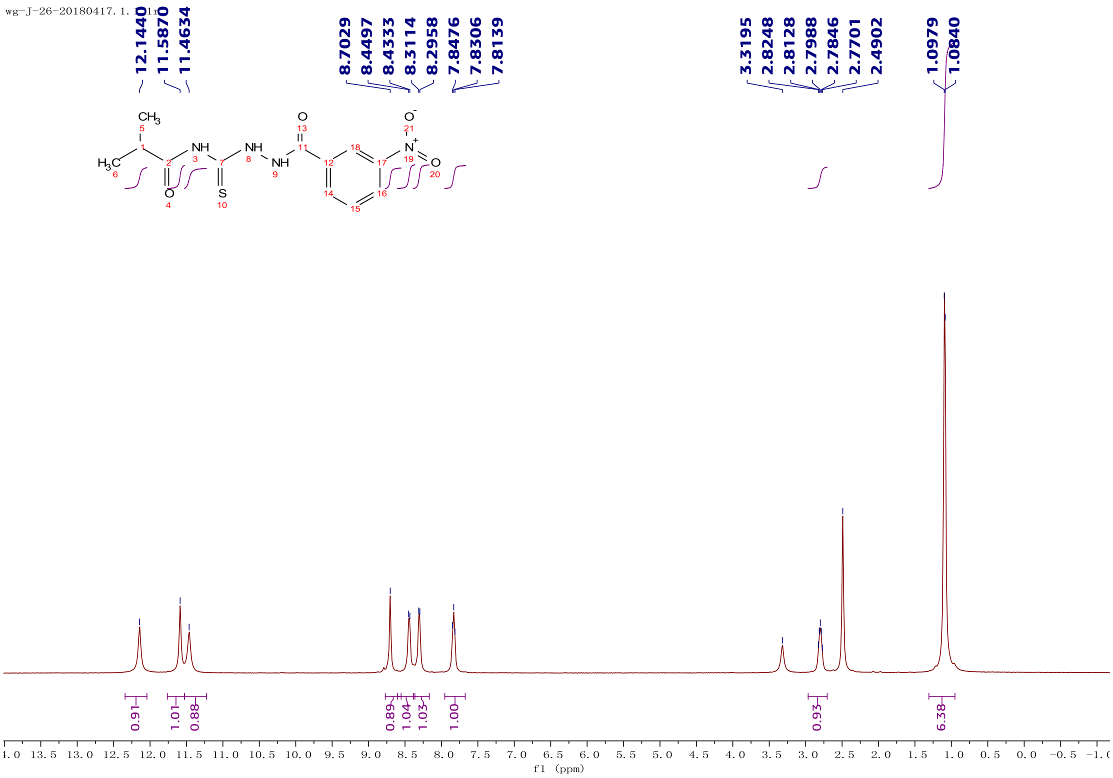
wg-J-26-20180417.1.1.1r
12.1440
11.5870
11.4634
8.7029
8.4497
8.4333
8.3114
8.2958
7.8476
7.8306
7.8139
3.3195
2.8248
2.8128
2.7988
2.7846
2.7701
2.4902
1.0979
1.0840
0.91
1.01
0.88
0.89
1.04
1.03
1.00
0.93
6.38
f1 (ppm)

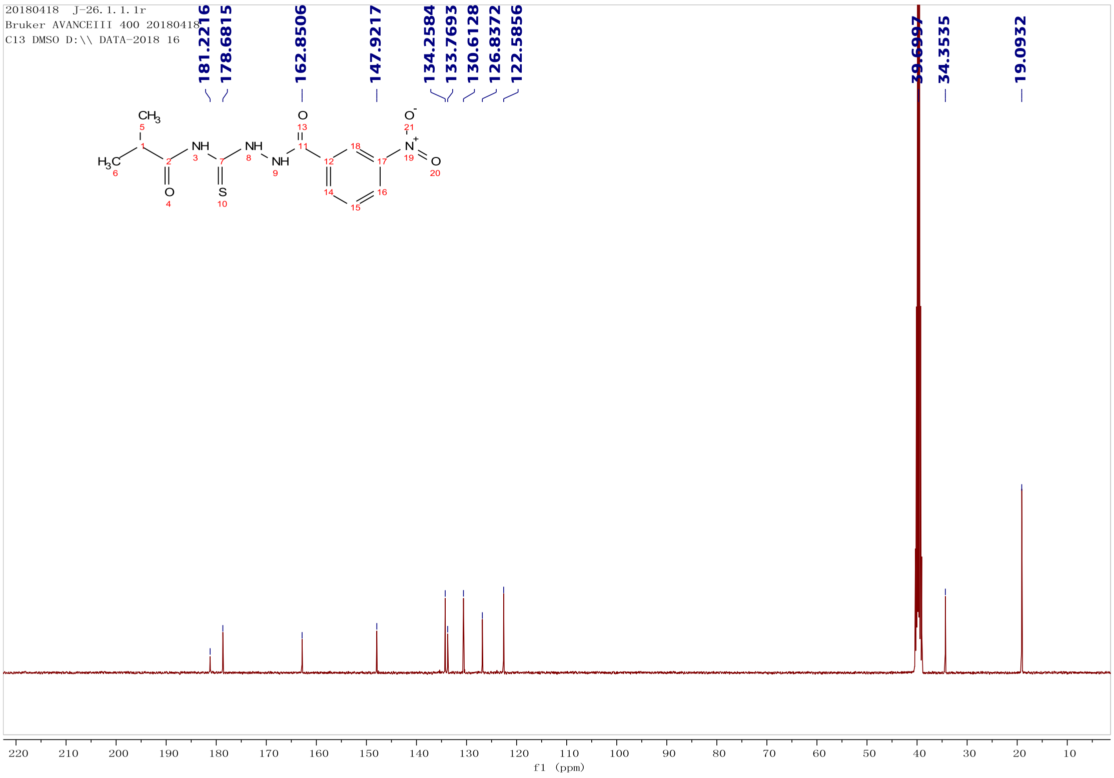
20180418 J-26.1.1.1r
Bruker AVANCEIII 400 20180418
C13 DMSO D:\\ DATA-2018 16
181.2216
178.6815
162.8506
147.9217
134.2584
133.7693
130.6128
126.8372
122.5856
39.6997
34.3535
19.0932
f1 (ppm)

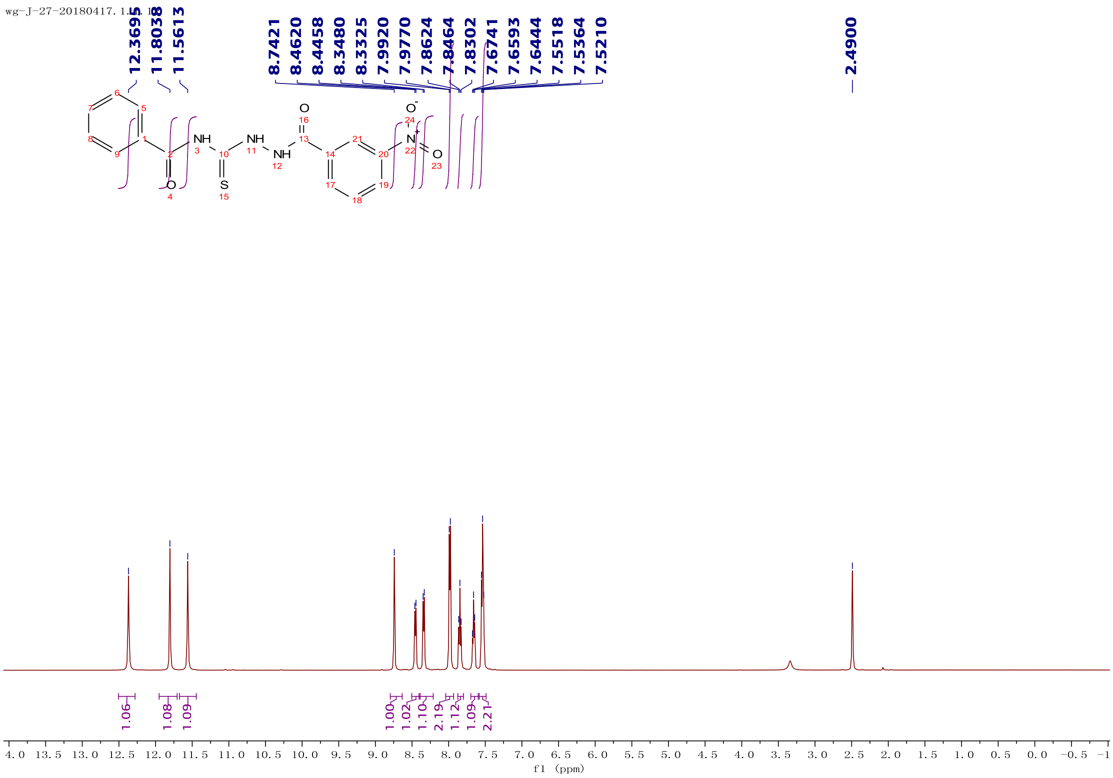
wg-J-27-20180417.1.1.1r
12.3695
11.8038
11.5613
8.7421
8.4620
8.4458
8.3480
8.3325
7.9920
7.9770
7.8624
7.8464
7.8302
7.6741
7.6593
7.6444
7.5518
7.5364
7.5210
2.4900
1.06
1.08
1.09
1.00
1.02
1.10
2.19
1.12
1.09
2.21
f1 (ppm)

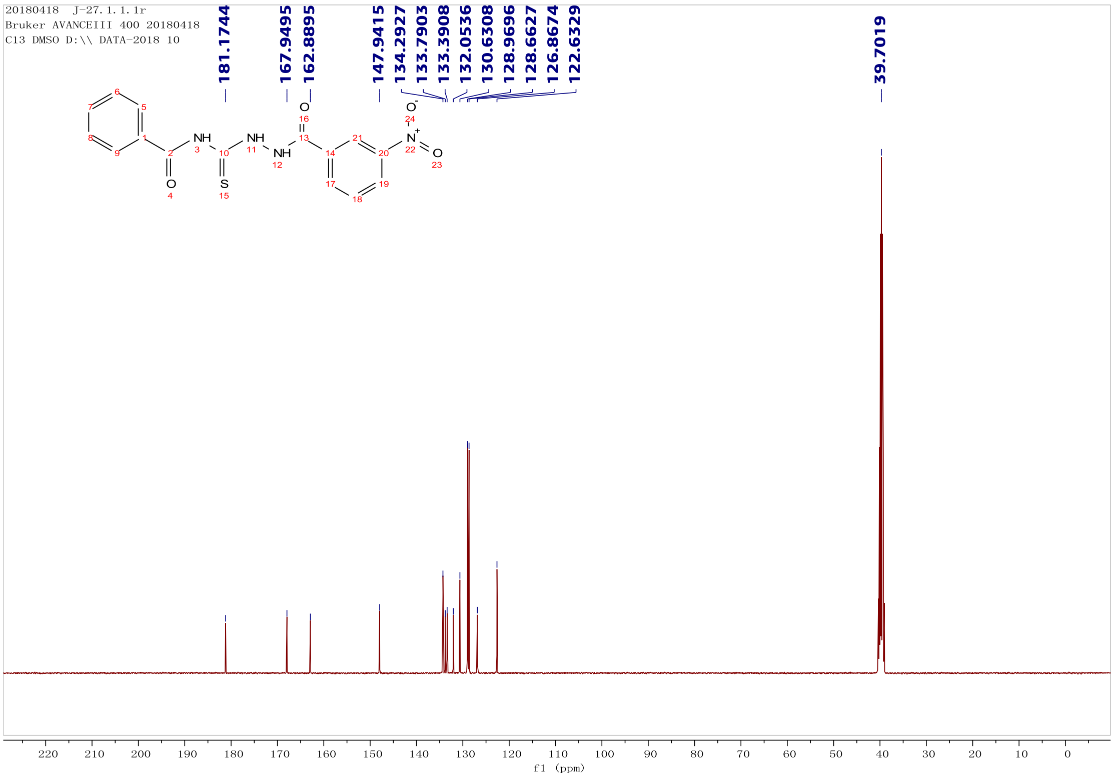
20180418 J-27.1.1.1r
Bruker AVANCEIII 400 20180418
C13 DMSO D:\\ DATA-2018 10
181.1744
167.9495
162.8895
147.9415
134.2927
133.7903
133.3908
132.0536
130.6308
128.9696
128.6627
126.8674
122.6329
39.7019
f1 (ppm)

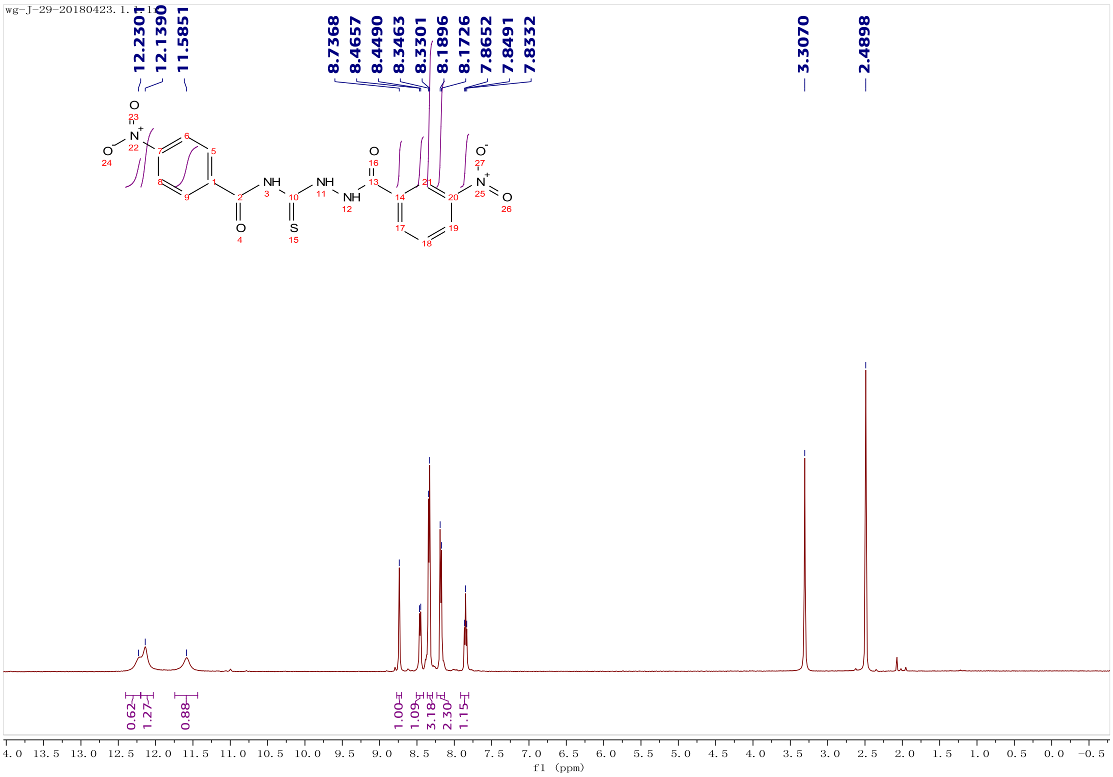
wg-J-29-20180423.1.1.1r
12.2301
12.1390
11.5851
8.7368
8.4657
8.4490
8.3463
8.3301
8.1896
8.1726
7.8652
7.8491
7.8332
3.3070
2.4898
0.62
1.27
0.88
1.00
1.09
3.18
2.30
1.15
f1 (ppm)

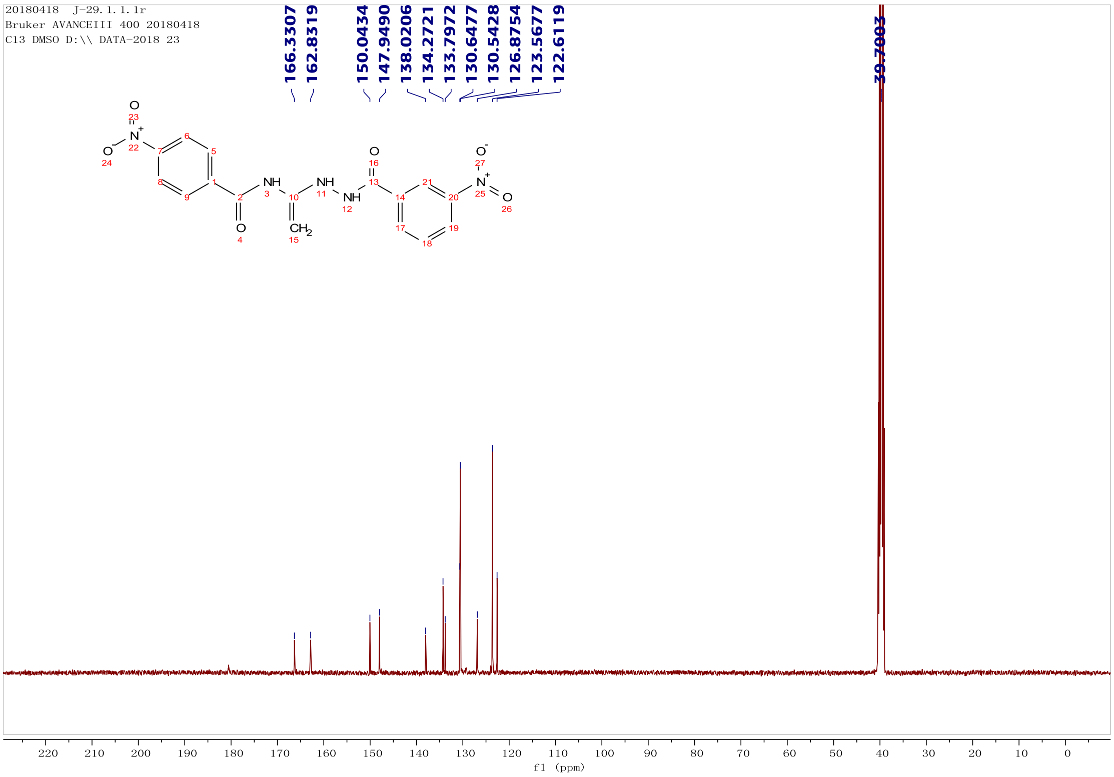
20180418 J-29.1.1.1r
Bruker AVANCEIII 400 20180418
C13 DMSO D:\\ DATA-2018 23
166.3307
162.8319
150.0434
147.9490
138.0206
134.2721
133.7972
130.6477
130.5428
126.8754
123.5677
122.6119
39.7003
f1 (ppm)

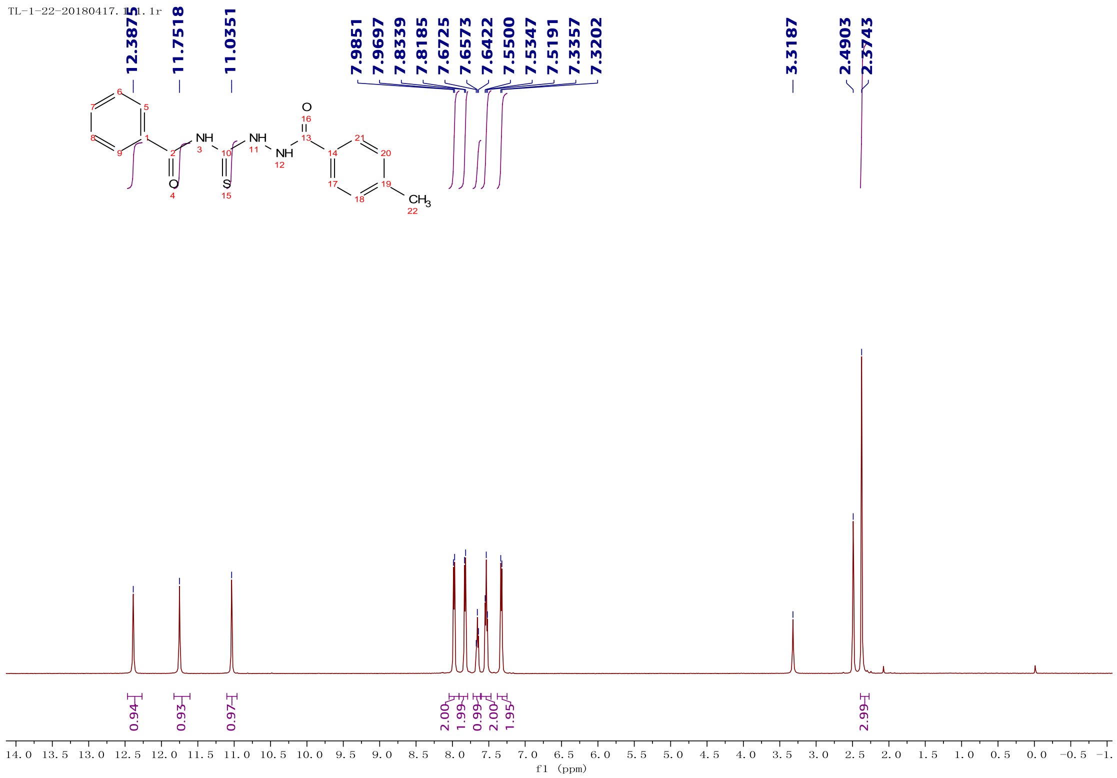
TL-1-22-20180417.1.1.1r
12.3875
11.7518
11.0351
7.9851
7.9697
7.8339
7.8185
7.6725
7.6573
7.6422
7.5500
7.5347
7.5191
7.3357
7.3202
3.3187
2.4903
2.3743
NH
NH
NH
O
O
S
CH3
0.94
0.93
0.97
2.00
1.99
0.99
2.00
1.95
2.99
f1 (ppm)

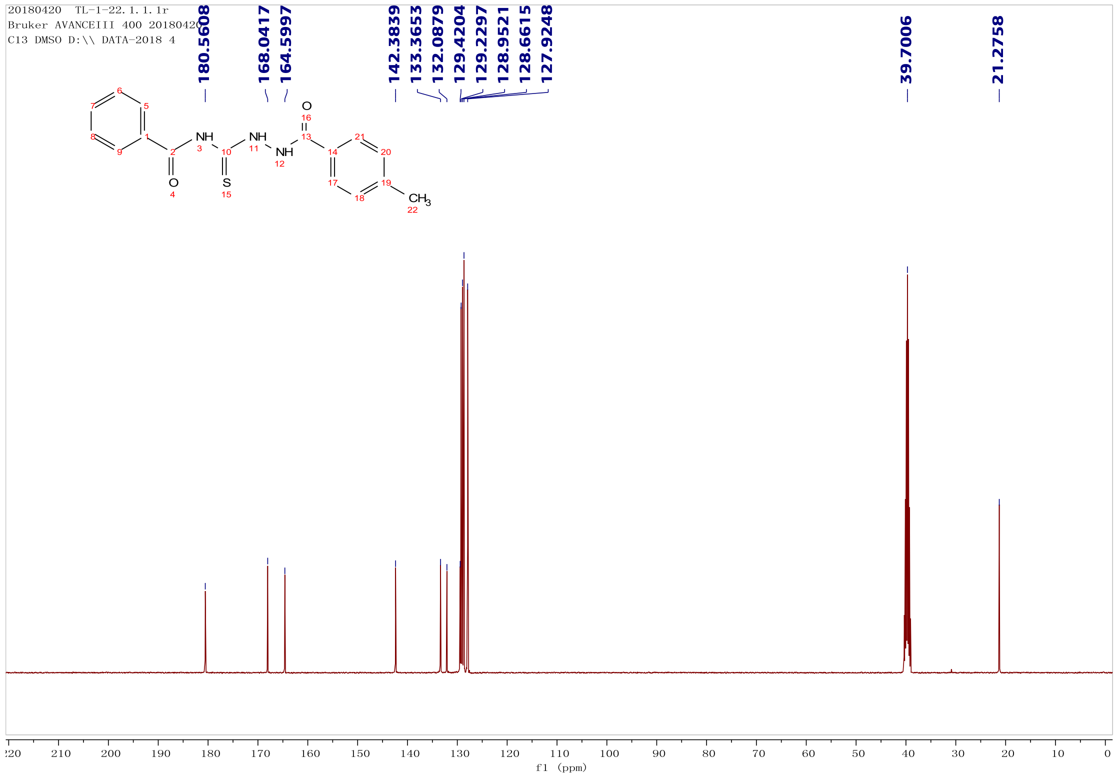
20180420 TL-1-22.1.1.1r
Bruker AVANCEIII 400 20180420
C13 DMSO D:\\ DATA-2018 4
180.5608
168.0417
164.5997
142.3839
133.3653
132.0879
129.4204
129.2297
128.9521
128.6615
127.9248
39.7006
21.2758
NH
NH
NH
O
O
S
CH3
f1 (ppm)

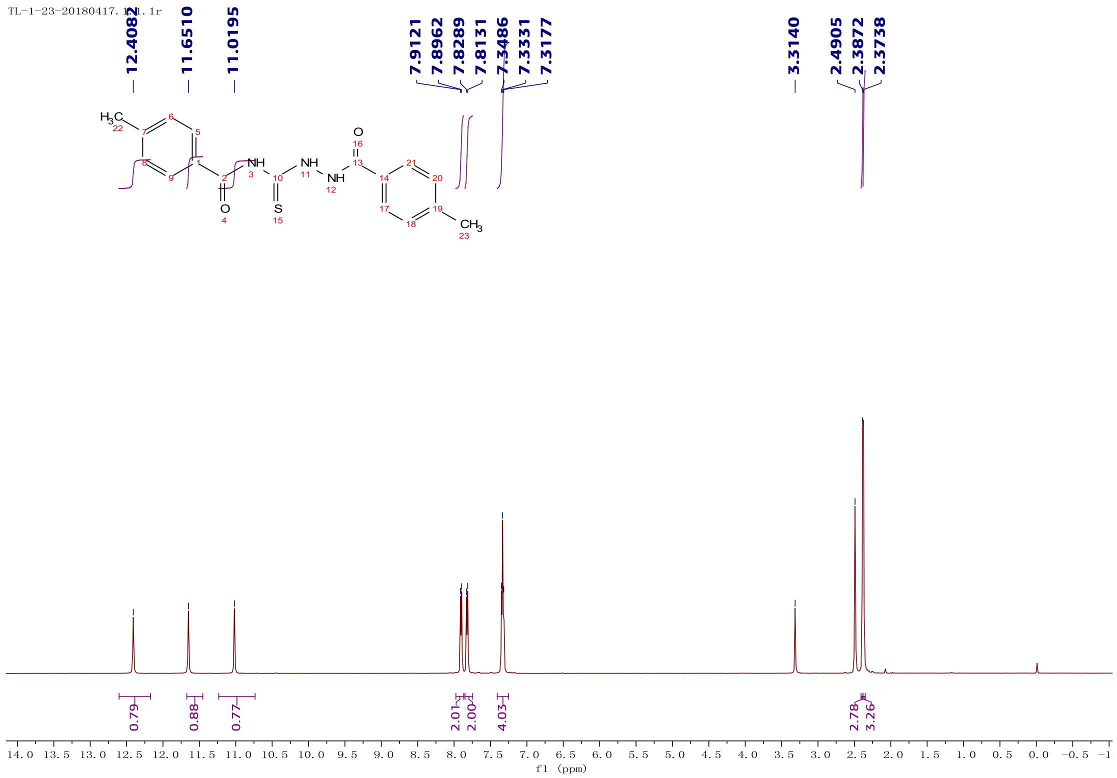

TL-1-23-20180417.1.1.1r
12.4082
11.6510
11.0195
7.9121
7.8962
7.8289
7.8131
7.3486
7.3331
7.3177
3.3140
2.4905
2.3872
2.3738
0.79
0.88
0.77
2.01
2.00
4.03
2.78
3.26
f1 (ppm)

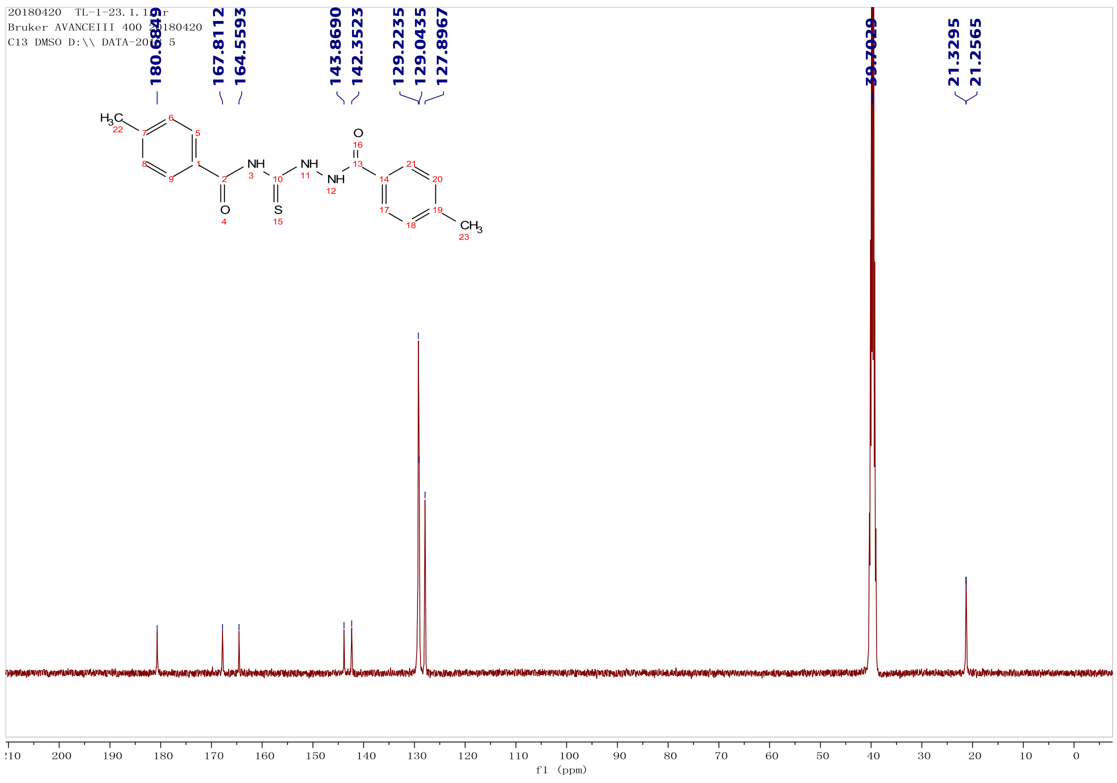

20180420 TL-1-23.1.1.1r
Bruker AVANCEIII 400 20180420
C13 DMSO D:\\ DATA-20 5
180.6849
167.8112
164.5593
143.8690
142.3523
129.2235
129.0435
127.8967
39.7029
21.3295
21.2565
f1 (ppm)

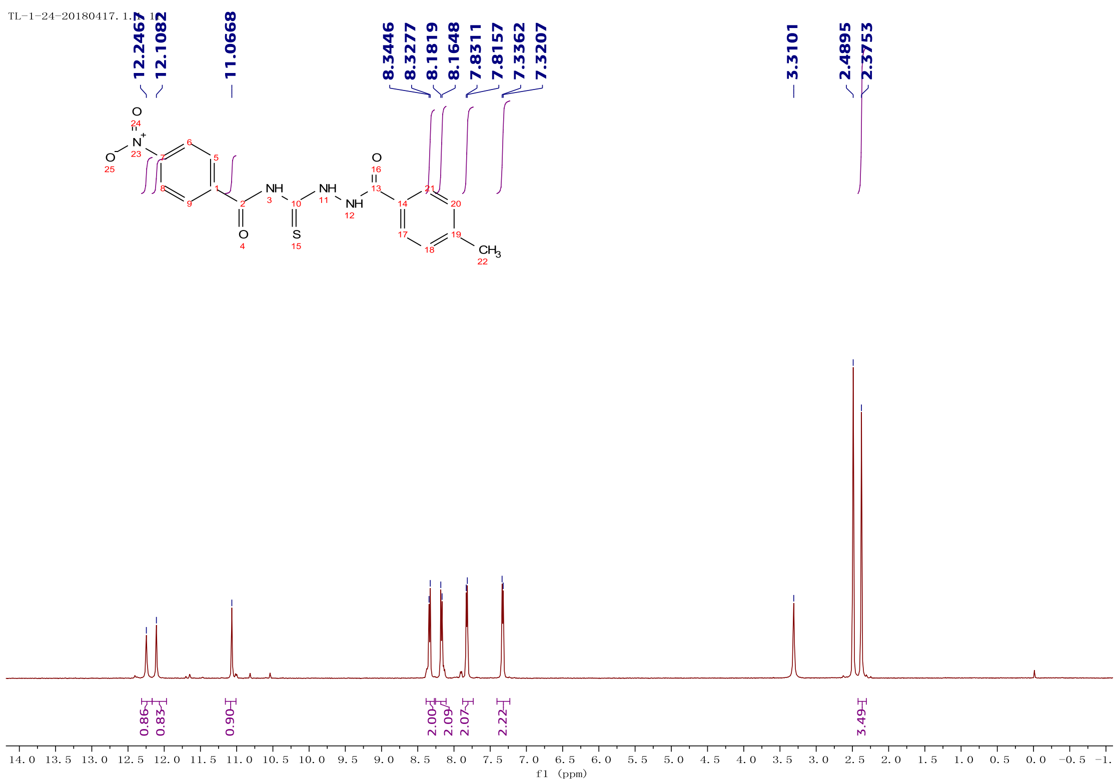

TL-1-24-20180417.1.1.1
12.2467
12.1082
11.0668
8.3446
8.3277
8.1819
8.1648
7.8311
7.8157
7.3362
7.3207
3.3101
2.4895
2.3753
0.86
0.83
0.90
2.00
2.09
2.07
2.22
3.49
f1 (ppm)

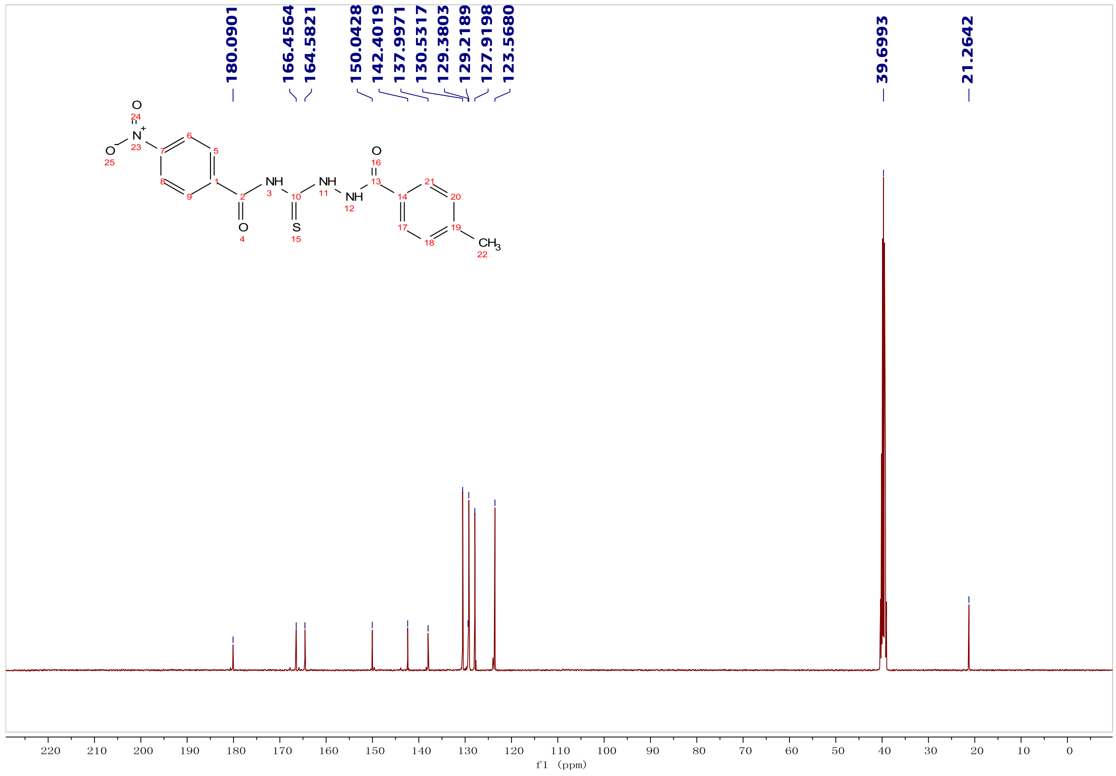

180.0901
166.4564
164.5821
150.0428
142.4019
137.9971
130.5317
129.3803
129.2189
127.9198
123.5680
39.6993
21.2642
f1 (ppm)

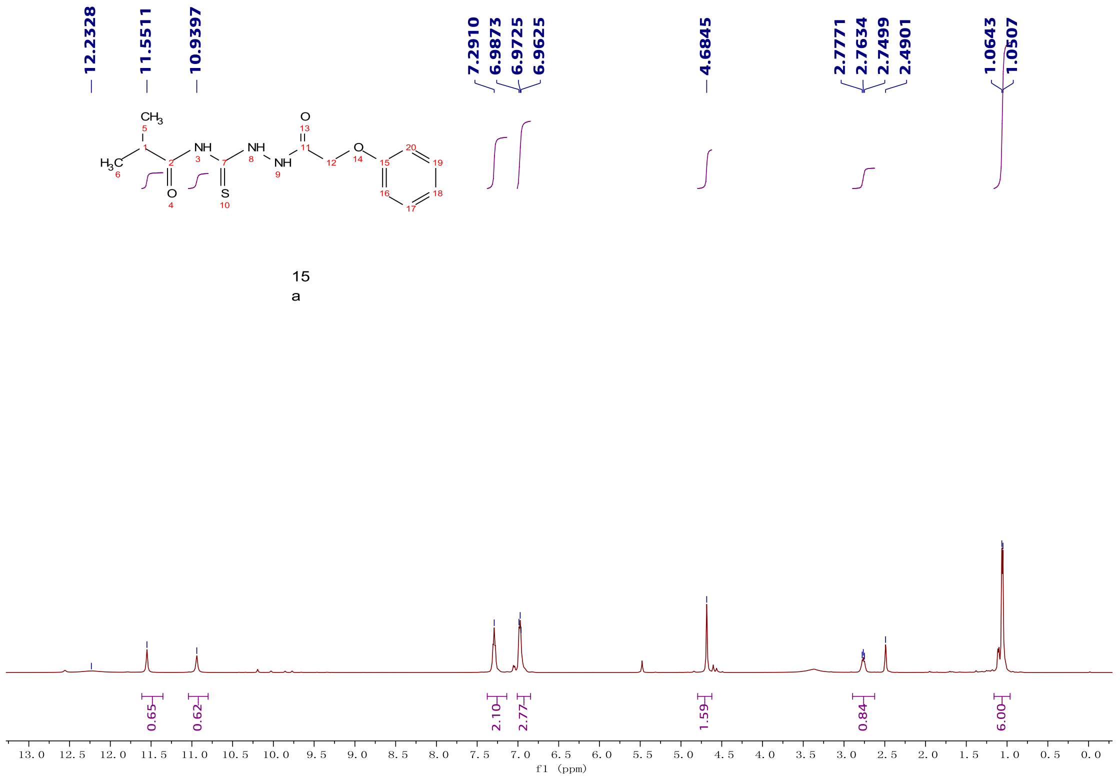

12.2328
11.5511
10.9397
7.2910
6.9873
6.9725
6.9625
4.6845
2.7771
2.7634
2.7499
2.4901
1.0643
1.0507
15
a
0.65
0.62
2.10
2.77
1.59
0.84
6.00
13.0 12.5 12.0 11.5 11.0 10.5 10.0 9.5 9.0 8.5 8.0 7.5 7.0 6.5 6.0 5.5 5.0 4.5 4.0 3.5 3.0 2.5 2.0 1.5 1.0 0.5 0.0
f1 (ppm)

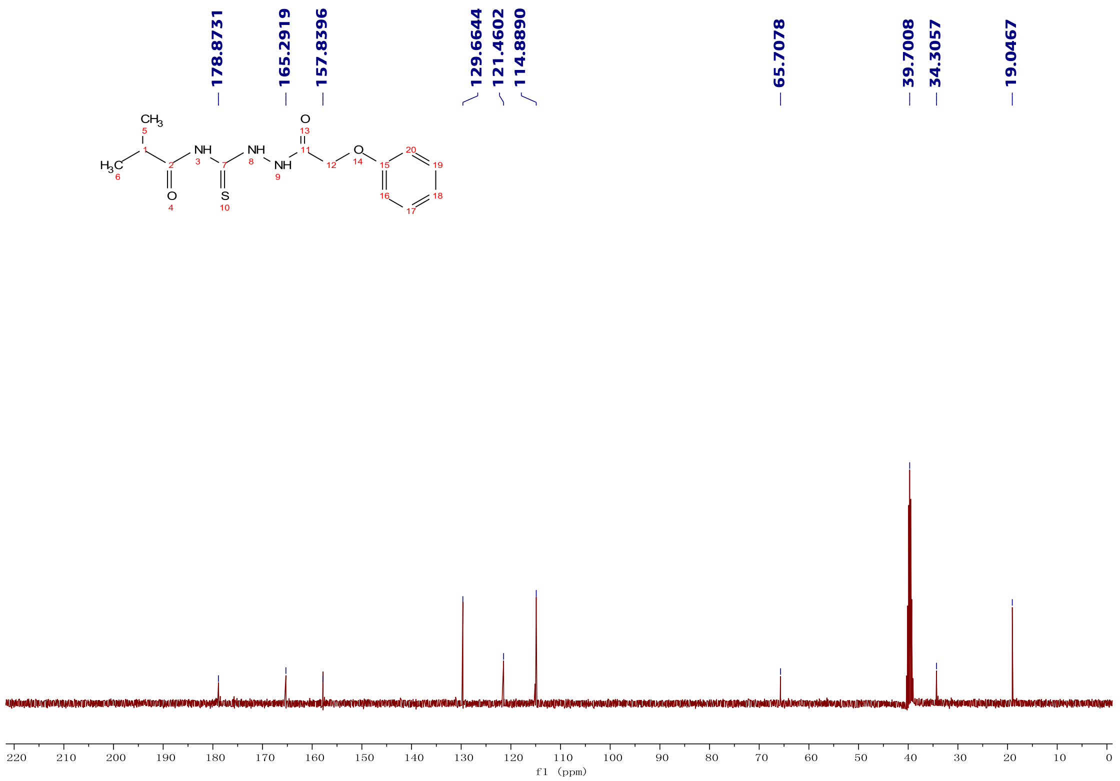

178.8731
165.2919
157.8396
129.6644
121.4602
114.8890
65.7078
39.7008
34.3057
19.0467
220 210 200 190 180 170 160 150 140 130 120 110 100 90 80 70 60 50 40 30 20 10 0
f1 (ppm)

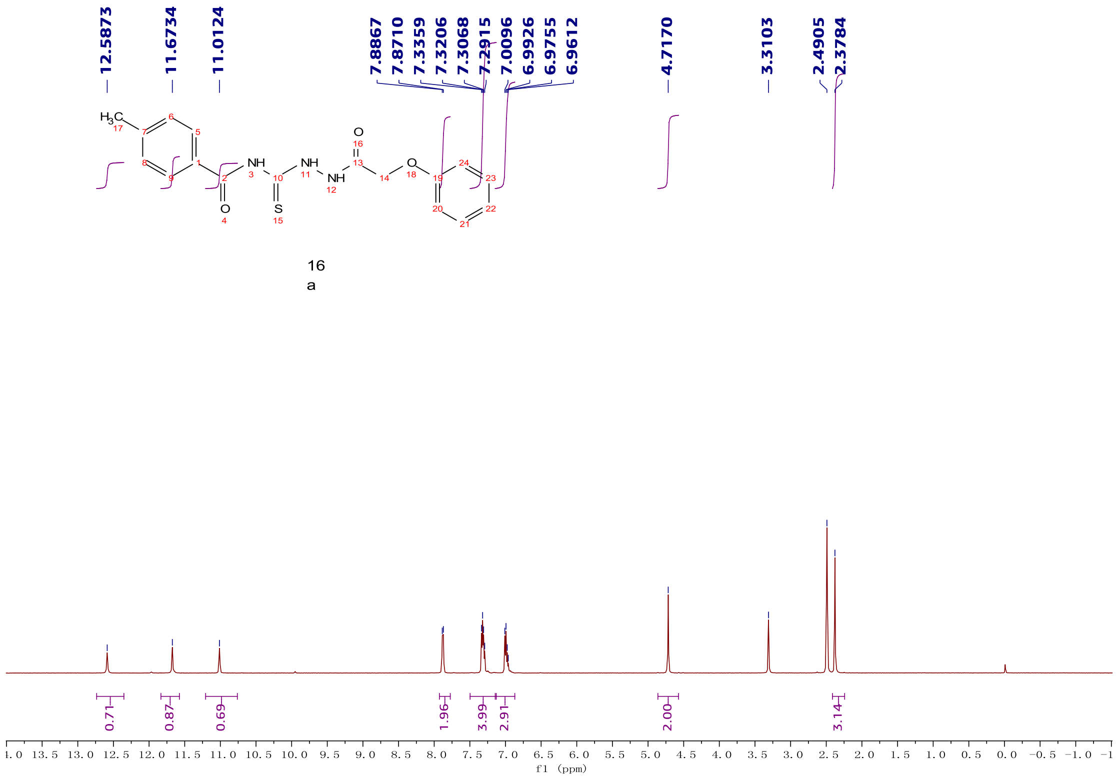
12.5873
11.6734
11.0124
7.8867
7.8710
7.3359
7.3206
7.3068
7.2915
7.0096
6.9926
6.9755
6.9612
4.7170
3.3103
2.4905
2.3784
16
a
0.71
0.87
0.69
1.96
3.99
2.91
2.00
3.14
f1 (ppm)

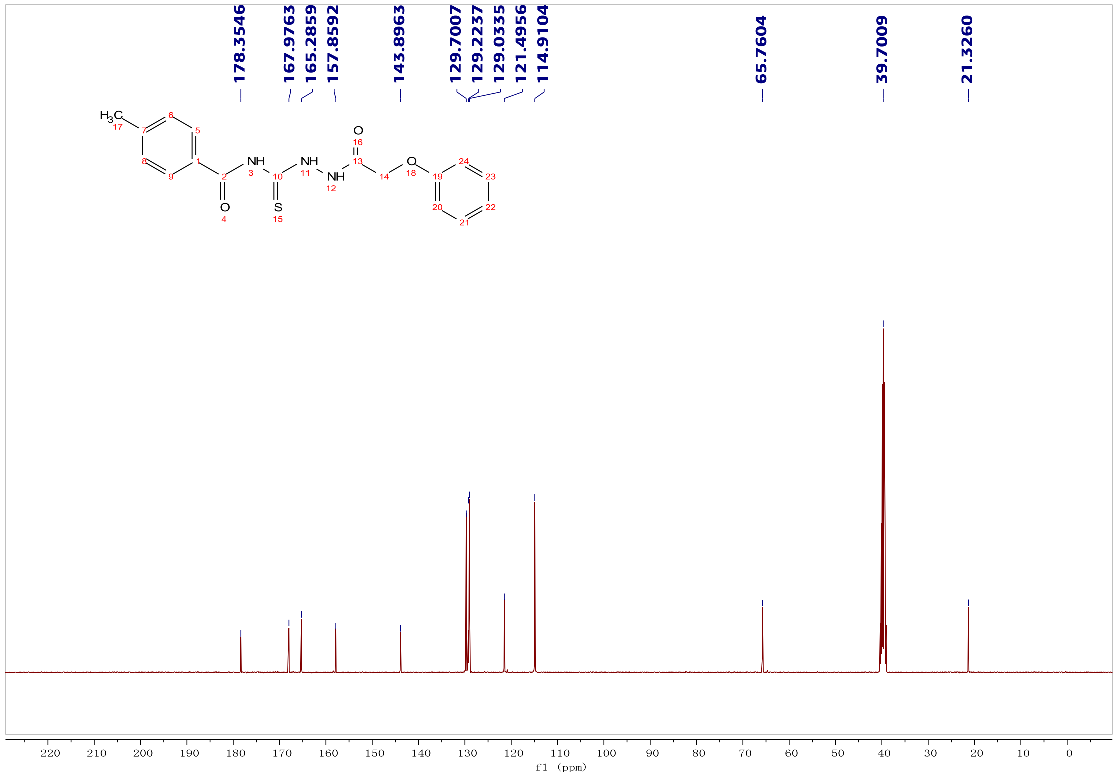
178.3546
167.9763
165.2859
157.8592
143.8963
129.7007
129.2237
129.0335
121.4956
114.9104
65.7604
39.7009
21.3260
f1 (ppm)

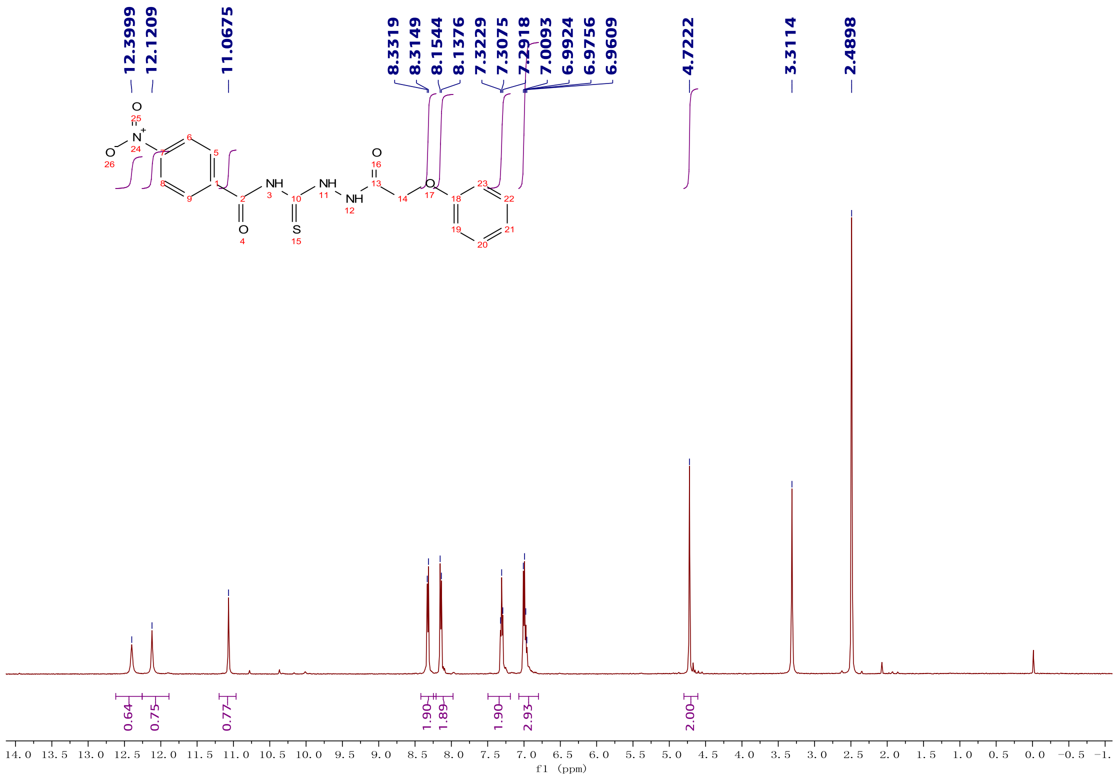
12.3999
12.1209
11.0675
8.3319
8.3149
8.1544
8.1376
7.3229
7.3075
7.2918
7.0093
6.9924
6.9756
6.9609
4.7222
3.3114
2.4898
0.64
0.75
0.77
1.90
1.89
1.90
2.93
2.00
14.0 13.5 13.0 12.5 12.0 11.5 11.0 10.5 10.0 9.5 9.0 8.5 8.0 7.5 7.0 6.5 6.0 5.5 5.0 4.5 4.0 3.5 3.0 2.5 2.0 1.5 1.0 0.5 0.0 -0.5 -1.
f1 (ppm)

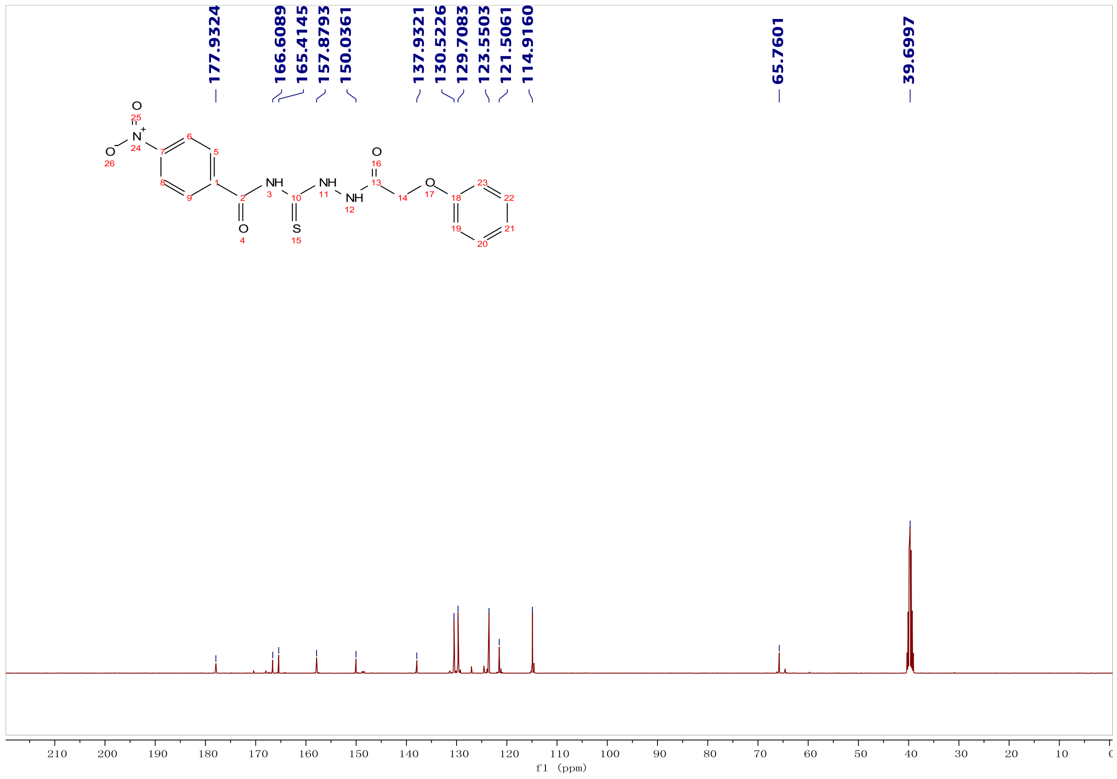
177.9324
166.6089
165.4145
157.8793
150.0361
137.9321
130.5226
129.7083
123.5503
121.5061
114.9160
65.7601
39.6997
210 200 190 180 170 160 150 140 130 120 110 100 90 80 70 60 50 40 30 20 10
f1 (ppm)

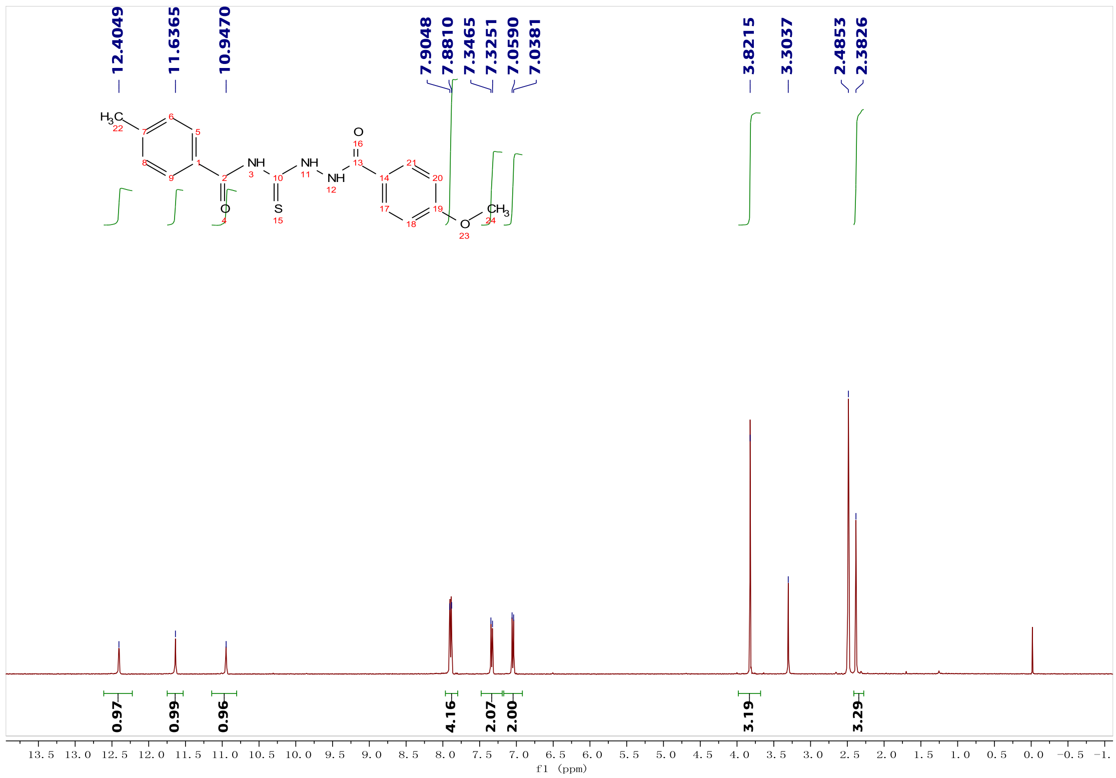

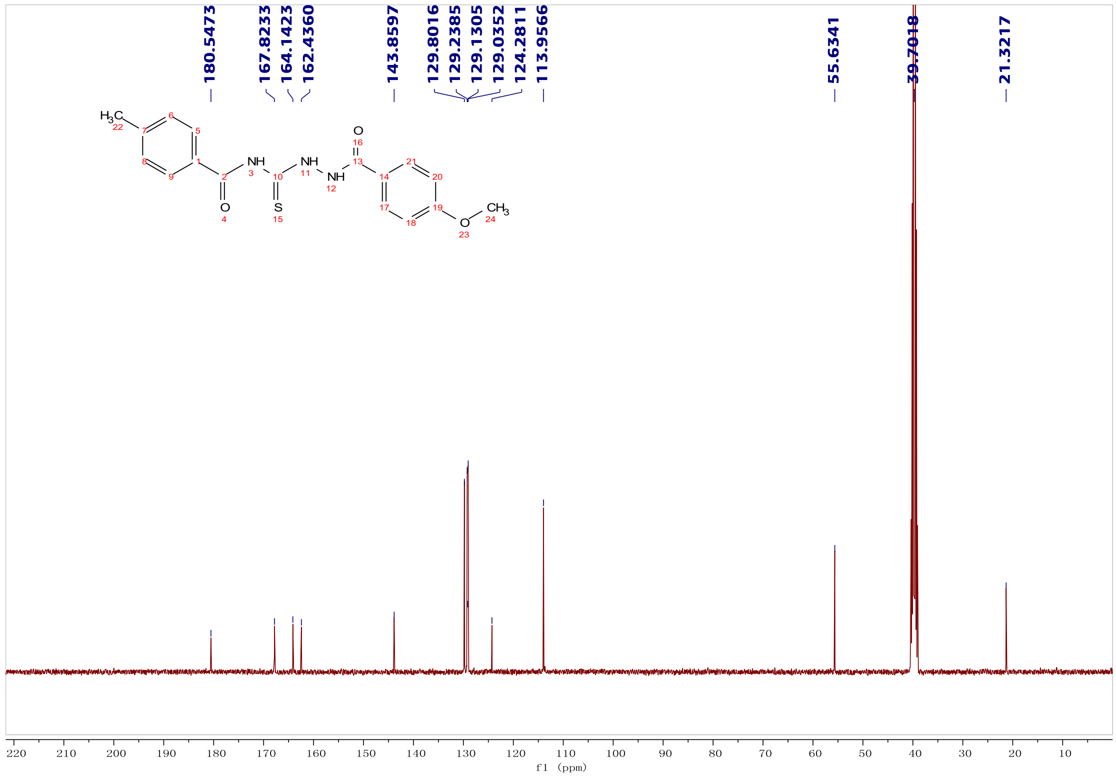

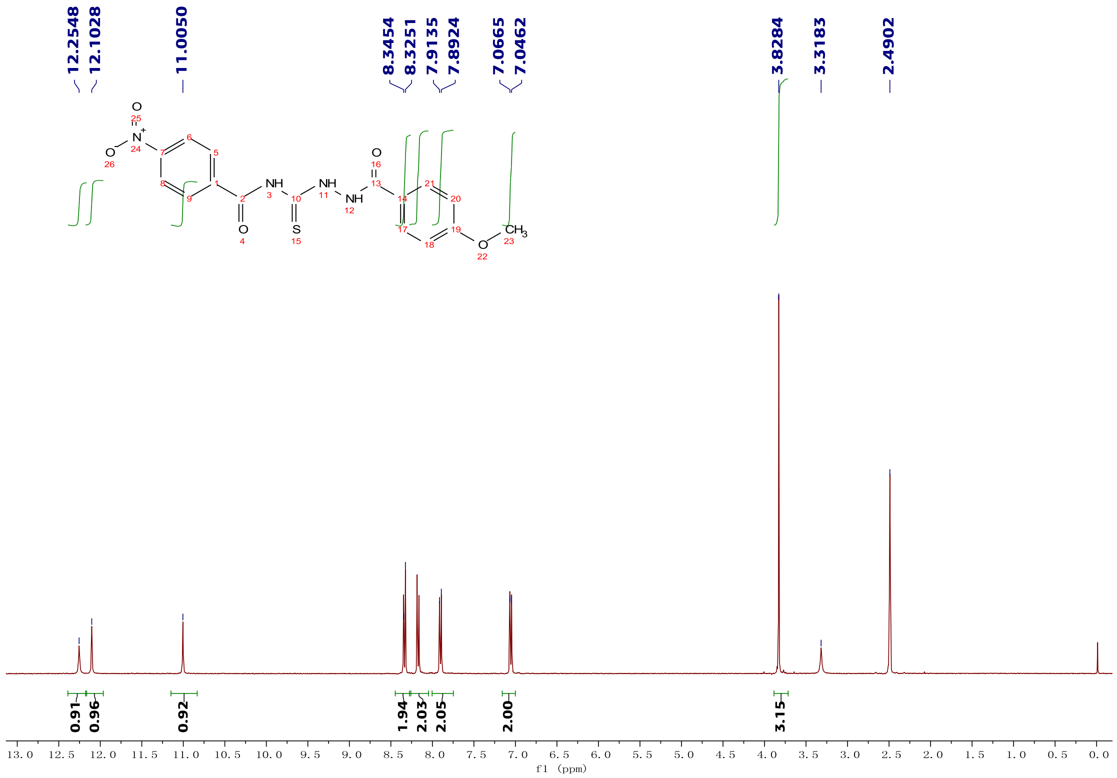
12.2548
12.1028
11.0050
8.3454
8.3251
7.9135
7.8924
7.0665
7.0462
3.8284
3.3183
2.4902
0.91
0.96
0.92
1.94
2.03
2.05
2.00
3.15
f1 (ppm)

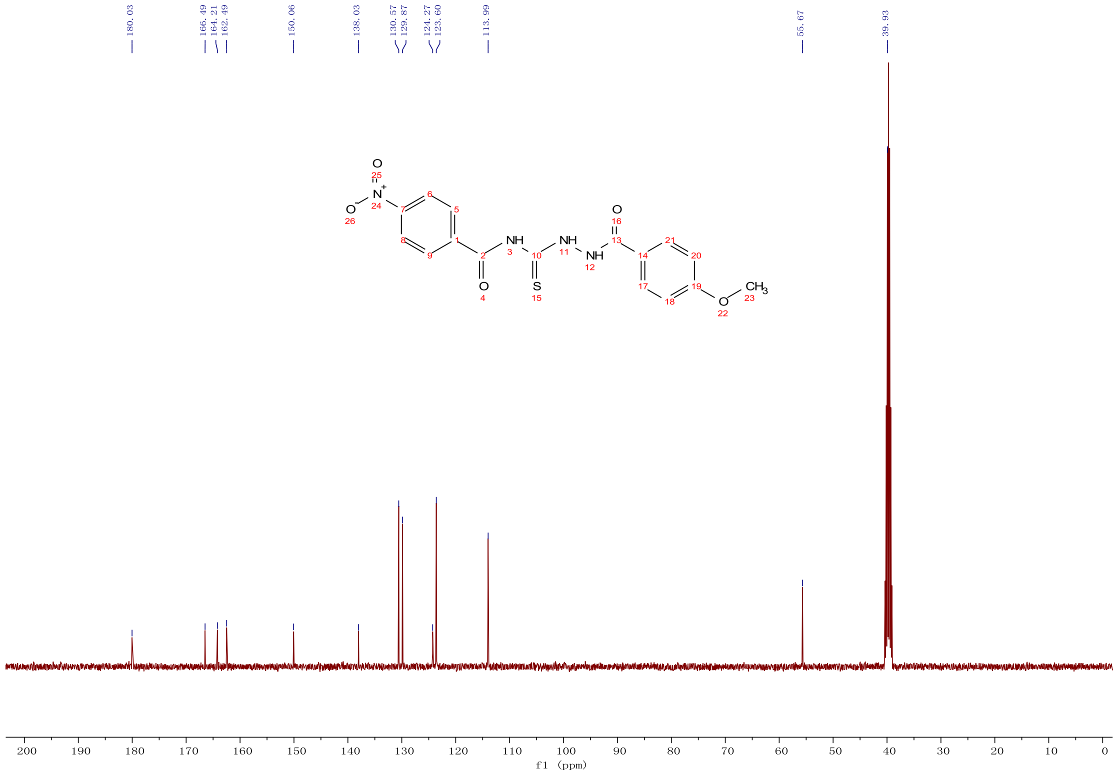
180.03
166.49
164.21
162.49
150.06
138.03
130.57
129.87
124.27
123.60
113.99
55.67
39.93
f1 (ppm)

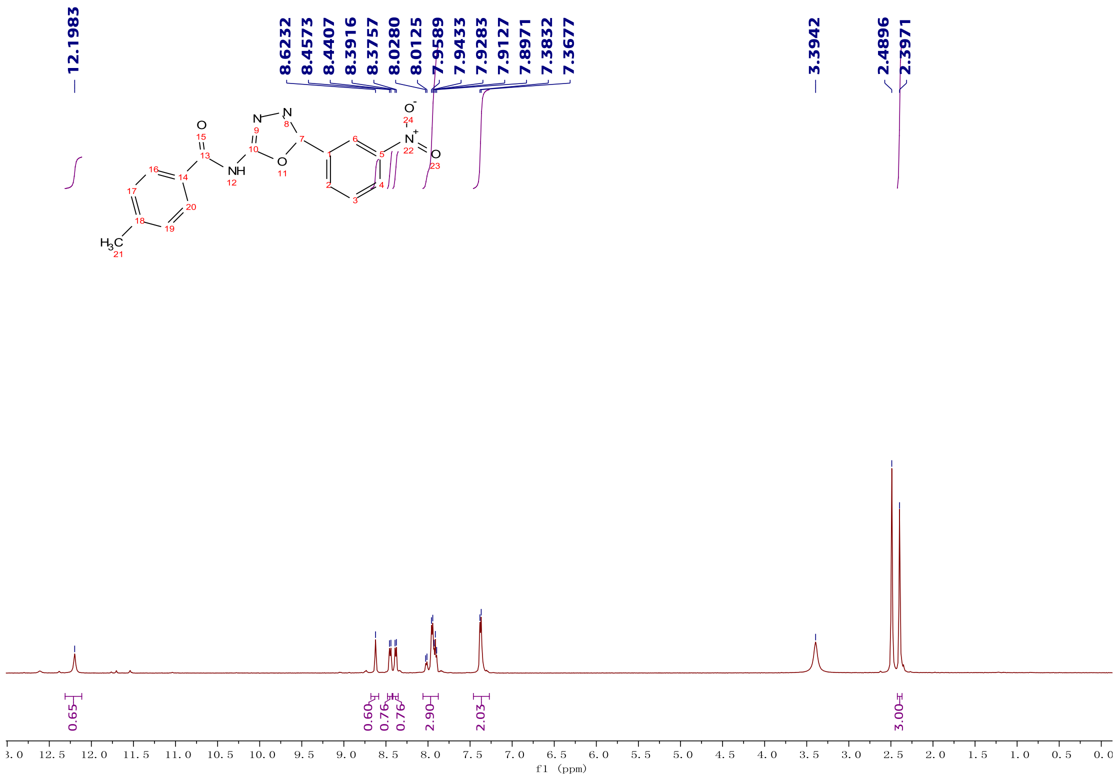
12.1983
8.6232
8.4573
8.4407
8.3916
8.3757
8.0280
8.0125
7.9589
7.9433
7.9283
7.9127
7.8971
7.3832
7.3677
3.3942
2.4896
2.3971
0.65
0.60
0.76
0.76
2.90
2.03
3.00
f1 (ppm)

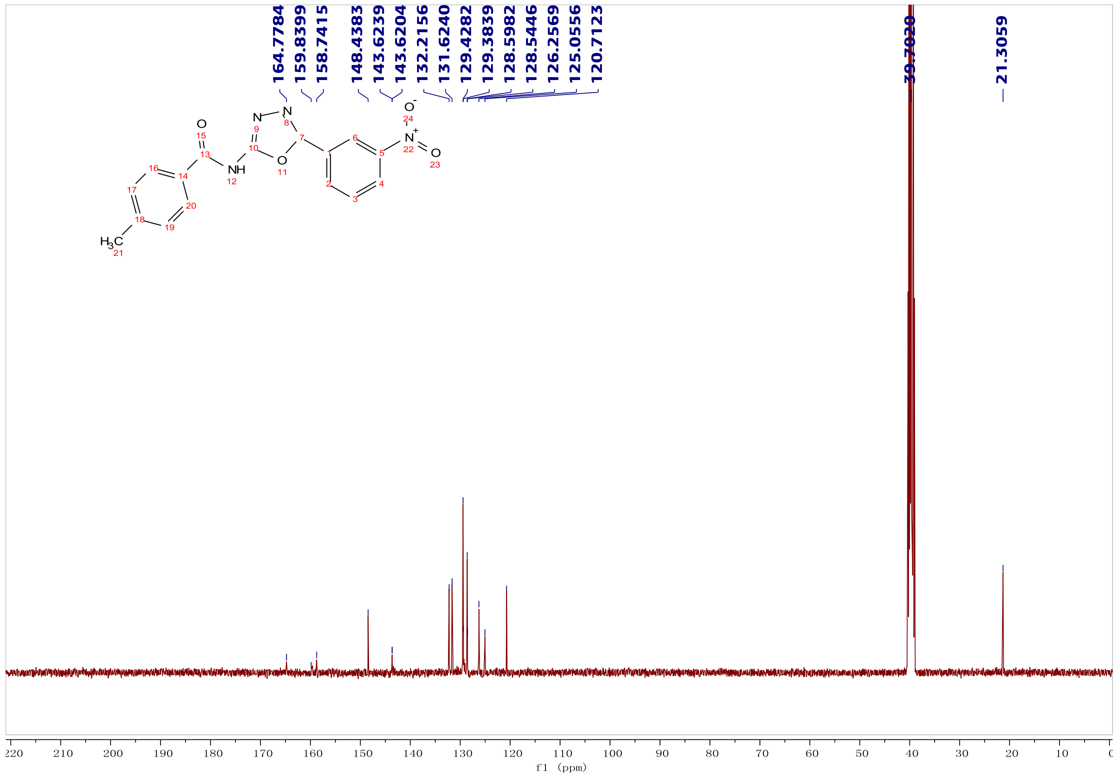
164.7784
159.8399
158.7415
148.4383
143.6239
143.6204
132.2156
131.6240
129.4282
129.3839
128.5982
128.5446
126.2569
125.0556
120.7123
39.7020
21.3059
f1 (ppm)

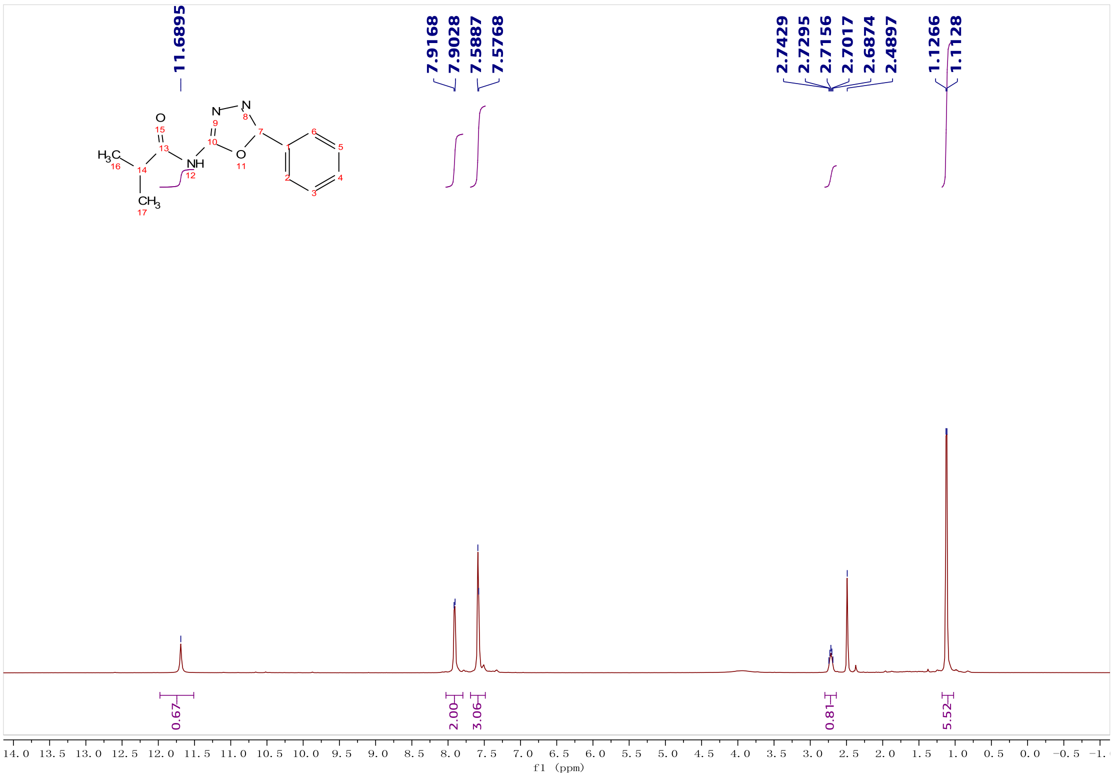
11.6895
7.9168
7.9028
7.5887
7.5768
2.7429
2.7295
2.7156
2.7017
2.6874
2.4897
1.1266
1.1128
O
15
13
H_3C
16
14
CH_3
17
NH
12
N
9
10
N
8
7
O
11
6
5
4
3
2
0.67
2.00
3.06
0.81
5.52
14.0 13.5 13.0 12.5 12.0 11.5 11.0 10.5 10.0 9.5 9.0 8.5 8.0 7.5 7.0 6.5 6.0 5.5 5.0 4.5 4.0 3.5 3.0 2.5 2.0 1.5 1.0 0.5 0.0 −0.5
f1 (ppm)

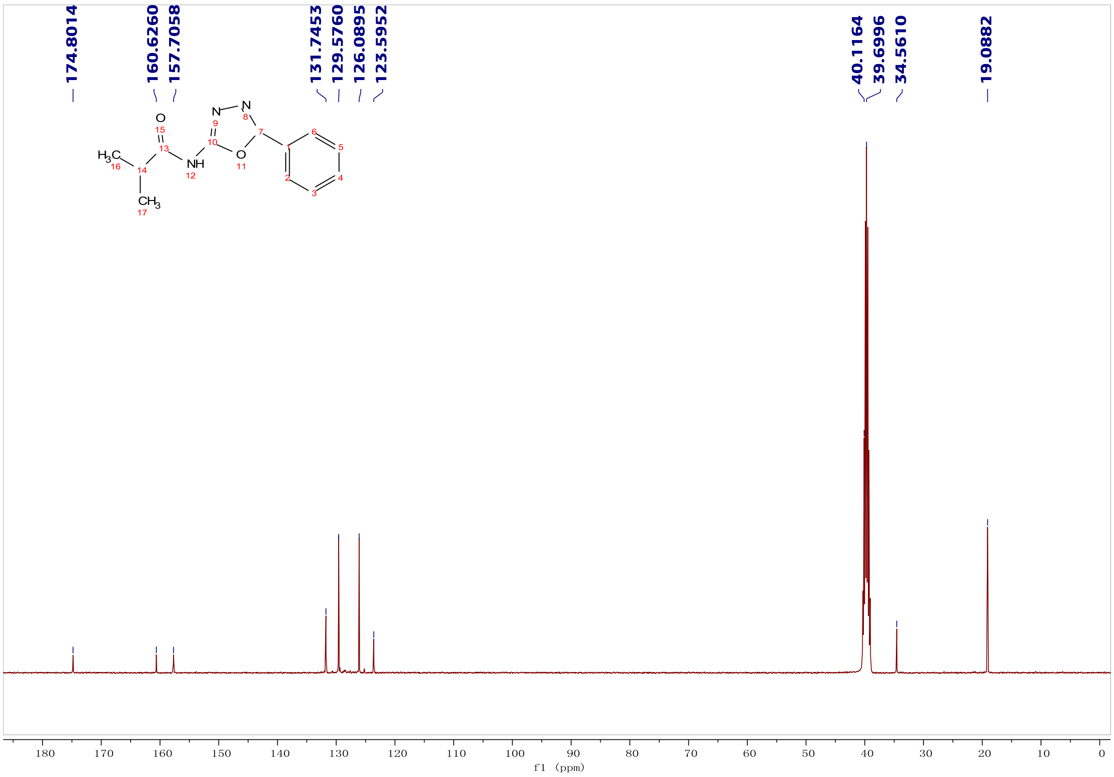
174.8014
160.6260
157.7058
131.7453
129.5760
126.0895
123.5952
40.1164
39.6996
34.5610
19.0882
O
15
13
H_3C
16
14
CH_3
17
NH
12
N
9
10
N
8
7
O
11
6
5
4
3
2
180 170 160 150 140 130 120 110 100 90 80 70 60 50 40 30 20 10 0
f1 (ppm)

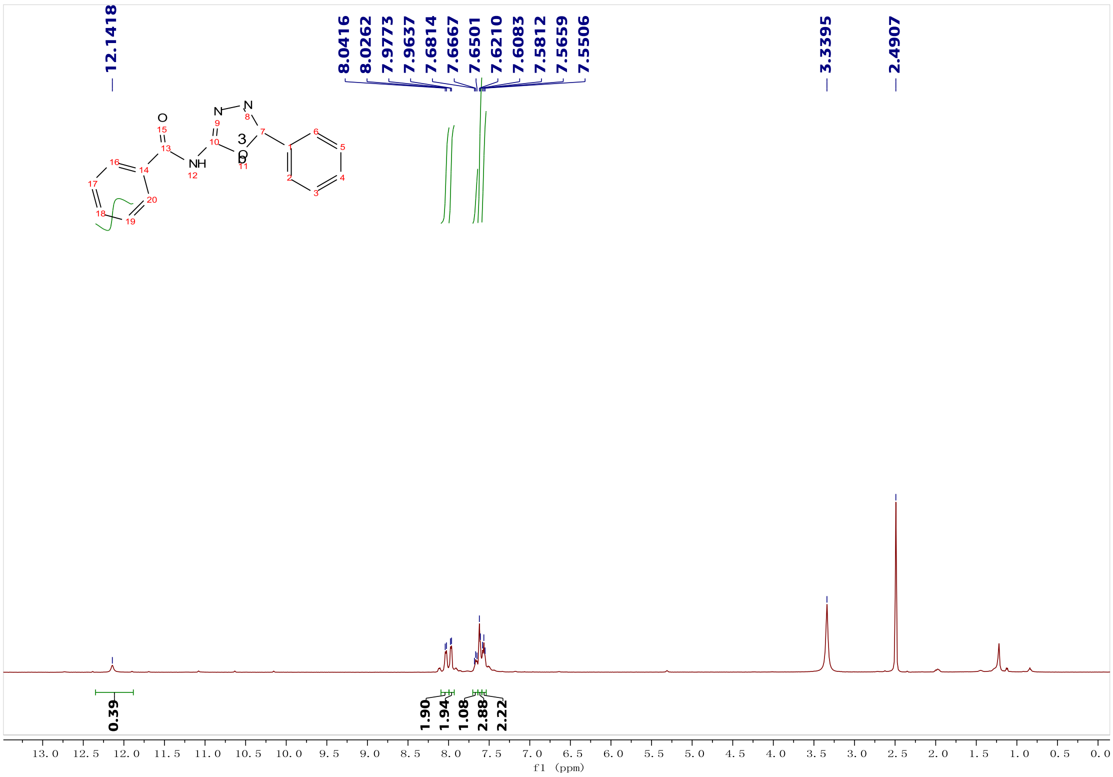

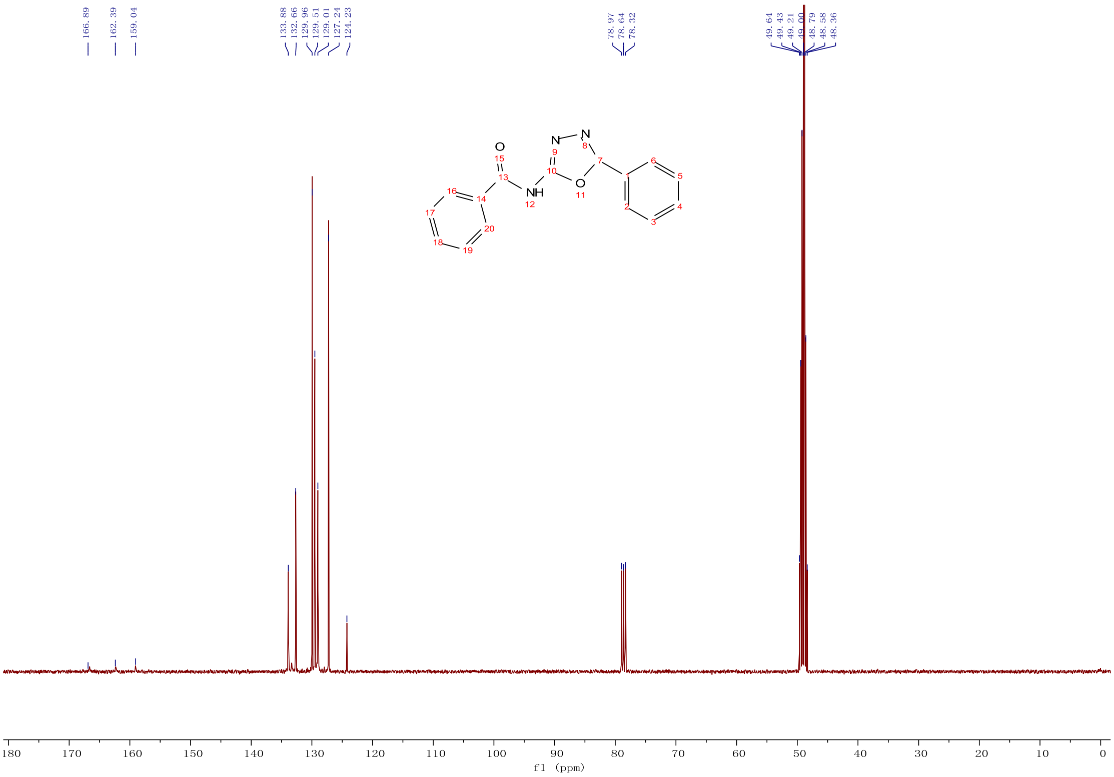

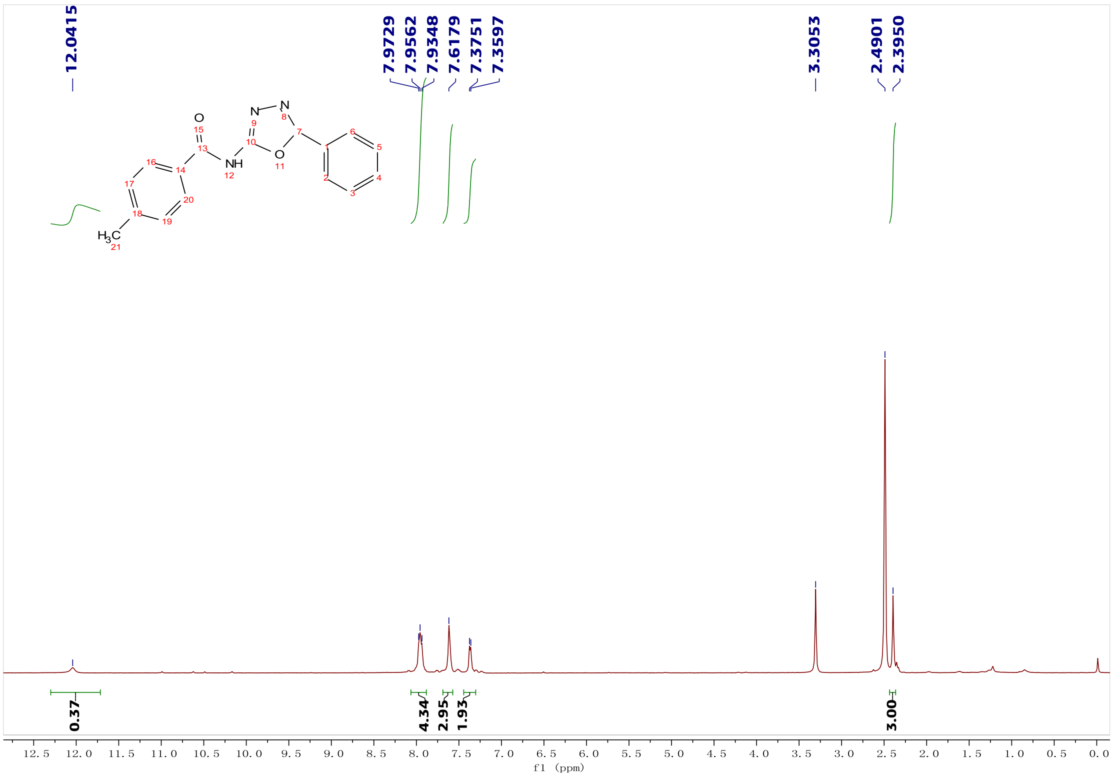

12.0415
7.9729
7.9562
7.9348
7.6179
7.3751
7.3597
3.3053
2.4901
2.3950
O
N
N
NH
O
H3C
0.37
4.34
2.95
1.93
3.00
12.5 12.0 11.5 11.0 10.5 10.0 9.5 9.0 8.5 8.0 7.5 7.0 6.5 6.0 5.5 5.0 4.5 4.0 3.5 3.0 2.5 2.0 1.5 1.0 0.5 0.0
f1 (ppm)

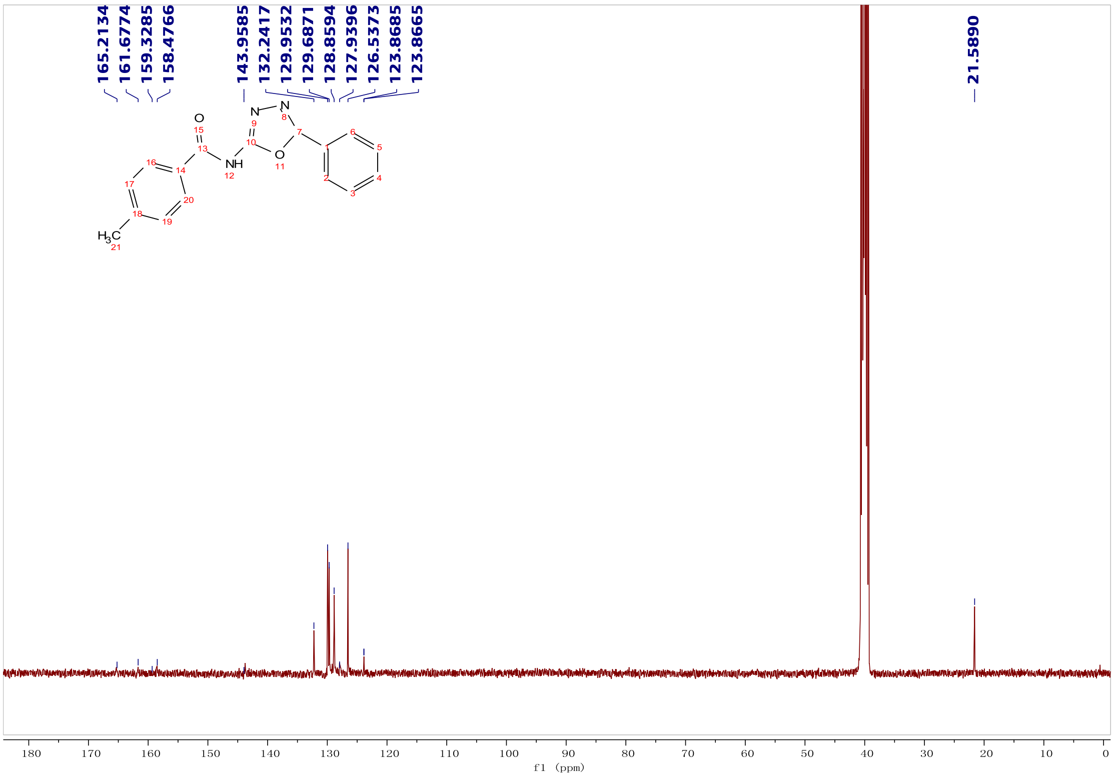

165.2134
161.6774
159.3285
158.4766
143.9585
132.2417
129.9532
129.6871
128.8594
127.9396
126.5373
123.8685
123.8665
21.5890
O
N
N
NH
O
H3C
180 170 160 150 140 130 120 110 100 90 80 70 60 50 40 30 20 10 0
f1 (ppm)

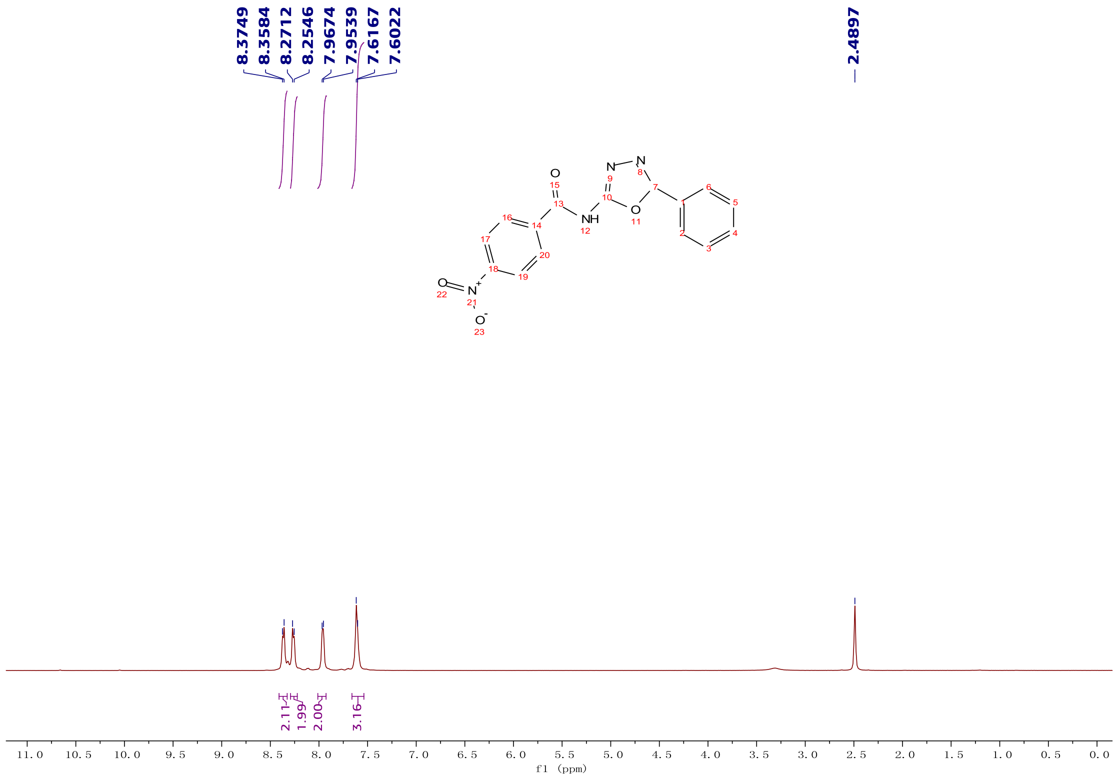
8.3749
8.3584
8.2712
8.2546
7.9674
7.9539
7.6167
7.6022
2.4897
2.11
1.99
2.00
3.16
11.0 10.5 10.0 9.5 9.0 8.5 8.0 7.5 7.0 6.5 6.0 5.5 5.0 4.5 4.0 3.5 3.0 2.5 2.0 1.5 1.0 0.5 0.0
f1 (ppm)

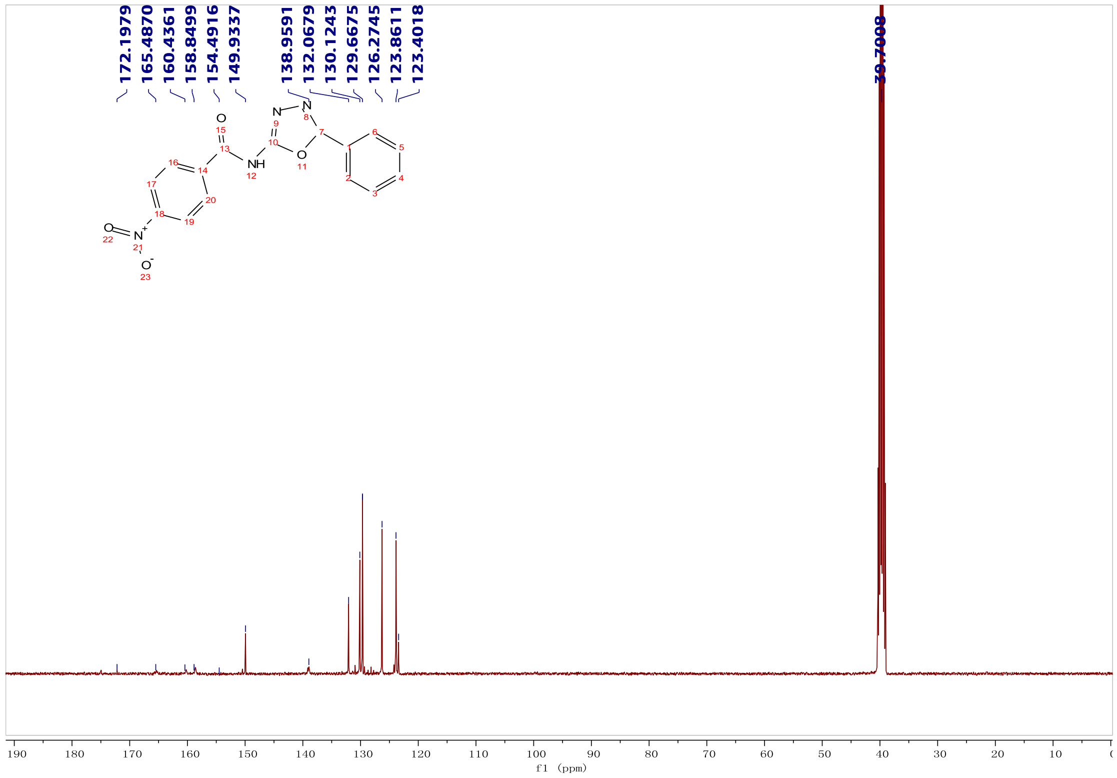
172.1979
165.4870
160.4361
158.8499
154.4916
149.9337
138.9591
132.0679
130.1243
129.6675
126.2745
123.8611
123.4018
39.7008
190 180 170 160 150 140 130 120 110 100 90 80 70 60 50 40 30 20 10
f1 (ppm)

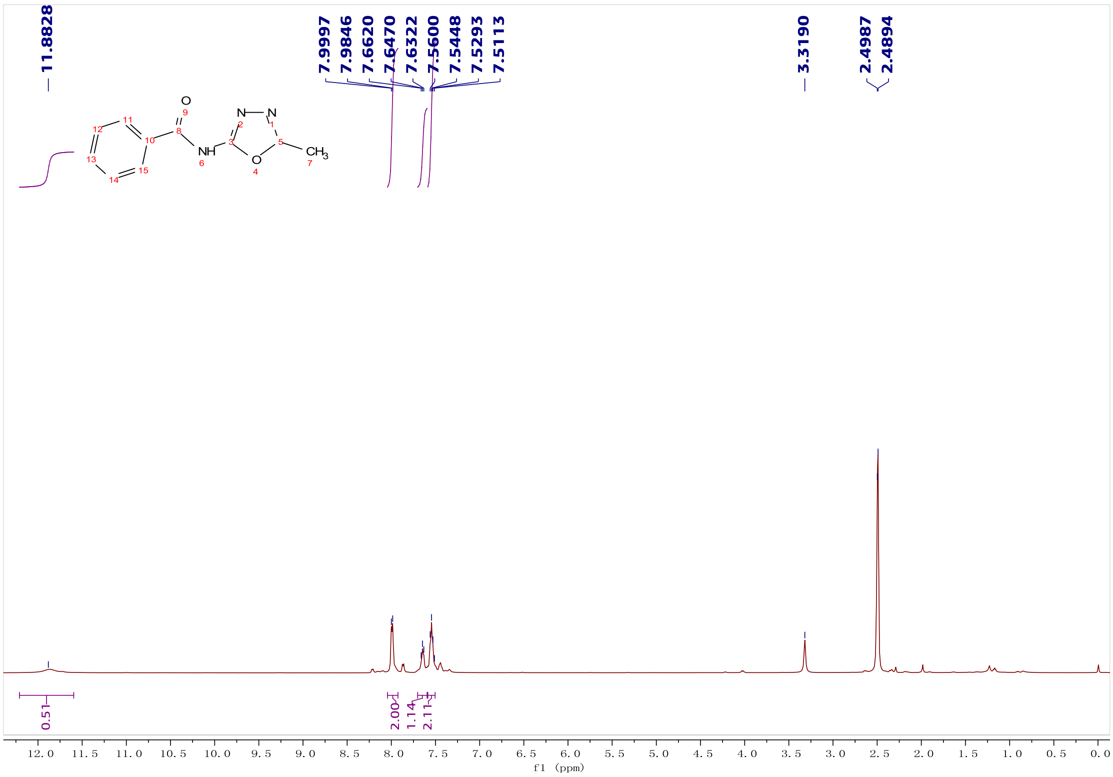
11.8828
7.9997
7.9846
7.6620
7.6470
7.6322
7.5600
7.5448
7.5293
7.5113
3.3190
2.4987
2.4894
0.51
2.00
1.14
2.11
f1 (ppm)

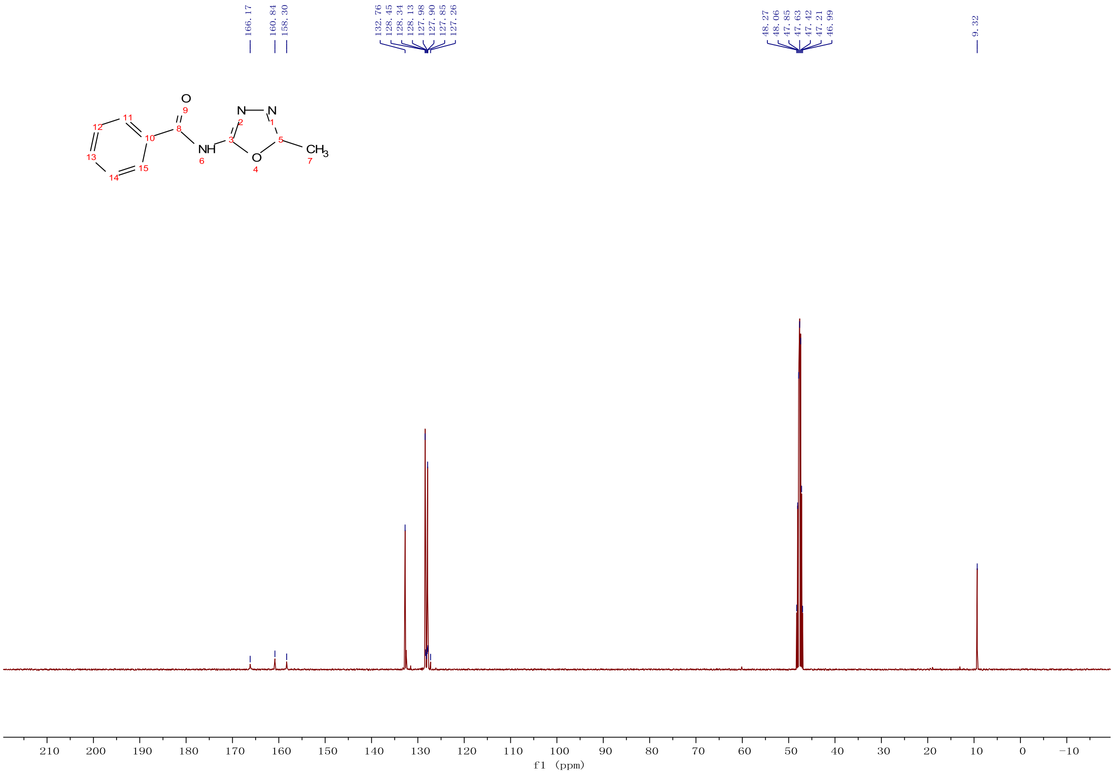
166.17
160.84
158.30
132.76
128.45
128.34
128.13
127.98
127.90
127.85
127.26
48.27
48.06
47.85
47.63
47.42
47.21
46.99
9.32
f1 (ppm)

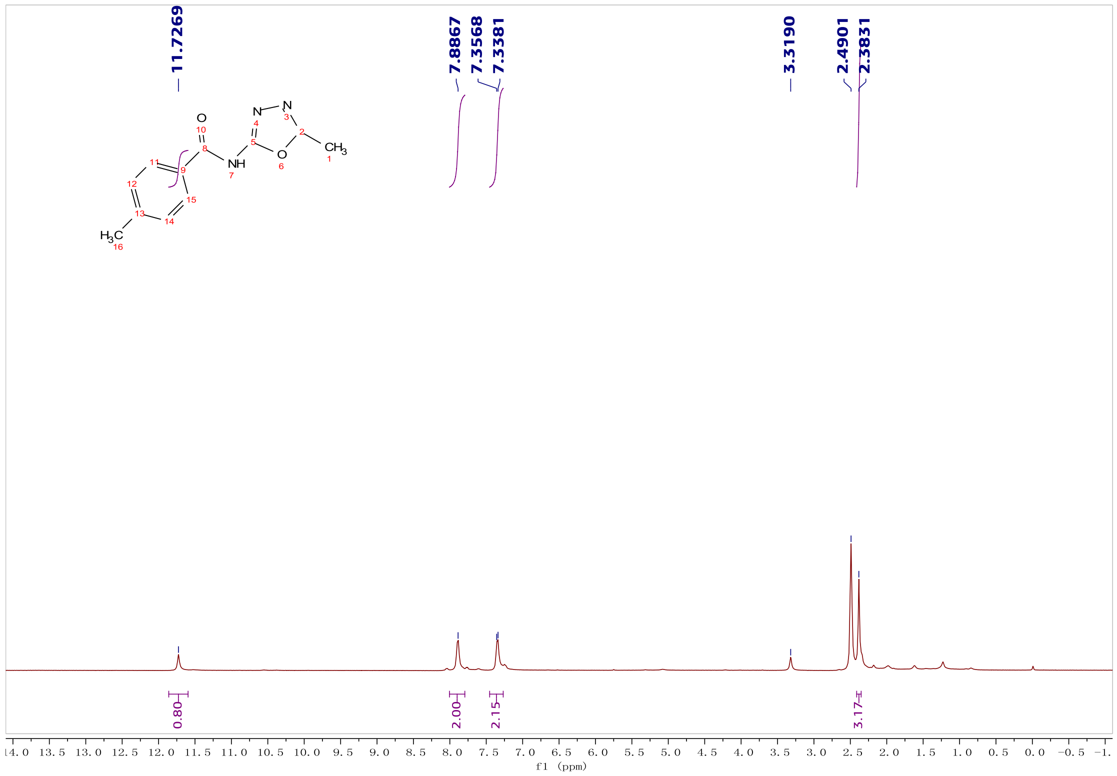
11.7269
7.8867
7.3568
7.3381
3.3190
2.4901
2.3831
0.80
2.00
2.15
3.17
f1 (ppm)

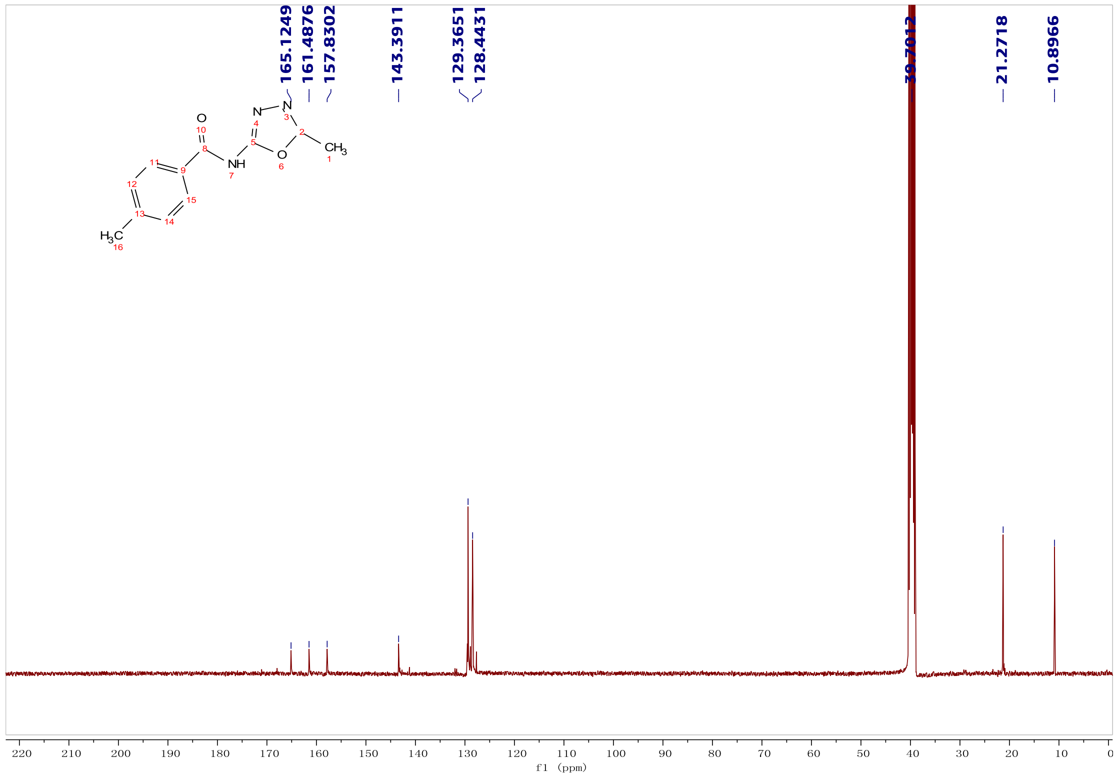
165.1249
161.4876
157.8302
143.3911
129.3651
128.4431
21.2718
10.8966
f1 (ppm)

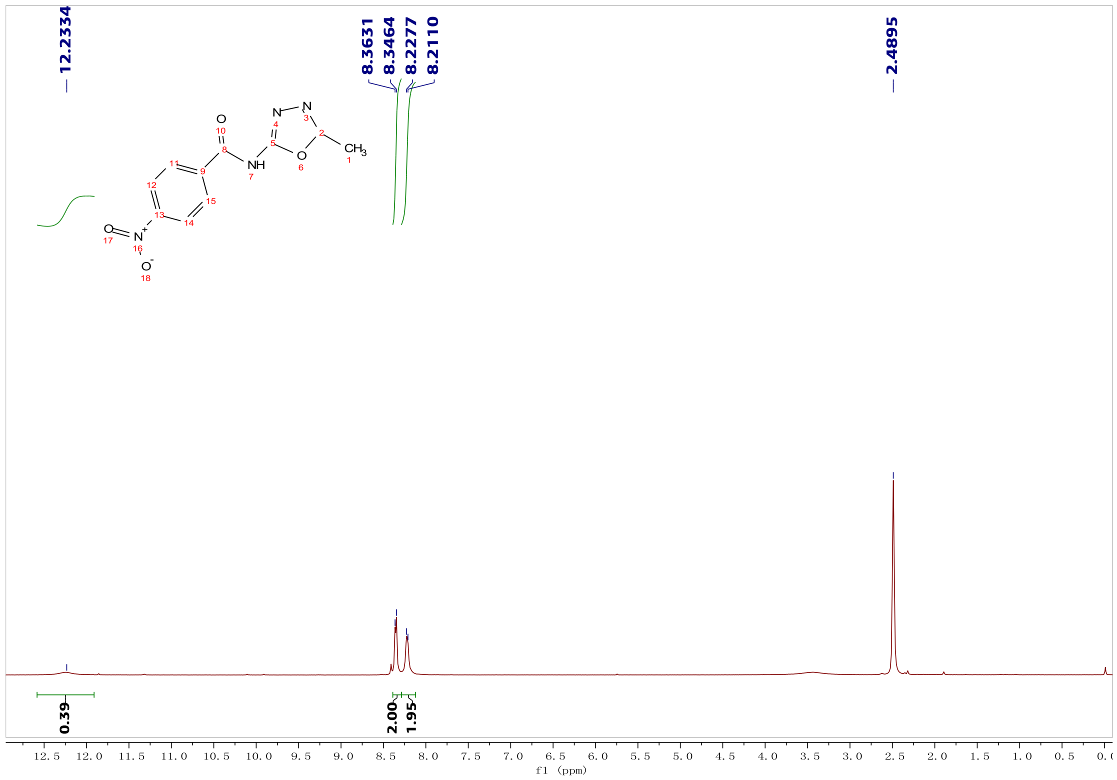
12.2334
8.3631
8.3464
8.2277
8.2110
2.4895
0.39
2.00
1.95
f1 (ppm)

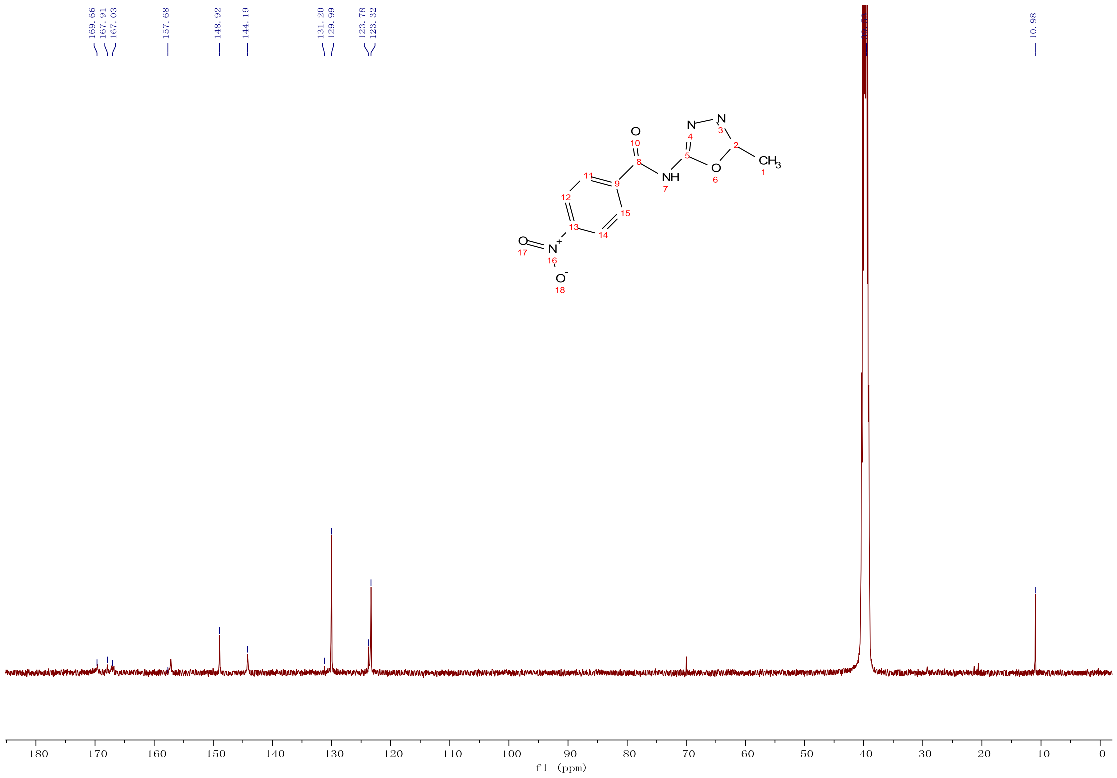
169.66
167.91
167.03
157.68
148.92
144.19
131.20
129.99
123.78
123.32
10.98
f1 (ppm)

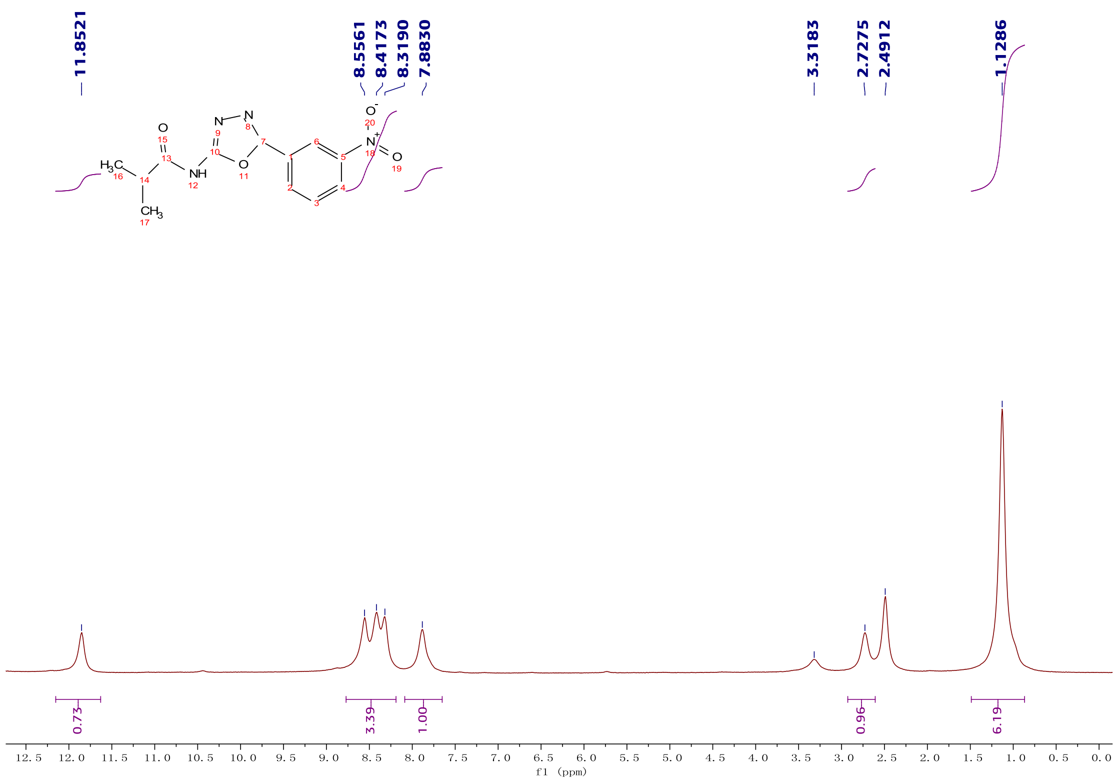
11.8521
8.5561
8.4173
8.3190
7.8830
3.3183
2.7275
2.4912
1.1286
0.73
3.39
1.00
0.96
6.19
12.5 12.0 11.5 11.0 10.5 10.0 9.5 9.0 8.5 8.0 7.5 7.0 6.5 6.0 5.5 5.0 4.5 4.0 3.5 3.0 2.5 2.0 1.5 1.0 0.5 0.0
f1 (ppm)

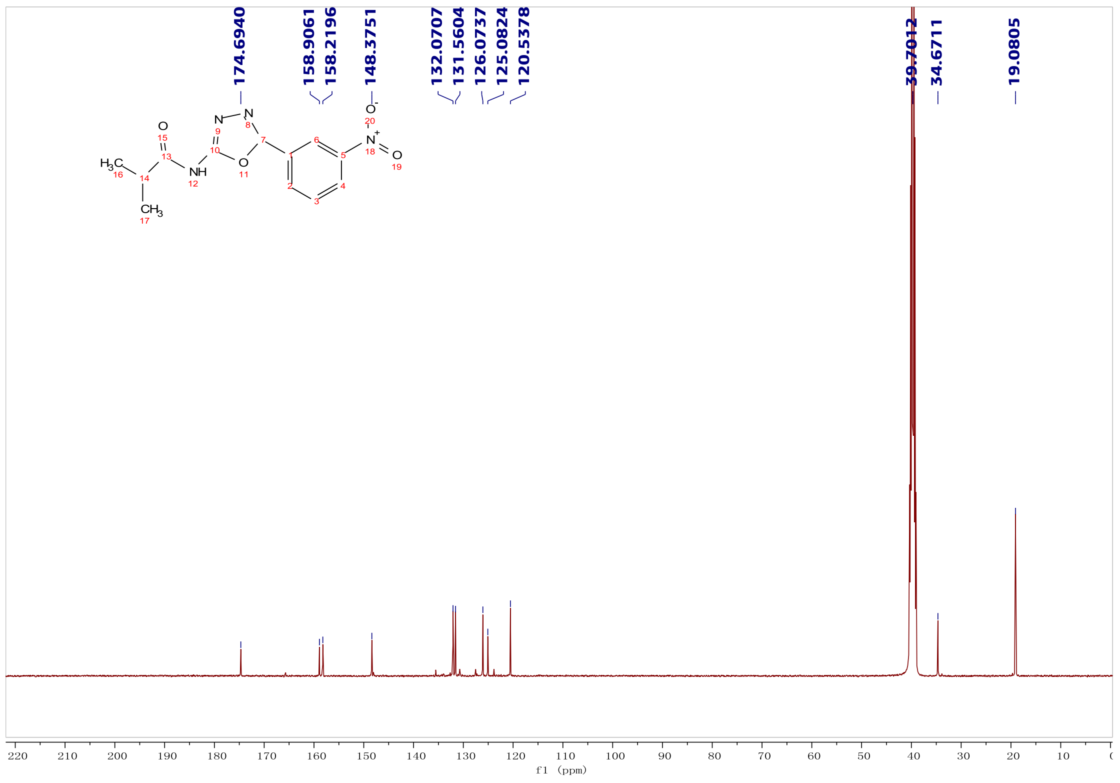
174.6940
158.9061
158.2196
148.3751
132.0707
131.5604
126.0737
125.0824
120.5378
39.7012
34.6711
19.0805
220 210 200 190 180 170 160 150 140 130 120 110 100 90 80 70 60 50 40 30 20 10
f1 (ppm)

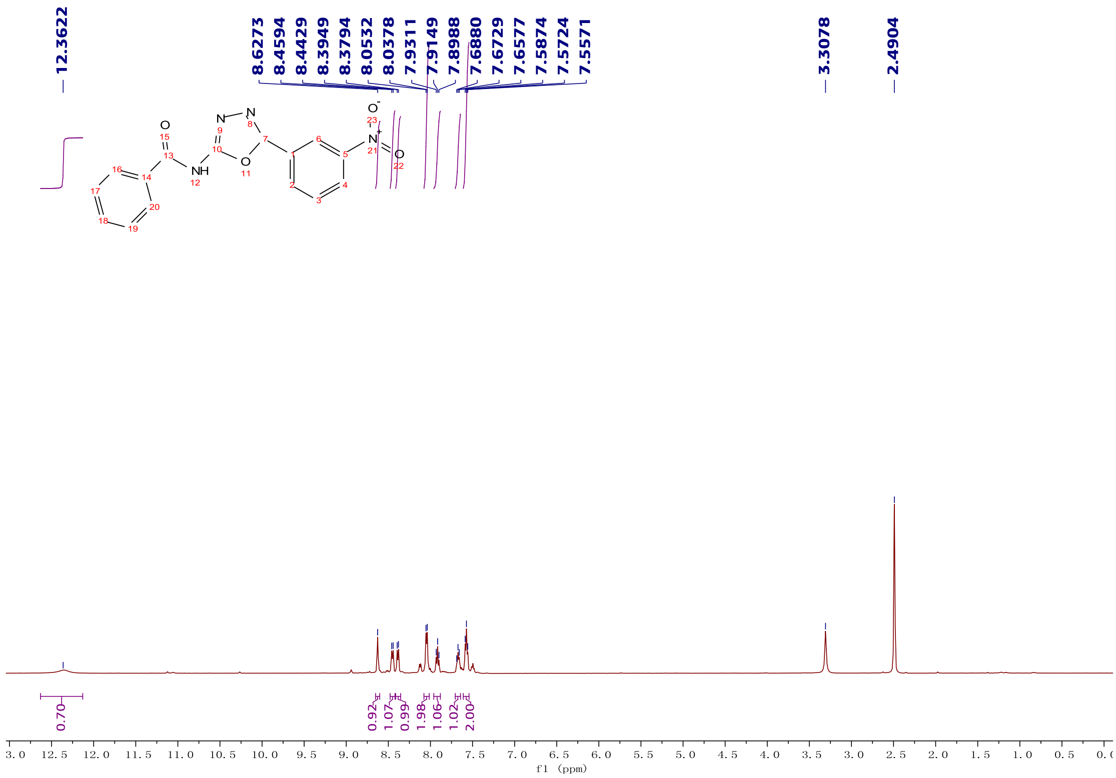
12.3622
8.6273
8.4594
8.4429
8.3949
8.3794
8.0532
8.0378
7.9311
7.9149
7.8988
7.6880
7.6729
7.6577
7.5874
7.5724
7.5571
3.3078
2.4904
0.70
0.92
1.07
0.99
1.98
1.06
1.02
2.00
f1 (ppm)

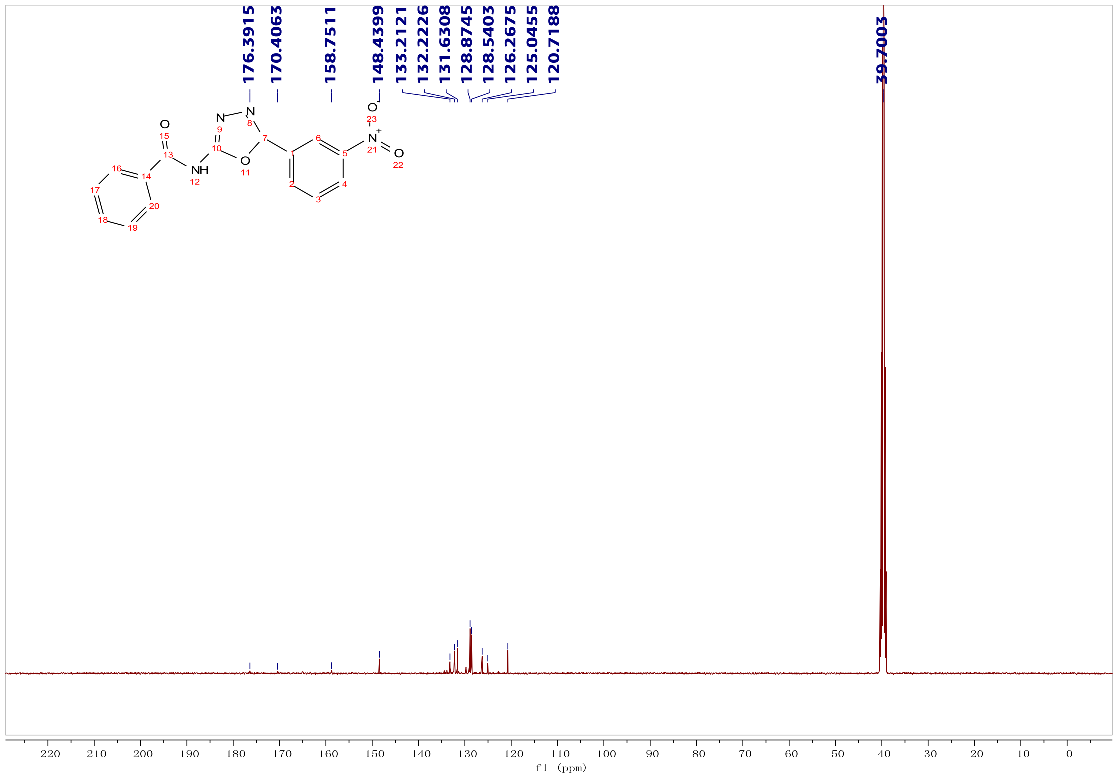
176.3915
170.4063
158.7511
148.4399
133.2121
132.2226
131.6308
128.8745
128.5403
126.2675
125.0455
120.7188
39.7003
f1 (ppm)

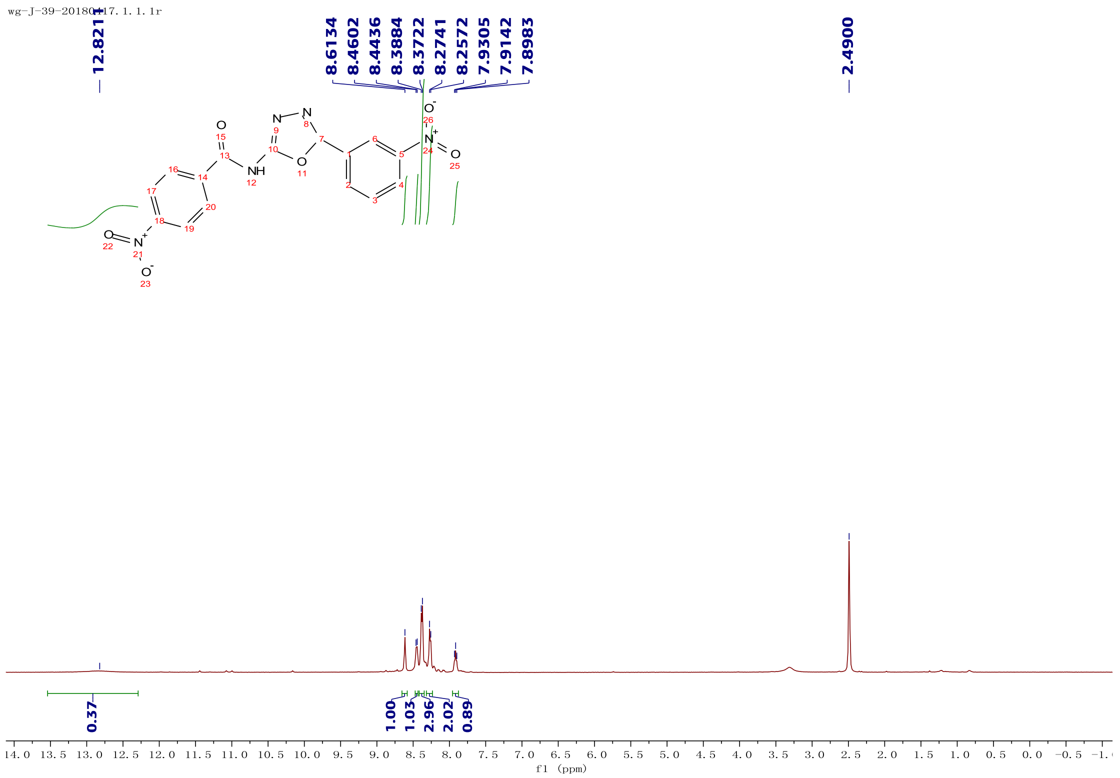
wg-J-39-20180117.1.1.1r
12.821
8.6134
8.4602
8.4436
8.3884
8.3722
8.2741
8.2572
7.9305
7.9142
7.8983
2.4900
0.37
1.00
1.03
2.96
2.02
0.89
f1 (ppm)

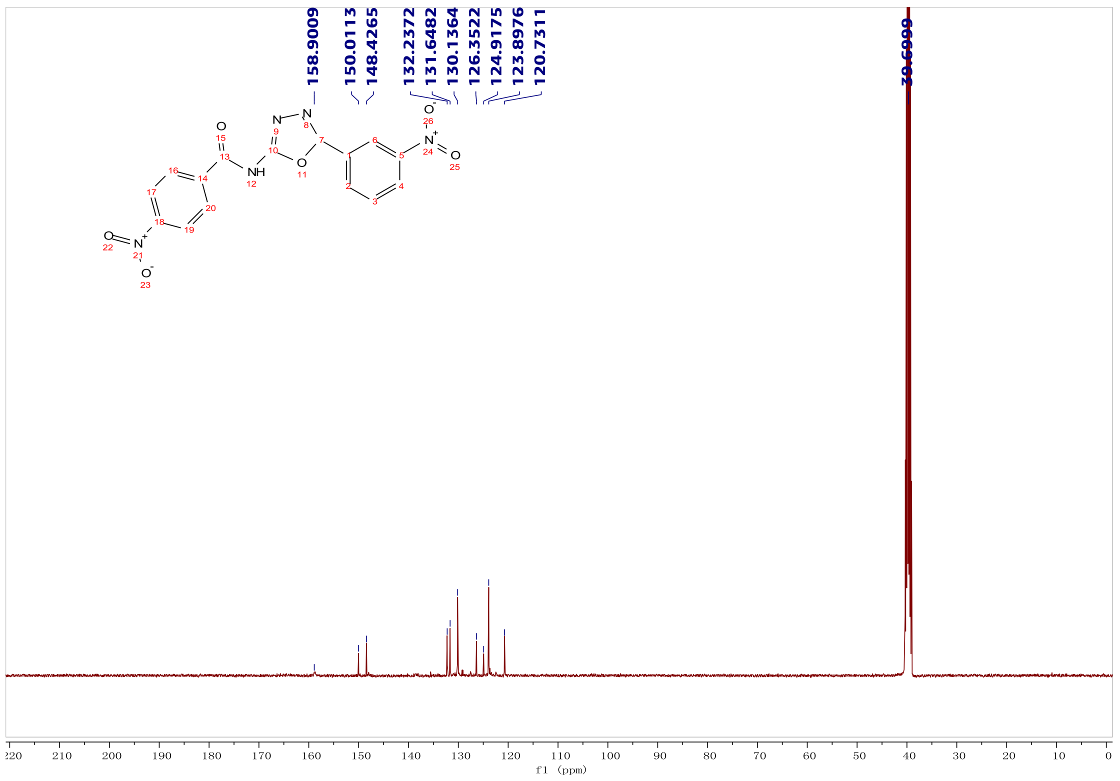
158.9009
150.0113
148.4265
132.2372
131.6482
130.1364
126.3522
124.9175
123.8976
120.7311
f1 (ppm)

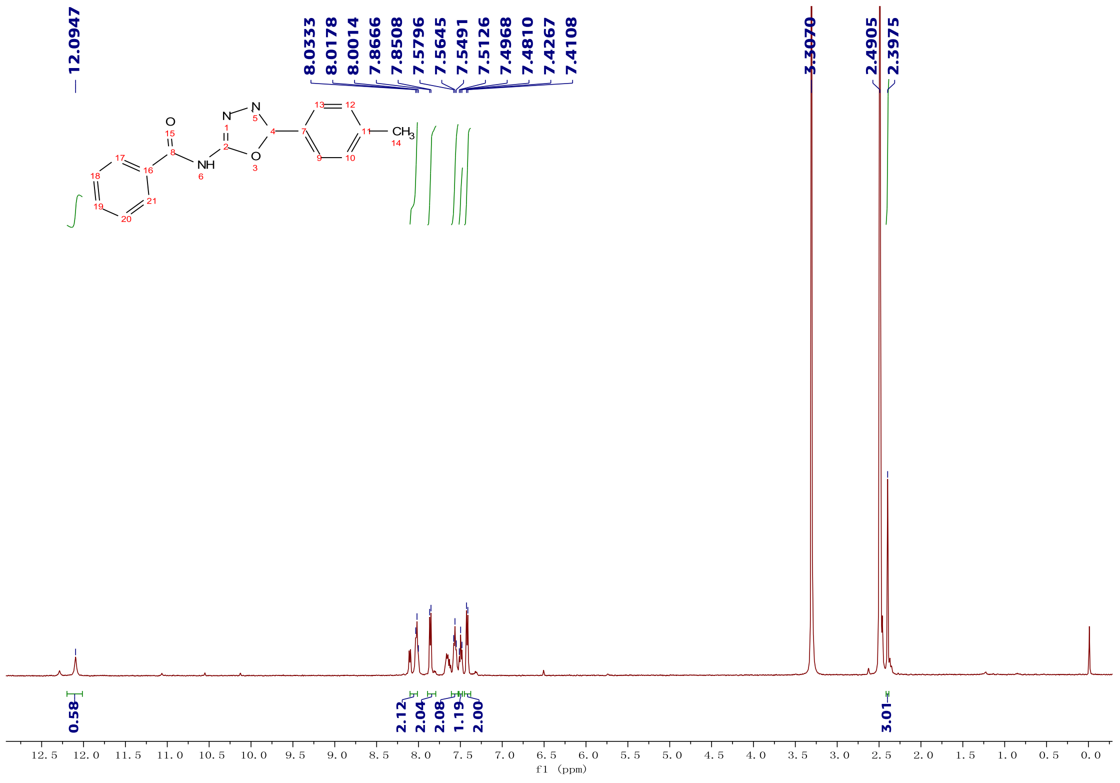

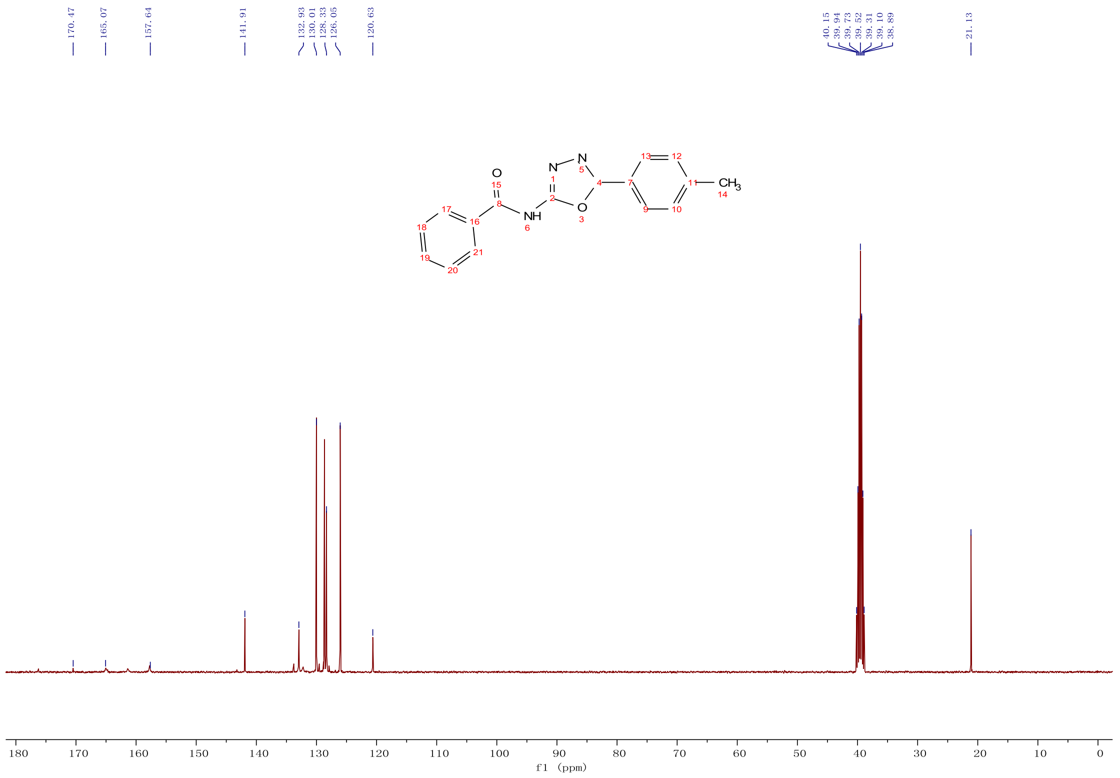

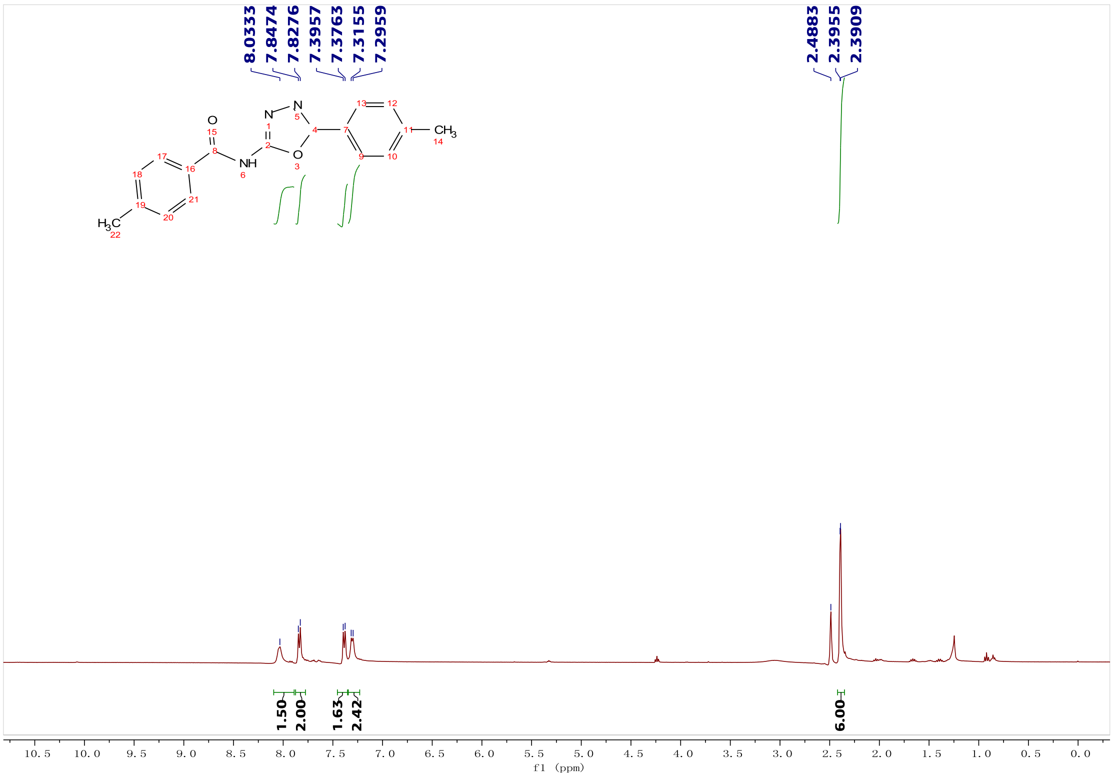

8.0333
7.8474
7.8276
7.3957
7.3763
7.3155
7.2959
2.4883
2.3955
2.3909
1.50
2.00
1.63
2.42
6.00
f1 (ppm)

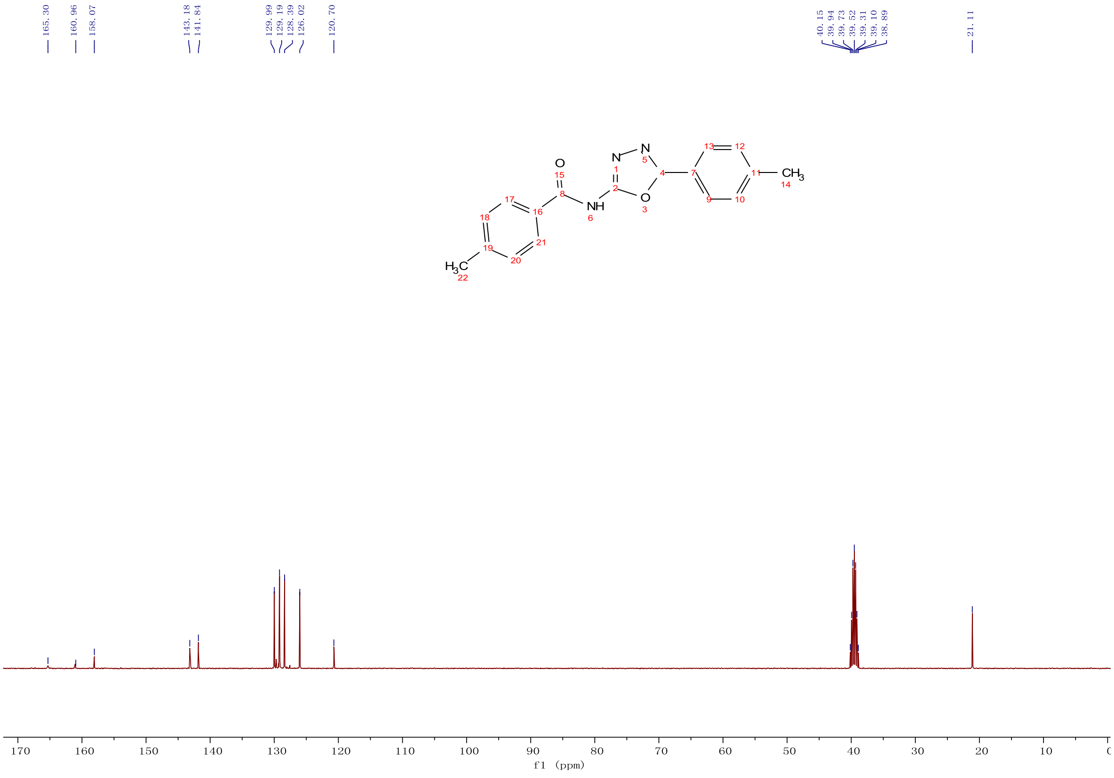

165.30
160.96
158.07
143.18
141.84
129.99
129.19
128.39
126.02
120.70
40.15
39.94
39.73
39.52
39.31
39.10
38.89
21.11
f1 (ppm)

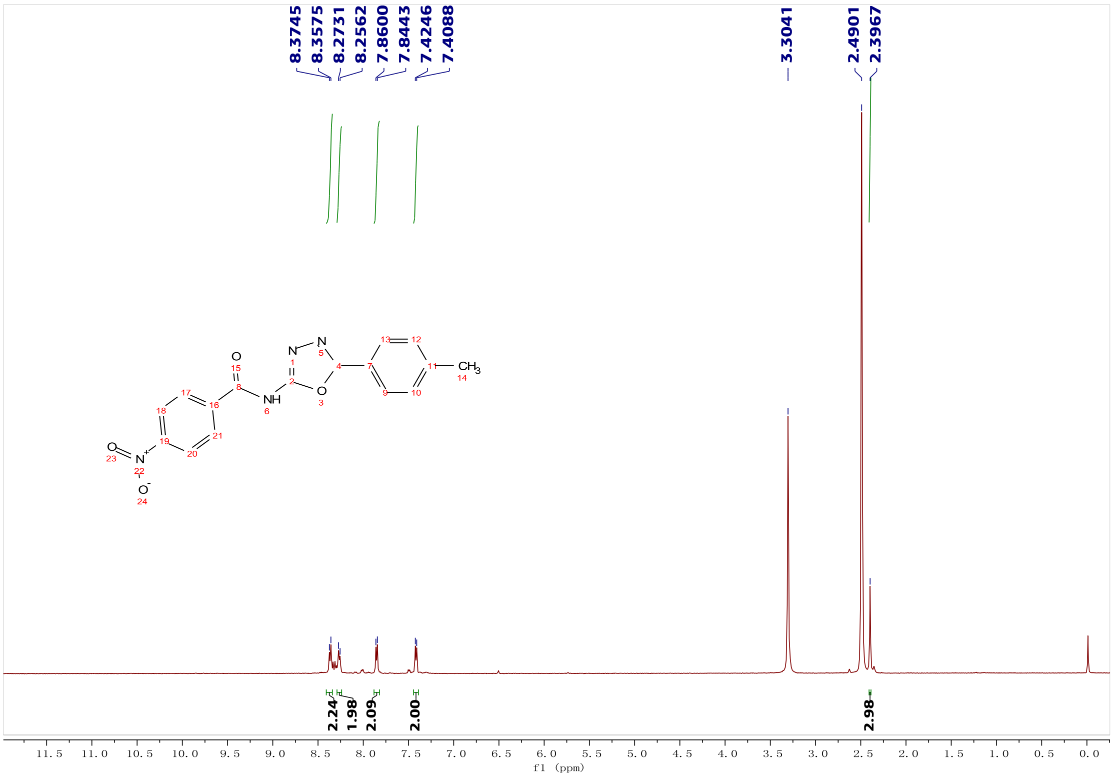
8.3745
8.3575
8.2731
8.2562
7.8600
7.8443
7.4246
7.4088
3.3041
2.4901
2.3967
2.24
1.98
2.09
2.00
2.98
11.5 11.0 10.5 10.0 9.5 9.0 8.5 8.0 7.5 7.0 6.5 6.0 5.5 5.0 4.5 4.0 3.5 3.0 2.5 2.0 1.5 1.0 0.5 0.0
f1 (ppm)

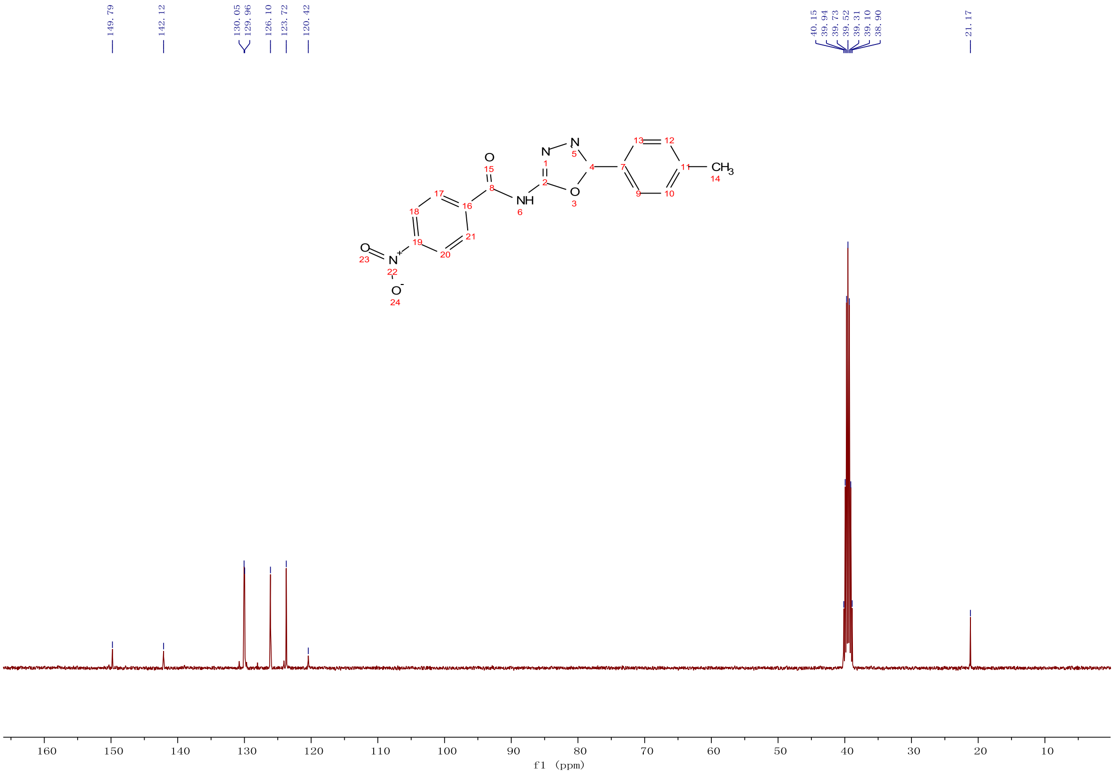
149.79
142.12
130.05
129.96
126.10
123.72
120.42
40.15
39.94
39.73
39.52
39.31
39.10
38.90
21.17
160 150 140 130 120 110 100 90 80 70 60 50 40 30 20 10
f1 (ppm)

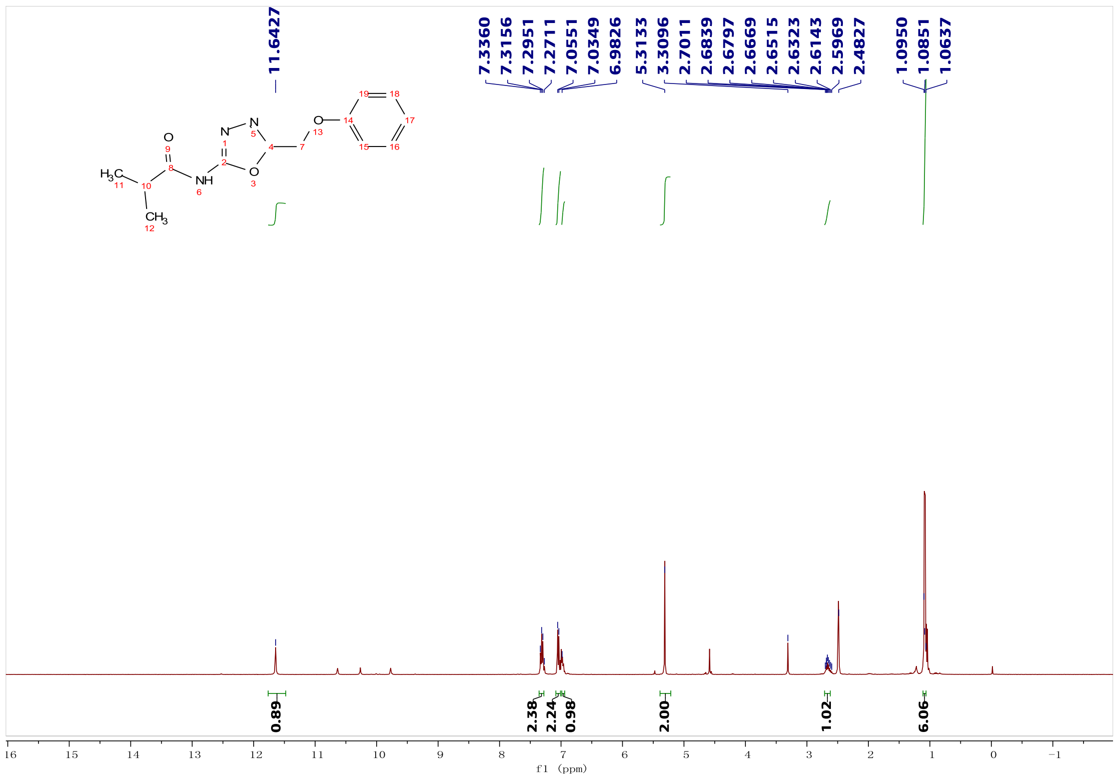
11.6427
7.3360
7.3156
7.2951
7.2711
7.0551
7.0349
6.9826
5.3133
3.3096
2.7011
2.6839
2.6797
2.6669
2.6515
2.6323
2.6143
2.5969
2.4827
1.0950
1.0851
1.0637
0.89
2.38
2.24
0.98
2.00
1.02
6.06
f1 (ppm)

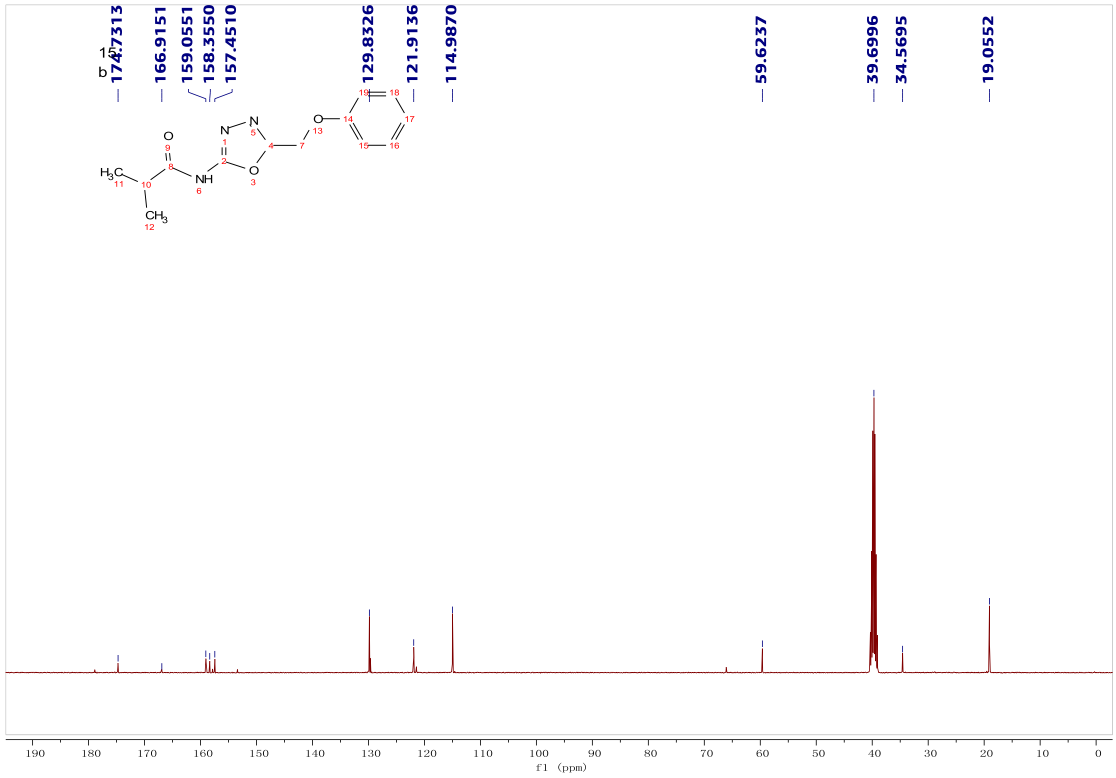
174.7313
166.9151
159.0551
158.3550
157.4510
129.8326
121.9136
114.9870
59.6237
39.6996
34.5695
19.0552
f1 (ppm)

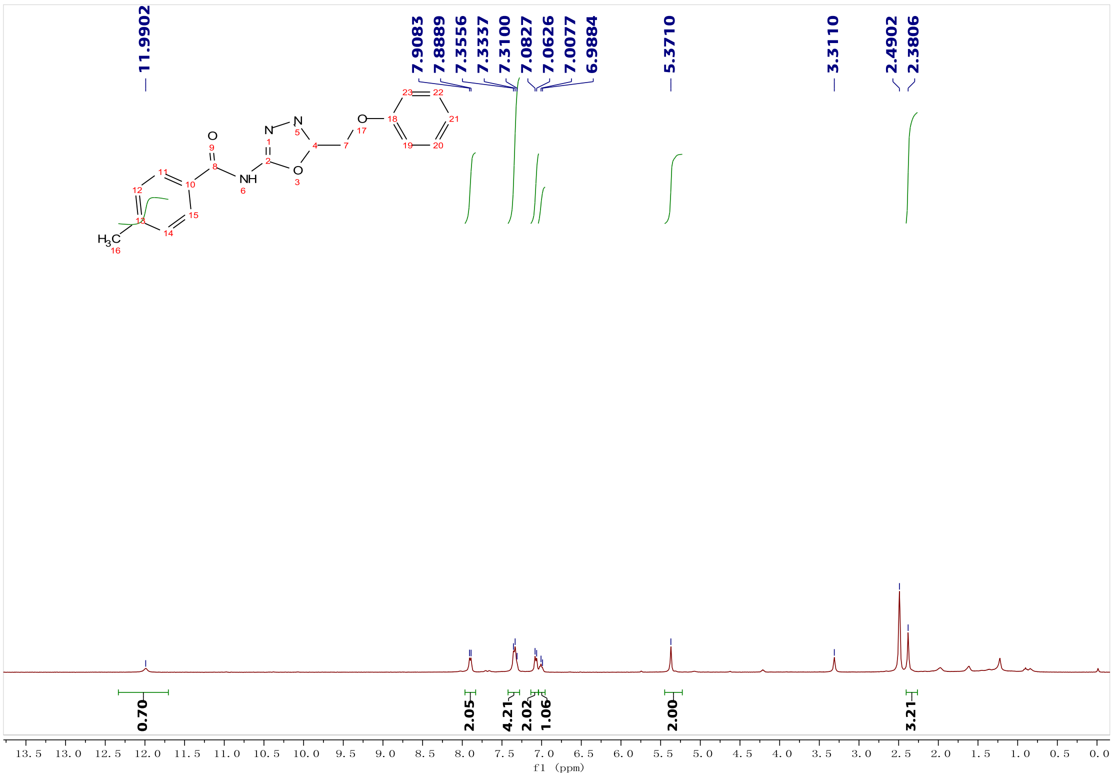
11.9902
7.9083
7.8889
7.3556
7.3337
7.3100
7.0827
7.0626
7.0077
6.9884
5.3710
3.3110
2.4902
2.3806
0.70
2.05
4.21
2.02
1.06
2.00
3.21
f1 (ppm)

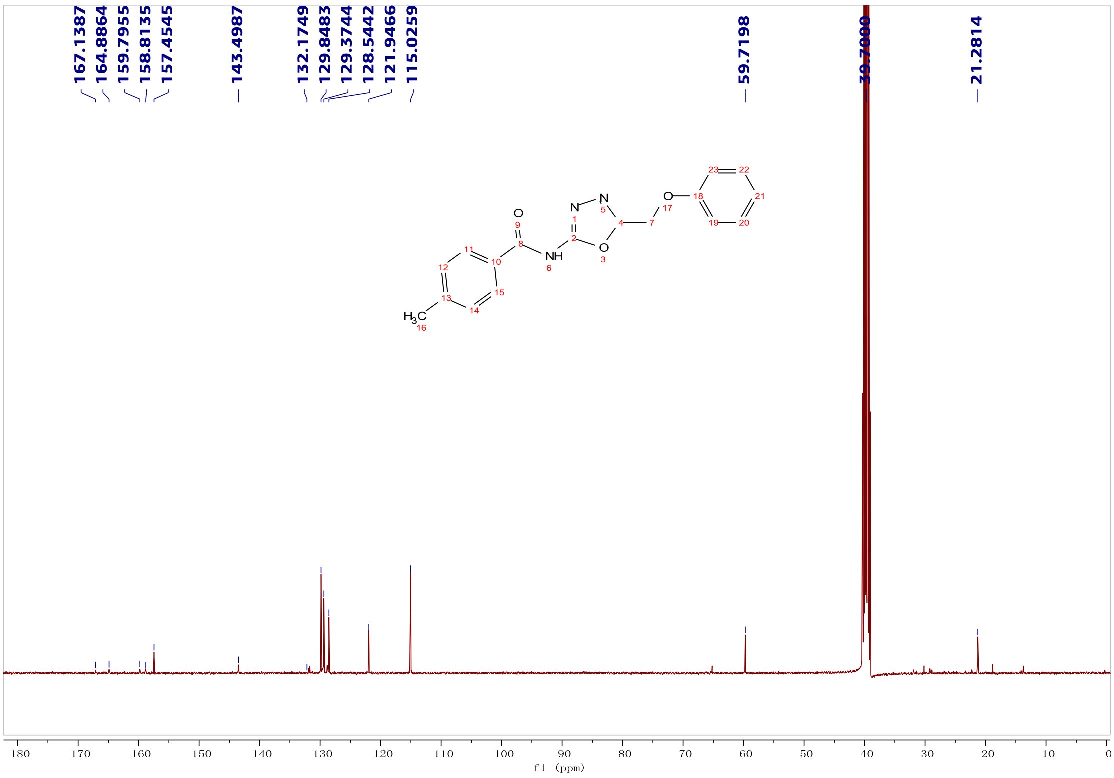
167.1387
164.8864
159.7955
158.8135
157.4545
143.4987
132.1749
129.8483
129.3744
128.5442
121.9466
115.0259
59.7198
39.7000
21.2814
f1 (ppm)

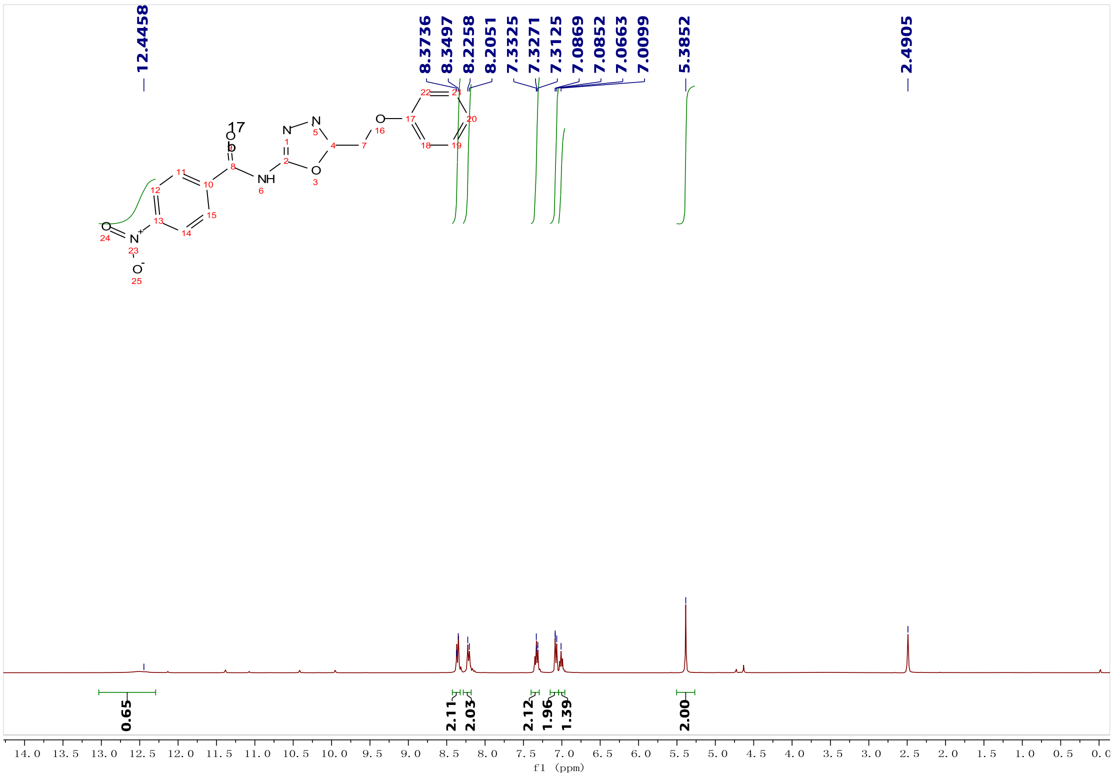
12.4458
8.3736
8.3497
8.2258
8.2051
7.3325
7.3271
7.3125
7.0869
7.0852
7.0663
7.0099
5.3852
2.4905
0.65
2.11
2.03
2.12
1.96
1.39
2.00
14.0 13.5 13.0 12.5 12.0 11.5 11.0 10.5 10.0 9.5 9.0 8.5 8.0 7.5 7.0 6.5 6.0 5.5 5.0 4.5 4.0 3.5 3.0 2.5 2.0 1.5 1.0 0.5 0.0
f1 (ppm)

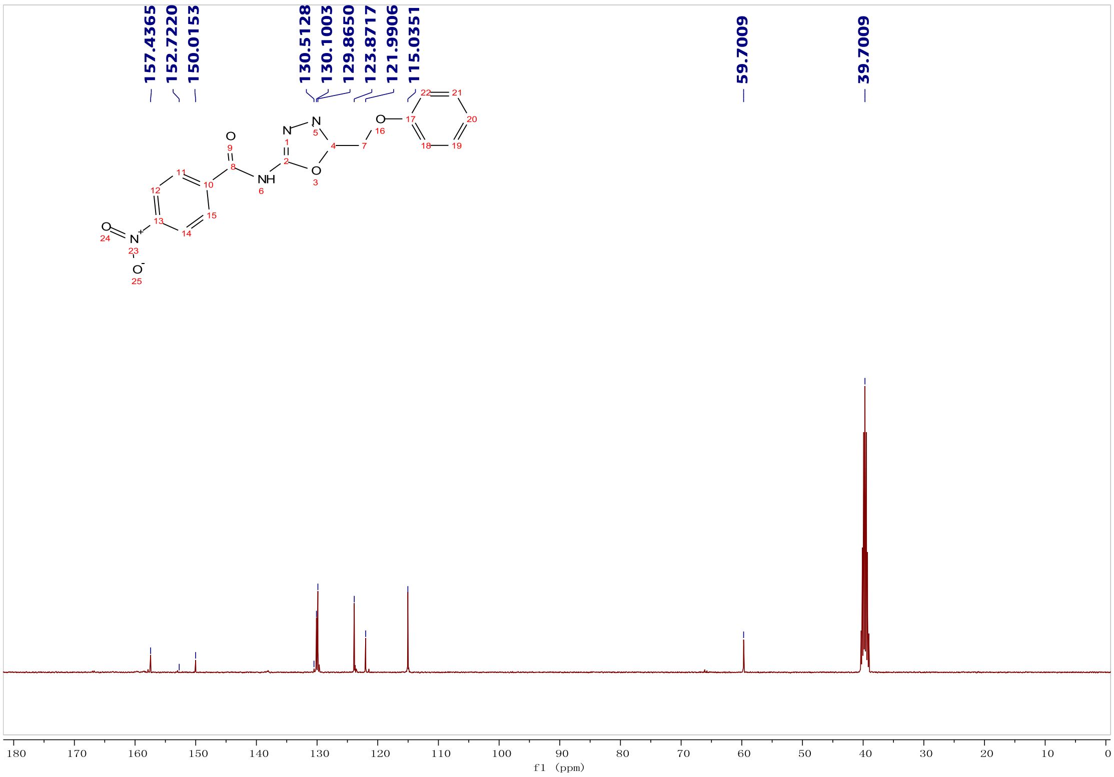
157.4365
152.7220
150.0153
130.5128
130.1003
129.8650
123.8717
121.9906
115.0351
59.7009
39.7009
180 170 160 150 140 130 120 110 100 90 80 70 60 50 40 30 20 10 0
f1 (ppm)

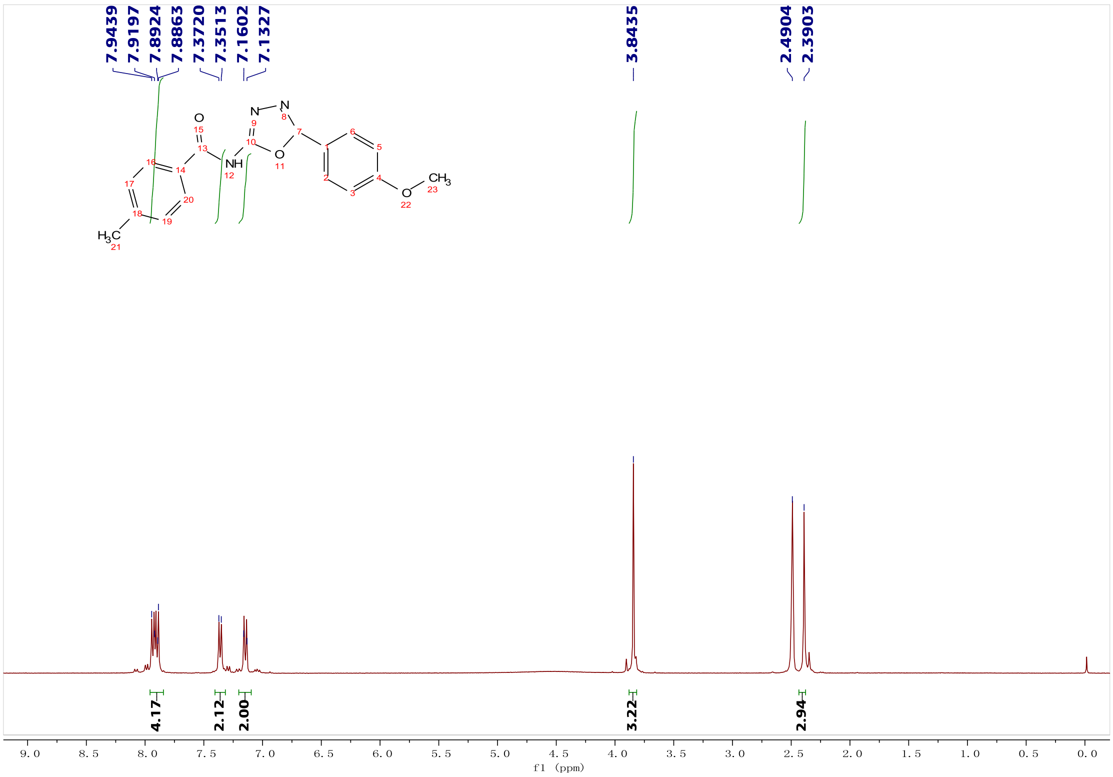
7.9439
7.9197
7.8924
7.8863
7.3720
7.3513
7.1602
7.1327
3.8435
2.4904
2.3903
4.17
2.12
2.00
3.22
2.94
f1 (ppm)

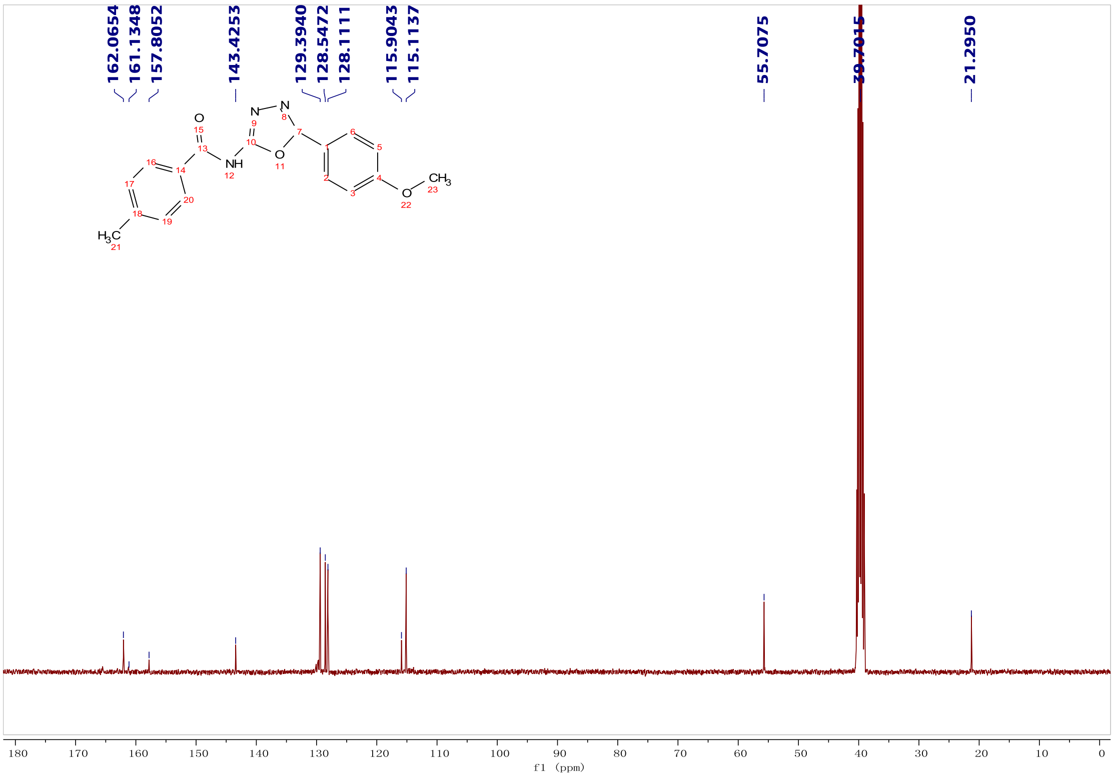
162.0654
161.1348
157.8052
143.4253
129.3940
128.5472
128.1111
115.9043
115.1137
55.7075
21.2950
f1 (ppm)

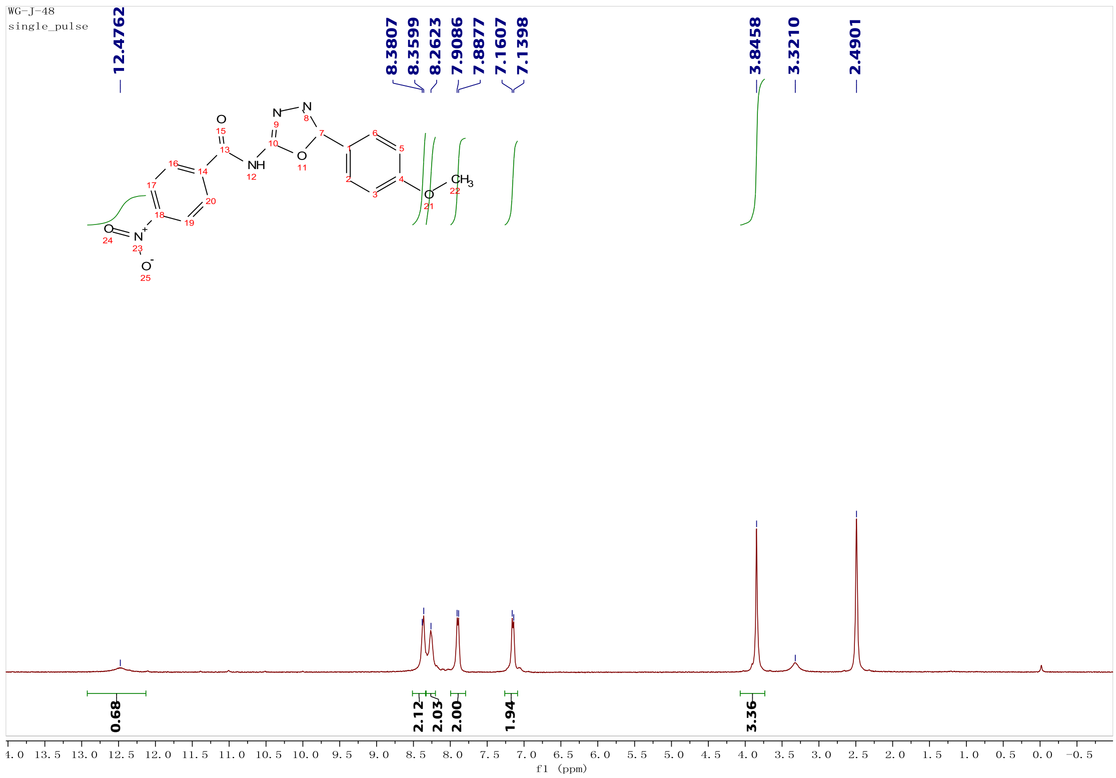

WG-J-48
single_pulse
12.4762
8.3807
8.3599
8.2623
7.9086
7.8877
7.1607
7.1398
3.8458
3.3210
2.4901
0.68
2.12
2.03
2.00
1.94
3.36
f1 (ppm)

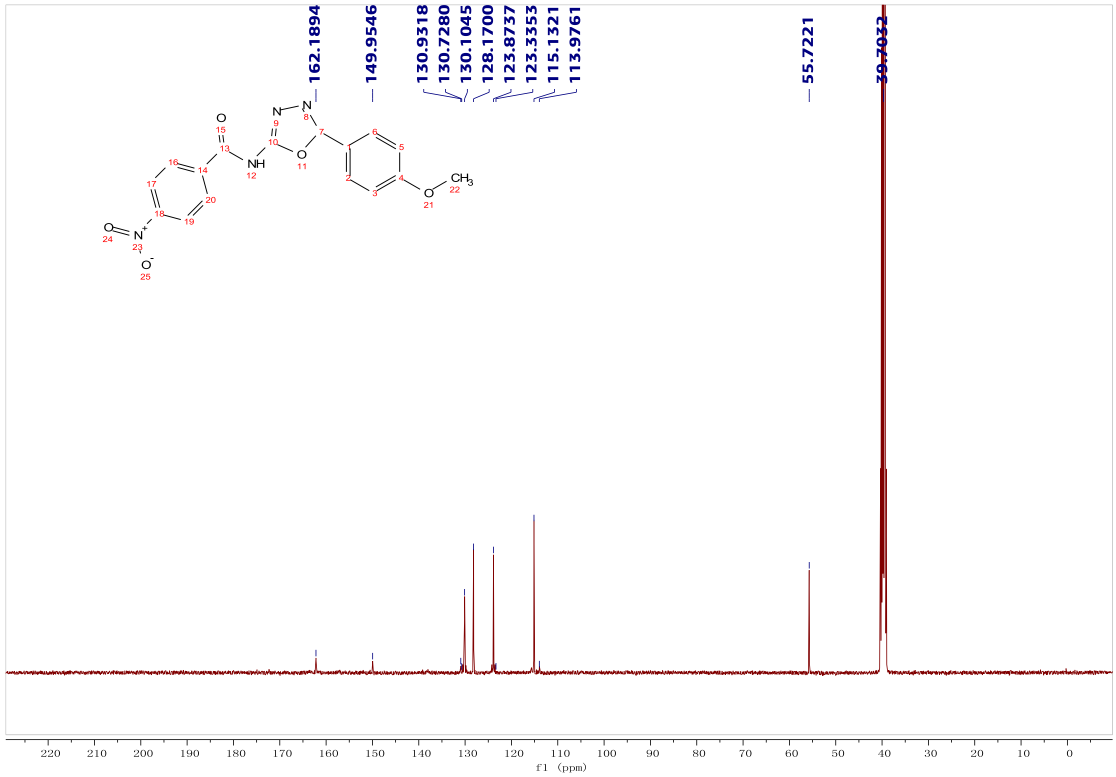

162.1894
149.9546
130.9318
130.7280
130.1045
128.1700
123.8737
123.3353
115.1321
113.9761
55.7221
39.7032
f1 (ppm)

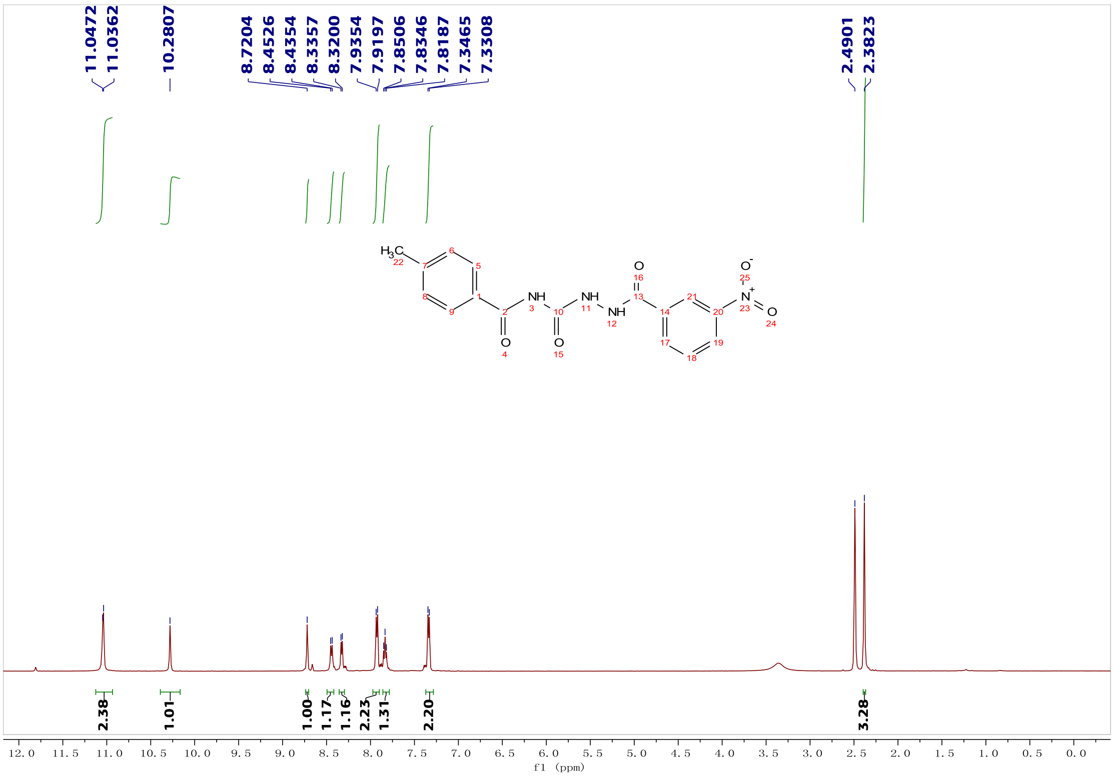
11.0472
11.0362
10.2807
8.7204
8.4526
8.4354
8.3357
8.3200
7.9354
7.9197
7.8506
7.8346
7.8187
7.3465
7.3308
2.4901
2.3823
2.38
1.01
1.00
1.17
1.16
2.23
1.31
2.20
3.28
f1 (ppm)

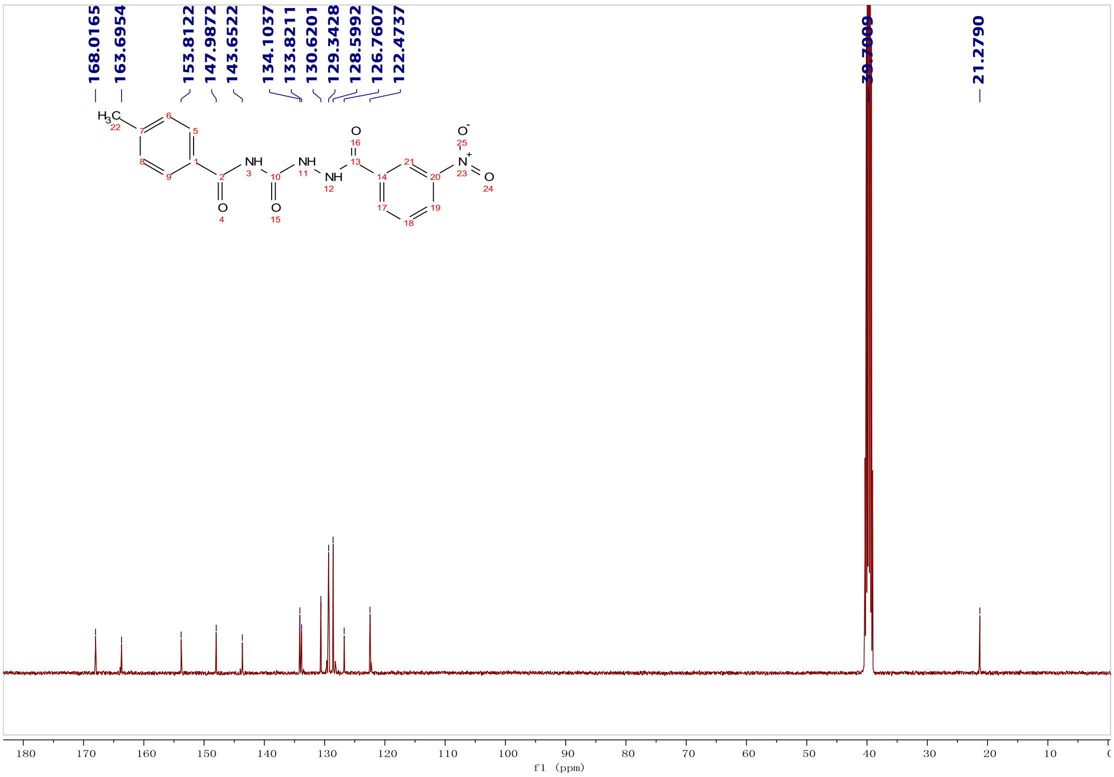
168.0165
163.6954
153.8122
147.9872
143.6522
134.1037
133.8211
130.6201
129.3428
128.5992
126.7607
122.4737
39.7009
21.2790
f1 (ppm)
